# Machine learning designs non-hemolytic antimicrobial peptides†

**DOI:** 10.1039/d1sc01713f

**Published:** 2021-06-07

**Authors:** Alice Capecchi, Xingguang Cai, Hippolyte Personne, Thilo Köhler, Christian van Delden, Jean-Louis Reymond

**Affiliations:** Department of Chemistry, Biochemistry and Pharmaceutical Sciences, University of Bern Freiestrasse 3 3012 Bern Switzerland jean-louis.reymond@dcb.unibe.ch; Department of Microbiology and Molecular Medicine, University of Geneva Switzerland; Service of Infectious Diseases, University Hospital of Geneva Geneva Switzerland

## Abstract

Machine learning (ML) consists of the recognition of patterns from training data and offers the opportunity to exploit large structure–activity databases for drug design. In the area of peptide drugs, ML is mostly being tested to design antimicrobial peptides (AMPs), a class of biomolecules potentially useful to fight multidrug-resistant bacteria. ML models have successfully identified membrane disruptive amphiphilic AMPs, however mostly without addressing the associated toxicity to human red blood cells. Here we trained recurrent neural networks (RNN) with data from DBAASP (Database of Antimicrobial Activity and Structure of Peptides) to design short non-hemolytic AMPs. Synthesis and testing of 28 generated peptides, each at least 5 mutations away from training data, allowed us to identify eight new non-hemolytic AMPs against *Pseudomonas aeruginosa*, *Acinetobacter baumannii*, and methicillin-resistant *Staphylococcus aureus* (MRSA). These results show that machine learning (ML) can be used to design new non-hemolytic AMPs.

## Abstract 2

Machine learning models trained with experimental data for antimicrobial activity and hemolysis are shown to produce new non-hemolytic antimicrobial peptides active against multidrug-resistant bacteria.

## Graphical abstract

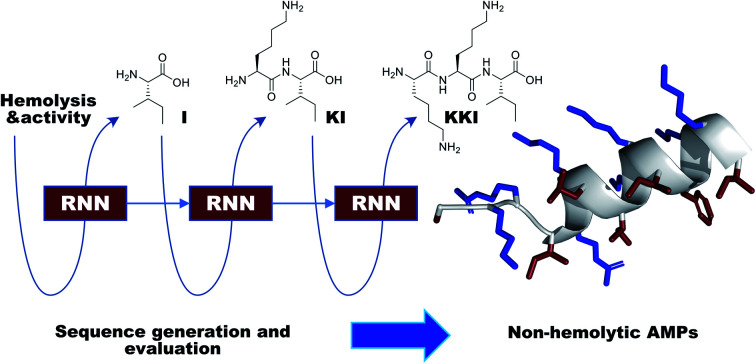

## 1 Introduction

Machine learning (ML) is a part of artificial intelligence consisting of using algorithms to recognize patterns in training data. In the context of computer-aided drug discovery,^1,2^ ML allows one to exploit experimental structure–activity data on known drugs to generate new molecules and predict their properties and activities.^3–5^ Generating new molecules is commonly a two-step approach that requires first a more general training and then a fine-tuning towards a specific set of characteristics. The fine-tuning of a generative ML model can be achieved with transfer learning (TL), which is essentially a second learning of a prior generative model with a smaller set of compounds.^6^

In the area of computational peptide design,^7,8^ ML models for generation and activity classification can readily be trained with structure–activity data using the linear sequence of amino acids as input for the peptide structure. Efforts to develop and test ML for peptide design mostly focus on antimicrobial peptides (AMPs)^9,10^ because relatively large structure–activity databases are available in the public domain.^11–17^ Antimicrobial peptides (AMPs) are synthesized by microorganisms, plants, and animals as a defense against bacterial predators innate immunity. They often show good activity against multidrug-resistant bacteria, thereby offering an opportunity to address this global public health threat.^18–21^

Most AMPs are polycationic and act by disrupting bacterial membranes, usually by folding into an amphiphilic α-helix at the membrane surface,^22,23^ a mechanism against which resistance is not easily obtained and which has been used broadly to guide the design of new AMPs. Unfortunately, designing amphiphilicity often results in compounds lacking selectivity against eukaryotic membranes and showing hemolytic properties, which strongly limits their use.^24^ In principle, ML should be optimally suited to address this challenge by training models with data on AMPs with annotated hemolysis data.

Several ML models for AMP *de novo* design have been reported so far, and they range from classifiers for AMPs prediction applied to select sequences from randomly generated, existing, or genome derived libraries,^25–33^ to standalone generative models,^34^ to a combination of both generative models and classifiers.^35–37^ Furthermore, ML has also been used in combination with evolutionary algorithms for the optimization of AMPs.^38,39^ However, only two of the discussed studies considered both activity and hemolysis in the design of novel AMPs,^31,37^ reflecting the challenge of avoiding hemolysis in designing AMPs, and highlighting the importance of its further investigation.

Here we considered the use of ML for AMP design considering activity and hemolysis by training our models on sets of active, inactive, hemolytic, and non-hemolytic sequences derived from reported activity data. We also aimed to validate if ML can be used to identify new AMPs by testing only sequences substantially different from known AMPs. Starting with sequence information and antimicrobial and hemolysis data from DBAASP (Database of Antimicrobial Activity and Structure of Peptides),^12^ which contains manually curated information on activity values and hemolysis behavior, we trained a combination of generative and predictive recurrent neural networks (RNN). To generate peptide sequences, we trained a generative model and we fine-tuned it using TL to target three problematic and often drug-resistant pathogens: the Gram-negative *Pseudomonas aeruginosa* and *Acinetobacter baumannii,* and the Gram-positive *Staphylococcus aureus*. Furthermore, to select non-hemolytic AMPs among the generated sequences, we implemented two RNN classifiers to predict antimicrobial activity and hemolysis. Our combination of supervised and unsupervised learning to design non-hemolytic AMPs is unprecedented, and it allowed us to maximizes the use of highly curated data on antimicrobial activity and hemolysis. Synthesis and testing of twenty-eight of the generated and selected sequences resulted in twelve new active AMPs, eight of which were also non-hemolytic. Detailed characterization of the best two peptides showed that they are typical α-helical membrane disruptive AMPs.

## 2 Results and discussion

### 2.1 Machine learning

#### 2.1.1 DBAASP

DBAASP contains peptides annotated with activity values, and when known, with their hemolytic behavior. This allowed us to obtain reliable AMP activity and hemolysis data. With a threshold of 32 μg mL^−1^ and 10 μM, we identified 4774 active and 1867 inactive linear peptides. Additionally, we considered the DBAASP peptides reported to cause less than 20% hemolysis at a concentration of at least 50 μM as non-hemolytic and the peptides reported to cause more than 20% hemolysis at any concentration as hemolytic, which resulted in 1319 hemolytic and 943 non-hemolytic linear peptide sequences. Finally, we extracted 339 non-hemolytic peptides active against the Gram-negative bacteria *P. aeruginosa* and/or *A. baumannii* and 458 non-hemolytic peptides active against the Gram-positive bacterium *S. aureus.*

#### 2.1.2 Generative models

**Fig. 1:**
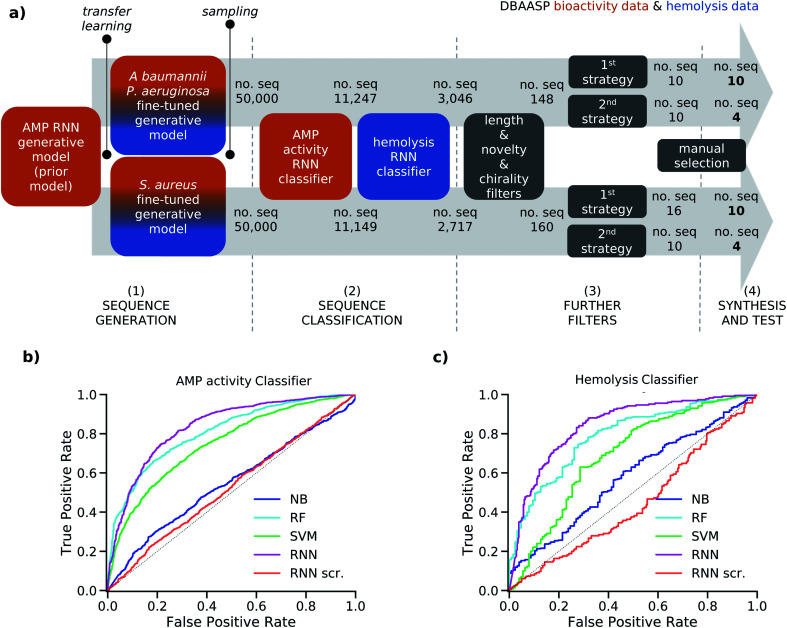
(a) Strategy schematic. An AMP RNN generative model, an AMP RNN activity classifier, and a hemolysis RNN classifier were trained using activity (orange) and hemolysis (blue) data from DBAASP. (1) Two copies of the AMP RNN generative model (prior model) were transferred learned using active and non-hemolytic peptides against specific strains: *P. aeruginosa*/*A. baumannii* and *S. aureus*, respectively. (2) The fine-tuned models were sampled, and the generated sequences were first classified using the RNN AMP activity classifier and then the RNN hemolysis classifier. (3) The selected sequences were further filtered to obtain short peptides of maximum 15 residues with at least five mutations from the sequences in DBAASP and no d amino acids. Then two different selection strategies were used. In the first selection strategy (1^st^ strategy) we used the calculated amphiphilicity of the sequences to further filter them, and we clustered the selected ones. In the second selection strategy (2^nd^ strategy) we select at random 10 sequences. (4) Finally, the 28 chosen sequences were synthesized and tested. (b) ROC curves of the test set for the NB, RF, SVM, RNN, and RNN with scrambled labels (RNN scr.) models for the AMP activity (b) and hemolysis (c) classification tasks. The probabilistic prediction values were converted into binary classification values using a threshold of 0.5.

Alone, the 339 and 458 non-hemolytic AMPs active, respectively, against *P. aeruginosa* and/or *A. baumannii* and *S. aureus* are not enough to directly train a generative model able to design a diverse set of novel AMPs. To overcome the challenge posed by the scarcity of data points on specific strains in the DBAASP, we first trained a general generative model on the entire DBAASP, and then we fine-tuned it with the smaller subset of AMPs with reported hemolysis data and specific activity (Fig. 1a). The 4774 active peptides in the DBAASP were divided into a training and a test set, and the training set was used to train an RNN generative model to produce AMPs (prior model). Subsequently, two generative models were derived by fine-tuning the prior model with TL using two smaller sets of sequences with a specific activity and known non-hemolytic behavior: (i) the 242 non-hemolytic peptide sequences present in the training set of the prior model and active against the Gram-negative *P. aeruginosa* and/or *A. baumannii* and (ii) the 321 non-hemolytic sequences present in the training set of the prior model and active against the Gram-positive *S. aureus*. Interestingly, 170 peptides were common to both sets (see Methods Sections 4.1 and 4.5 for details).

To avoid overfitting, the prior and the two fine-tuned generative models were trained with the respective training sets until the probability of generating the related test sets reached their maximum value. For the fined-tuned models, the 97 and 137 sequences active, respectively, against *P. aeruginosa*/*A. baumannii* and *S. aureus*, which were present in the test set of the prior model, were used as test set. We then sampled 50 000 peptide sequences from each of the two fine-tuned models. The percentage of unique sampled sequences was 82.8% for the *P. aeruginosa*/*A. baumannii* model and 82.3% for the *S. aureus* model. Furthermore, in both cases over 99% of the sampled sequences were not present in the corresponding training set used for transfer learning due to our attention in avoiding overfitting. The high percentage of uniqueness and the novelty of the generated sequences within the 50 000 samples showed that our fine-tuned models were capable of generating new and diverse sequences. This allowed us to proceed in our analysis with a relatively small and manageable number of candidate peptide sequences.

#### 2.1.3 Classifiers

To assess the capabilities of the prior model and to predict the AMP activity of the generated peptide sequences, we implemented a NB (Naive Bayes), an SVM (Support Vector Model), a RF (Random Forest), and an RNN AMP activity classifiers. The DBAASP active compounds in the same training/test split used for the prior model were used as positive class. As negative class, we used an equally sized set of inactive sequences dived in training and test sets. The inactive sequences consisted of all inactive sequences in DBAASP and additional sequences generated by scrambling active peptides and by fragmenting SwissProt entries. As a baseline and to make sure that the performance of the RNN model was due to a trend in the data and not to an artifact, an RNN activity classifier with the same architecture but trained with scrambled labels was implemented. The models were trained using the training set, and their performances were evaluated using the test set (Fig. 1b and Table S1†). The RNN activity classifier performed best across all computed metrics (ROC AUC = 0.84, accuracy = 0.76, precision = 0.74, recall = 0.80, F1 score = 0.77, MCC = and 0.53) and was selected for further investigation.

To account for non-hemolytic behavior, a second classifier to distinguish between hemolytic and non-hemolytic sequences was trained. In this case, the DBAASP entries with hemolysis annotation were used to train the models. Non-hemolytic sequences were considered as the positive class and hemolytic sequences as the negative class. Being the sequences with hemolysis data a subset of the ones having activity data, we used the same training/test split used for the activity classifier (for details refer to method Section 4.1). Similar to the AMP activity classification discussed above, an RNN classifier with scrambled labels (baseline), NB, SVM, RF, and RNN classifiers were trained with the training set and evaluated for the hemolysis task with the test set. As for the activity classifier discussed above, the RNN classifier had the best overall performance for hemolysis prediction (ROC AUC = 0.87, accuracy = 0.76, precision = 0.70, recall = 0.76, F1 score = 0.73, MCC = 0.52) and was selected for further study (Fig. 1c and Table S1†).

To increase the precision of the RNN AMP activity and RNN hemolysis classifiers, we raised the threshold used to transform their probabilistic output to a binary classification from 0.5 to over 0.95 for both classifiers (refer to Methods 4.6 for details). This resulted in an adjusted precision of 0.91 and 0.84 for the RNN AMP activity classifier and the RNN hemolysis classifier, respectively. Therefore, when considering the antimicrobial activity and the hemolysis behavior of a peptide sequence as two independent characteristics, we obtained a combined precision of 0.76, which means that 76% of predicted positives are expected to have antimicrobial activity and non-hemolytic properties. However, because hemolysis is a known drawback of antimicrobial peptides, non-hemolytic behavior and antimicrobial activity are likely to be inversely proportional. This is also evident when looking at the 1786 active peptides reported in the DBAASP with a hemolysis annotation, as only 721 are reported as non-hemolytic. For this reason, a lower overall performance of the two classifiers was expected.

#### 2.1.4 Sequences selection

The RNN AMP activity and hemolysis classifiers were used to filter the 50 000 sequences sampled from each of the two fine-tuned generative models, resulting in 3046 sequences from the model fine-tuned for *P. aeruginosa* and *A. baumannii* and 2717 from the model fine-tuned for *S. aureus* (Fig. 1a). To facilitate the synthesis process, sequences longer than 15 amino acids were excluded (Fig. S1a†). The sequences were further filtered to ensure novelty, considering a minimum of four mutations from the test set peptides and, to further challenge our model, of five mutations from the training set peptides (Fig. S1b to e†). This selection criterion has not been used in previous AMP discovery approaches using ML, however, we believe it to be fundamental to avoid trivial analogs of known peptides and analogs which have already been studied within SAR analysis. Finally, we decided to exclude sequences containing d amino acids since the percentage of d amino acids in the training sets of the generative model and of the classifiers was too low for the model to learn this feature (Fig. S1f†). The selection yielded 148 and 160 peptides from the *P. aeruginosa*/*A. baumannii* model and *S. aureus* model, respectively.

Then, two different strategies to further select the sequences were followed. In the first case, we used the calculated hydrophobic moment^40^ and the predicted α-helix fraction as estimations of amphiphilic helix to further filter the sequences (Fig. S1g†) and performed clustering to diversify our selection (first selection strategy). In the second case, we randomly sampled 10 sequences out of each pool of peptides to follow the model sampling distribution (second selection strategy, see Methods Section 4.7 for details). This selection resulted in 20 peptide sequences from the *P. aeruginosa*/*A. baumannii* model and 26 peptide sequences from the *S. aureus* model. From each set, 14 peptides were chosen manually for experimental evaluation. Thanks to the applied filters and selection processes, all selected sequences were distinct from the training and test sets of both AMP activity and hemolysis classifiers in at least five positions, and to the best of our knowledge, they were not present in any peptide databases. The sequences coming from the *P. aeruginosa*/*A. baumannii* model were labeled as Gram-negative targeting compounds (GN), and the sequences selected from the *S. aureus* model were labeled as Gram-positive targeting compounds (GP).

### 2.2 Synthesis and testing

#### 2.2.1 Antibacterial activity and hemolysis

We synthesized the selected 14 GN and 14 GP peptides by solid phase peptide synthesis and evaluated the activity of their HPLC-purified trifluoroacetate salts by determining minimum inhibitory concentrations (MIC) against bacteria by broth microdilution assay in Muller–Hinton medium and minimum hemolysis concentrations (MHC) on human red blood cells by serial dilution in phosphate buffer saline (Table 1).

**Table 1:** Synthesis and activity of generated peptides

cpd^a^	Sequence^b^	*P. aeruginosa* c (μg mL^−1^)	*A. baumannii* c (μg mL^−1^)	MRSA^c^ (μg mL^−1^)	MHC^d^ (μg mL^−1^)	*E. coli* c (μg mL^−1^)
**Gram-neg. active, non-hemolytic:**						
**GN1**	**AKRIRKLIKKIFKKI**	***4***	***4***	**16**	***>2000***	**8**
**GN2**	RRWKWRRKIKKWL	*8*	*8*	4	*1000*	16
GN3	IDKWKAAFKKIKNLF	*8–16*	*2*	8	*500*	8–16
GN4	LNALKKVFQKIRQGL	32	*16*	>64	*>2000*	4
GN5	KFFRKLKKLVKK	*16*	>64	64	*>2000*	64
GN6	RLRKKWRKLKKLL	32	*16*–*32*	64	*2000*	16–32

**Gram-neg. active, hemolytic:**						
GN7	KRIRKWVRRILKKL	4	4	4	250	16
GN8	LRKFWKKIRKFLKKI	8	4	4	62.5	16
GN9	KRLWKRIYRLLKK	8	8	8	250	4–8

**Gram-neg. inactive:**						
GN10	IRRIRKKIKKIFKKI	32	32	64	>2000	16
GN11	LRKARRLLKKLRARL	>64	32	32	>2000	32
GN12	GNWRKIVHKIKKAG	32	>64	>64	>2000	16
GN13	AGRLQKVFKVIAK	64	>64	>64	>2000	32
GN14	IHKLAKLAKNVL	>64	>64	>64	>2000	32

**Gram-pos. active, non-hemolytic:**						
**GP1**	**FLKAVKKLIPSLF**	**16**	**8–16**	*16*	*2000*	**8**
GP2	RWRWPILGRILR	8	16	*16*	*500*	16

**Gram-pos. active, hemolytic:**						
GP3	FLHSIGKAIGRLLR	16	16	8	250	8

**Gram-pos. inactive:**						
GP4	GIGAVLNVAKKLL	64	32	32	>2000	16
GP5	KVARFLKKFFR	64	32–64	32	>2000	4
GP6	LKKLWKRIIKVGR	32	16–32	64	>2000	8
GP7	ARKWRKFLKKI	>64	64	64	>2000	32–64
GP8	GRIKRIRKIIHKY	8	32	>64	>2000	32
GP9	ARKKWRKRLKKLKI	32–64	>64	>64	>2000	32–64
GP10	AKKVVKKIYKRFQK	>64	64	>64	>2000	64
GP11	ARKFRRLVKKLR	>64	>64	>64	>2000	64
GP12	LRKARRLVKKLA	>64	>64	>64	>2000	>64
GP13	KRLWKIRQRIAK	>64	>64	>64	>2000	32
GP14	LNALKKVFQKIH	>64	>64	>64	>2000	>64

a Compounds labeled as GN were obtained from the *P. aeruginosa*/*A. baumannii* model, compounds labeled as GP were obtained from the *S. aureus* model; in both sets, compounds were ordered according to their activity and hemolysis profile; **GN2**, **6**, **9**, **10** and **GP2**, **6**, **9**, **11** were obtained using the second selection strategy.

b One-letter code for amino acids. All peptides are carboxamides (–CONH_2_) at the C terminus.

c MIC was determined after incubation for 16–20 h at 37 °C.

d MHC was measured on human red blood cells in 10 mM phosphate buffer saline, pH 7.4, 25 °C. 0.1% Triton X-100 was used as a positive control. Cells in italic denote MIC <32 μg mL^−1^ towards the bacterial strains used for the design (*P. aeruginosa*/*A. baumannii* for GN and *S. aureus* for GP) and MHC ≥500 μg mL^−1^.

Considering an activity threshold of MIC ≤16 μg mL^−1^ for activity and MHC ≥500 μg mL^−1^ for hemolysis, 9 of 14 GN peptides (64%) turned out as actives, but only 6 of 14 GN (43%) were both active against *P. aeruginosa* or *A. baumannii* and non-hemolytic. By the same measure, only 3 of 14 GP peptides (21%) were active against MRSA, and only 2 of 14 GP peptides (14%) were also non-hemolytic. Furthermore, three of the active GN peptides were also active against MRSA, while all three active GP peptides and one GP inactive peptide were also active against *P. aeruginosa* or *A. baumannii*, and 11 out of 14 GN and 6 out of 14 GP peptides showed activity against *Escherichia coli* tested as an additional Gram-negative bacterium. Therefore, in terms of overall activity, 18 out of the 28 synthesized peptides (64%) were active below the threshold, and 14 out of 28 (50%) were active and non-hemolytic, which is not very much below the precision of 76% for the combined activity/hemolysis classifier (see above).

The lack of selectivity of the generated AMPs for the bacteria they were trained on, either Gram-negative (*P. aeruginosa* and *A. baumannii*) or Gram-positive (*S. aureus*) bacteria suggested to test our AMPs in a broader context. We therefore tested the best GN (**GN1**) and the best GP (**GP1**) AMP against additional pathogenic bacteria available in our laboratory (Table 2). Both peptides were also active against ZEM-1A, which is a multidrug-resistant clinical strain of *P. aeruginosa*, but not against the related ZEM9A which is more resistant to polymyxin B, a pattern which we have observed previously with other AMPs.^41,42^**GN2** also showed good activity against *P. aeruginosa* PA14 and several mutant strains generated to be resistant to polymyxin and antimicrobial dendrimers,^43^ and against *S. maltophilia*, *E. cloacae*, both Gram-negative, and to a lesser extent against *S. epidermidis* (Gram-positive), but was inactive against two different strains of *Klebsiella pneumoniae* (Gram-negative). **GP1** also showed significant activity against several of these strains, and even against the two *K. pneumoniae* strains. This extended profiling confirmed the robust activity of both AMPs but also underscored the fact that our generative models did not produce AMPs with selectivity between Gram-negative and Gram-positive strains, reflecting the fact that many AMPs appeared as actives in both TL training sets.

**Table 2:** MIC^a^ of **GN1** and **GP1** towards further MDR and non-MDR bacterial strains

	**GN1**	**GP1**	Polymyxin B
*P. aeruginosa* ZEM-1A^b^^,^^c^	4	4	0.5
*P. aeruginosa* ZEM9A^b^^,^^c^	64	64	4
*P. aeruginosa* PA14^c^	2	8–16	<0.5
*P. aeruginosa* PA14 4.13 (*phoQ*)^c^^,^^d^	2	8–16	1
*P. aeruginosa* PA14 4.18 (*pmrB*)^c^^,^^d^	4	32–64	2
*P. aeruginosa* PA14 2P4 (*pmrB*)^c^^,^^d^	8	64	2
*S. maltophilia* b ^,^ c	4	16	0.5
*E. cloacae* b ^,^ c	8	16–32	1
*K. pneumoniae* (OXA-48)^b^^,^^c^	>64	16–32	1
*K. pneumoniae* NCTC148^b^^,^^c^	>64	32	1
*B. cenocepacia* b ^,^ c	>64	>64	>64
*S. epidermidis* b ^,^ e	16	16	32–64

a The MIC was determined in Müller–Hinton medium after 16–20 h of incubation at 37 °C. Each result represents two independent experiments performed in duplicate.

b MDR strains.

c Gram-negative strains.

d Strains carrying spontaneous mutations in the indicated genes, all leading to polymyxin B resistance.

e Gram-positive strain.

#### 2.2.2 α-Helical folding and membrane disruption

**Fig. 2:**
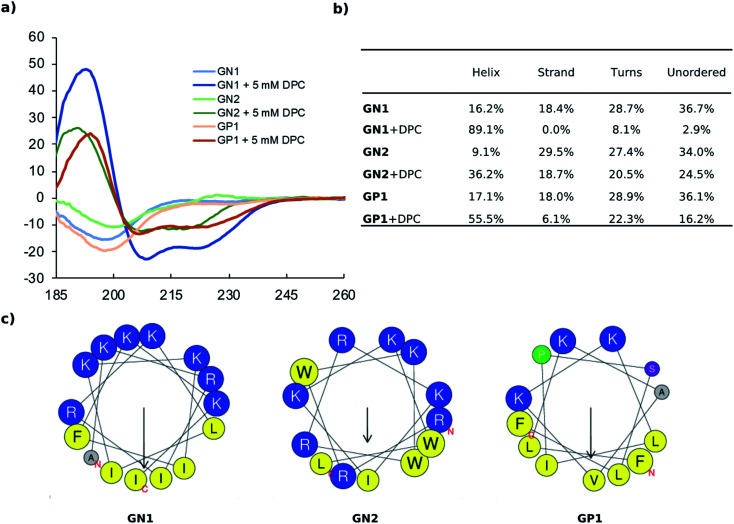
(a) CD spectra of **GN1**, **GN2**, and **GP1** recorded at 0.100 mg mL^−1^ in 10 mM phosphate buffer pH 7.4 with or without 5 mM DPC. (b) Extraction of percentages of secondary structure from primary CD data using DichroWeb. The Contin-LL method and reference set 4 were used. (c) Helix properties predicted by HeliQuest. Circle size proportional to side-chain size, blue indicates cationic residues, yellow indicates hydrophobic residues, grey indicates alanine, green indicates proline, purple indicates serine. The arrows inside each helix wheel indicates the magnitude and direction of the hydrophobic moment.

The amino acid sequences of peptides **GN1** (15 residues, 8 cationic, 7 hydrophobic) and **GP1** (13 residues, 3 cationic, 9 hydrophobic) both had an amphiphilic composition. Circular dichroism (CD) spectra showed that both peptides were unordered in pure water but adopted an α-helical conformation in the presence of *n*-dodecyl phosphocholine (DPC) micelles mimicking the membrane environment. The effect was very strong with **GN1** (89% α-helix with 5 mM DPC) and still quite strong with **GP1** (56% α-helix with 5 mM DPC) despite the presence of a helix-breaking proline residue in its sequence and in line with the fact that this sequence passed the α-helical filter used for sequence selection. By comparison, the second most active, non-hemolytic AMP **GN1** (13 residues, 8 cationic, 5 hydrophobic) which had been selected from the RNN generator and classifiers without the α-helix filter, only showed 36% α-helix with 5 mM DPC. Nevertheless, all three AMPs were predicted to adopt an amphiphilic arrangement of their cationic and hydrophobic side chains upon α-helical folding (Fig. 2c).

To confirm the secondary structure determined by CD, we performed MD (Molecular Dynamics) simulations for our most active peptides **GN1**, **GP1**, and **GN2** using GROMACS.^44^ In each case, 250 ns simulations were performed both in water and in presence of DPC micelle.

**Fig. 3:**
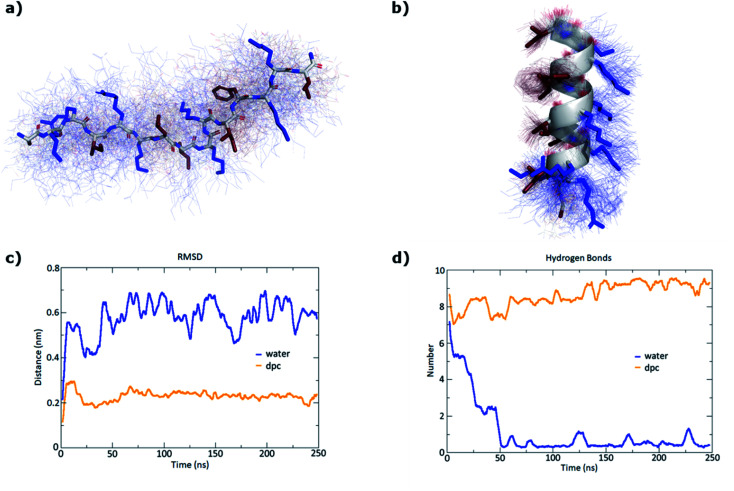
MD simulations of **GN1** in water and in presence of a DPC micelle over 250 ns using GROMACS. (a) Average structure (stick model) in water over 100 structures sampled over the last 100 ns (thin lines). Hydrophobic side chains are colored in red and cationic side chains in blue. (b) Average structure (cartoon model for backbone and stick model for side chains) with DPC micelle over 100 structures sampled over the last 100 ns (thin lines). (c) RMSD (root mean square deviation) of the peptide backbone atoms relative to the starting α-helical conformation. (d) Number of intramolecular hydrogen bonds. The DPC micelle was omitted for clarity.

As expected, simulation in water led to the unfolding of **GN1** (Fig. 3a). Interestingly, **GN1** kept a complete amphiphilic α-helix after 250 ns in presence of DPC micelle (Fig. 3b, c and d), which is consistent with the 89% α-helix obtained with 5 mM DPC during the CD measurements. Similarly, **GP1** and **GN2** unfolded in water and partially folded in presence of DPC micelle (Fig. S4 and S5†). Partial α-helical conformation was observed in the case of **GP1** while interacting with the micelle, confirming the CD data and the conservation of the secondary structure despite the presence of a proline residue. Surprisingly, **GN2** unfolded and refolded into a stable partial π-helix in contact with DPC, suggesting a stable transition state between α-helix and random coil. As both types of helix can not be distinguished using CD, this is coherent with the helicity signal observed with 5 mM DPC. Overall, MD simulations confirmed a helical secondary structure behavior in a membrane-like environment.

**Fig. 4:**
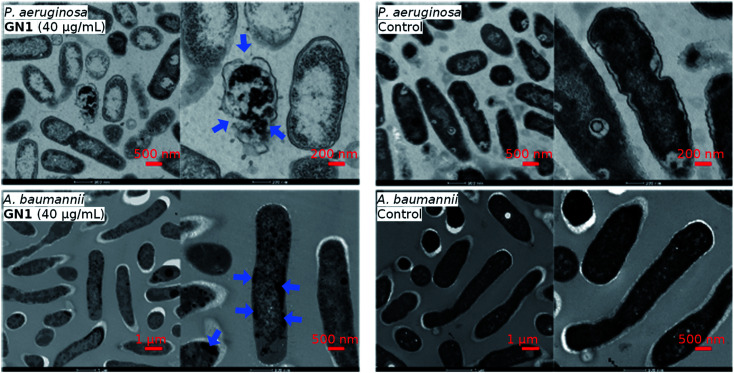
TEM images of *P. aeruginosa* and *A. baumannii*, after 2 hours treatment of **GN1** in MH medium. Blue arrows indicate effects on the bacteria.

The CD, MD, and sequence analysis above clearly pointed to membrane disruption as the probable mechanism of action for our AMPs. This hypothesis was further supported by transmission electron microscopy (TEM) imaging of bacterial cells exposed to the AMP in the case of **GN1**, which showed bacterial membrane ruptures for *P. aeruginosa*, while in the case of *A. baumannii* the cell shape was preserved but cell contents were altered, an effect also observed with other membrane disruptive AMPs on this bacterium (Fig. 4).

## 3. Conclusion

In this work, we have demonstrated ML capable of designing non-hemolytic AMPs. We extracted a highly reliable dataset of AMPs and non-AMPs, as well as hemolytic and non-hemolytic peptides from the DBAASP, a manually curated antimicrobial peptide database. We used the data to train a generative peptide model (prior model), an AMP activity classifier, and a hemolysis classifier. Two copies of the prior model were fine-tuned using active and non-hemolytic peptides against specific strains: *P. aeruginosa*/*A. baumannii* and *S. aureus*, respectively. The fine-tuned models were sampled, and the generated sequences were filtered using the implemented classifiers, basic physicochemical properties, and novelty criteria to obtain short peptides of maximum 15 residues with at least five mutations from the sequences in DBAASP.

Out of the 28 synthesized peptides, 12 were measured active towards the pathogens used in the design (*P. aeruginosa*/*A. baumannii* or *S. aureus*) with a MIC <32 μg mL^−1^, which was the activity threshold selected to train our ML models, and eight of them showed low hemolysis against human blood cells with an MHC ≥500 μg mL^−1^. Additionally, our best compounds **GN1** and **GP1** displayed remarkable activity also against a broader panel of pathogenic bacteria including MDR strains.

In the context of the AMPs previously discovered through a ML-guided approach,^25,28,31,32,35,36^**GN1** and **GP1** have a broader and overall higher activity combined with better hemolytic behavior. Two notable exceptions are the AMPs reported by Nagarajan *et al.*^35^ which have activity and hemolysis comparable to our results, and the two AMPs reported by Cherkasov *et al.*^25^ which show higher activity but a worse hemolytic behavior than our compounds. However, in both cases, hemolysis was not a design feature and the low hemolysis of the reported compounds was serendipitous. Our results indicate that ML can acquire sufficient information from known AMPs to guide the discovery of new AMPs substantially different from the training set and that ML can overcome the challenging task of designing both antimicrobial activity and non-hemolytic behavior. It should be noted that the ML approach exploiting experimental data helped us discover non-hemolytic AMPs even in the absence of a simple design rule for this property, highlighting the usefulness of ML in peptide design.

## 4. Methods

### 4.1 Datasets preparation

All peptide sequences without intrachain bonds were downloaded from the DBAASP peptide database website (https://dbaasp.org/), resulting in a dataset of 11 805 linear peptides. Only the 9946 sequences with free or amidated C-terminus, free or acetylated N-terminus, and containing only natural amino acids and their d-enantiomers were kept.

The targets and the activity measurements of the 9946 sequences were extracted using the DBAASP Python API. Sequences with a registered activity measure below 10 μM, or 10 000 nM, or 32 μg mL^−1^ towards at least one reported target were labeled as active; the sequences active against *P. aeruginosa*, *A. baumannii*, or *S. aureus* were flagged. Sequences with registered activity measures above 10 μM, or 10 000 nM, or 32 μg mL^−1^ towards all reported targets were labeled as inactive; when *P. aeruginosa*, *A. baumannii*, or *S. aureus* was one of the reported targets the sequences were flagged. When present, activity against human erythrocytes was used to label the sequences as hemolytic or non-hemolytic. The concentration was normalized to μM and sequences causing less than 20% of hemolysis with a concentration equal or above 50 μM were flagged as non-hemolytic. Sequences causing more than 20% of hemolysis were flagged as hemolytic regardless of the concentration. The remaining sequences, together with the ones not having reported data against human erythrocytes, were labeled as of unknown hemolytic properties. The procedure resulted in 4774 peptides labeled as active, 1867 labeled as inactive, 1319 labeled as hemolytic, and 943 labeled as non-hemolytic.

To achieve a balanced dataset for the activity classifiers, 2907 additional inactive sequences were generated. (1) 1453 unique sequences with the same length distribution of a randomly selected subset of the active sequences were obtained fragmenting an equally sized set of sequences randomly selected from Swissprot. (2) 1454 unique sequences were obtained scrambling a randomly selected subset of the active sequences. The 9548 obtained active and inactive unique peptide sequences were divided in training and test with a 75–25 random split. In the evaluation process, the active sequences were considered as the positive class and the inactive sequences as the negative class. For the hemolysis classifier, we used the same training test split but selecting only the sequences with hemolysis data. In the evaluation, we considered the non-hemolytic sequences as the positive class and the hemolytic sequences as the negative class.

### 4.2 NB, SVM, and RF classifiers

The NB, non-linear SVM, and RF classifiers were implemented using scikit-learn.^45^ The sequences were padded to the maximum sequence length (190 residues) and tokenized as singular amino acids (or empty position), then each token was mapped to a unique number. The SVM and the RF models were optimized with a grid search to increase the ROC AUC of the test set (Table 1).

### 4.3 RNN classifiers

The AMP activity RNN classifier and the hemolysis RNN were implemented in PyTorch.^46^ The input of the implemented RNN classifiers are the tokenized and “one-hot” encoded sequences. The sequences were tokenized as singular amino acids and a start and an end tokens were added; then each token was mapped to a unique number. The resulting vector was transformed into a matrix where the number of columns is the length of the vocabulary and the number of rows was the length of the vector itself. The presence of a specific residue at each position was represented with a 1 while the rest of the matrix is filled with zeros.

The models were composed of an embedding layer, gated recurrent unit (GRU)^47^ cells, and a linear transformation layer followed by a softmax function.^48^ The output of the model was considered only when the last token was reached (Fig. S2†). The hyperparameters of the RNN classifiers were optimized to maximize the ROC AUC of the test set (Table S1†). A threshold was picked to keep the prediction of false positives below 6%. The parameters were learned using a negative log-likelihood loss^48^ and a stochastic gradient descent^49^ with a momentum of 0.9 and a learning rate of 0.01.

To create a baseline prediction for both RNN classifiers, a second RNN AMP activity and hemolysis classifiers (RNN AMP activity classifier scrambled labels and RNN hemolysis classifier scrambled labels) were implemented (Table S2†) and trained using a different dataset, where the sequences were the same, but the activity and the hemolytic labels were randomly scrambled.

### 4.4 RNN generative models

A generative model was implemented in PyTorch with the same architecture of the previously described RNN activity classifier, with the exception of the dimensionality of the last linear layer which is the same size of the vocabulary (41 tokens, 41 dimensions, Fig. S3†). Furthermore, in this case, the output of the model was considered at every token, allowing the sequence generation. The input sequences were processed as for the RNN classifiers. The parameters of the RNN generative model were learned using negative log-likelihood loss (NLLL) and Stochastic gradient descent with a momentum of 0.9 and a learning rate of 0.001. During the training of the generator, only the active sequences of the training set were used, but the NLLL on the test set was also monitored. The training was stopped when the NLLL of the test reached its minimum.

### 4.5 Transfer learning

The 242 active sequences of the training set flagged against *P. aeruginosa* or *A. baumannii* and annotated as non-hemolytic were used to train again the generative model and fine-tune it against Gram-negative bacteria. The 312 active sequences of the training set flagged against *S. aureus* and annotated as non-hemolytic were used to train again the generative model and fine-tune it against Gram-positive bacteria. The parameters were learned using negative log-likelihood loss (NLLL) and Stochastic gradient descent with a momentum of 0.9 and a learning rate of 0.00001. As for the training of the prior model, the NLLL on the flagged subset of the test set, consisting of 97 for the *P. aeruginosa* or *A. baumannii* and of 137 sequences for the *S. aureus* model, was monitored and when it reached its minimum the training was stopped.

### 4.6 Sampling and properties calculation

50 000 sequences were sampled from each of the two transfer learned models. The Levenshtein distance (LD) from the nearest neighbor (NN) in the training and the test of both RNN classifiers was calculated using the Levenshtein Python package.^50,51^ The helicity prediction was performed using SPIDER3,^52^ and the helicity fraction was calculated as the number of residues predicted helical in a peptide sequence divided by the length of the sequence itself. The hydrophobic moment was calculated as described by Eisenberg *et al.*^40^ Hemolysis and activity were predicted by the respective classifiers converting the probabilistic prediction values into binary classification using the threshold that kept the prediction of false positive below 6% (0.99205756 for the activity classifier and 0.99981695 for the hemolysis classifier).

### 4.7 Sequences selection

The generated sequences were filtered based on multiple criteria. First, to ensure novelty, we have chosen sequences with LD >5 from the hemolysis classifier training set sequences and LD >4 from the hemolysis classifier test set sequences. Second, we remove all sequences that were outside the applicability domain of the hemolysis classifier. To do so, we calculated the minimum LD of every test set compound to the training set. Giving this minimum LD values we defined to applicability domain of the classifier to be the 90% quantile. This led to the exclusion of all generated sequences with a LD distance of 8 or more to the training set of the hemolysis classifier. Only sequences up to 15 residues were selected to facilitate the synthesis process and due to the low percentage of d amino acids in the training set, sequences containing d-residues were excluded. The sequences were further selected following two different strategies.

#### 4.7.1 First selection strategy

Since helicity and amphiphilicity often correlate with antimicrobial activity, we selected sequences with a predicted helicity fraction above 0.8 and an Eisenberg hydrophobic moment above 0.3. The thresholds for the predicted helicity fraction and hydrophobic moment were chosen based on the median values of the active sequences in the training and test, respectively 0.83 and 0.31. The filtered sequences were clustered using the RDKit^53^ Butina module with a threshold of 10 and the Levenshtein distance as distance function. Sequences containing methionine and sequences with an LD >5 from the training and test sets of the activity classifier were excluded from all clusters. The center of each cluster was picked, and in addition, one additional compound was selected at random from the clusters containing more than 6 compounds. The workflow resulted in 10 sequences predicted active against Gram-negative bacteria and 16 sequences predicted active against Gram-positive bacteria (ESI file 1†). 10 sequences for each class were selected for synthesis.

#### 4.7.2. Second selection strategy

To avoid the bias that secondary structure evaluation and the clustering might create and to gain a better insight on the activity of the sequences generated by the two transfer learned models, we randomly sampled 20 sequences (10 for each class, ESI file 1†). Four sequences predicted active against Gram-positive and five against Gram-negative were manually selected. Non-containing methionine sequences with higher distances from the training and test sets of the activity classifier were preferred.

### 4.8. Evaluation metrics

ROC AUC is the area under the ROC curve, and the ROC curve is obtained by plotting the true positive rate (TPR) against the false positive rate (FPR):
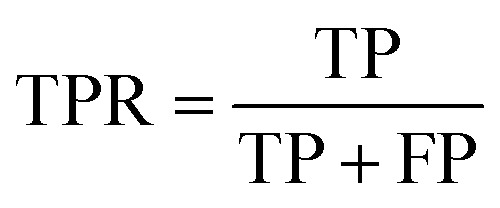

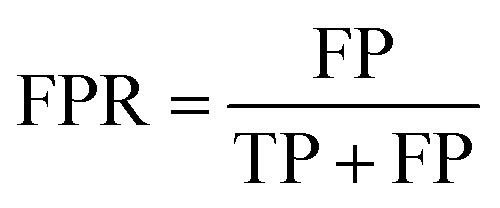
where TP stands for true positives, TN for true negatives, FP for false positives, and FN for false negatives predicted by the classifier.

The F1 score is defined as the harmonic mean of precision and recall:Precision = TPR
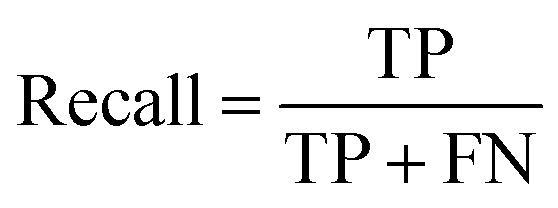

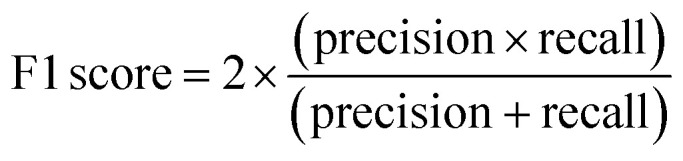


The balanced accuracy is defined as:
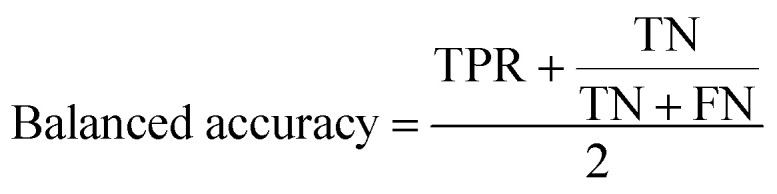


The Matthews correlation coefficient (MCC) is a correlation between the observed and the predicted class and it is defined as:
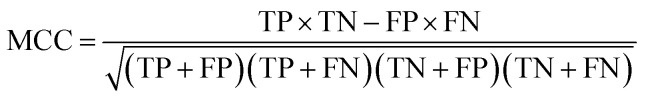


### 4.9 Peptide synthesis

Peptides were synthesized using standard 9-fluorenylmethoxycarbonyl (Fmoc) Solid Phase Peptide Synthesis. All syntheses were performed at 60 °C under nitrogen bubbling. 400 mg Rink Amide AM resin LL (0.26 mmol g^−1^) were used for each peptide. The resin was firstly deprotected twice one minute and four minutes using a deprotection cocktail containing 5% w/v piperazine, 2% v/v 1,8-diazabicyclo(5.4.0)undec-7-ene (DBU) and 10% v/v 2-butanol in *N*,*N*-dimethylformamide (DMF). For each amino acid, a doubling coupling was performed (twice eight minutes) using for each coupling 3 mL of 0.2 M of the corresponding Fmoc protected amino acid in DMF, 1.5 mL of 0.5 M Oxyma in DMF, and 2 mL of 0.5 M *N*,*N*′-diisopropylcarbodiimide (DIC) in DMF. Deprotection steps (double deprotection, one minute, and four minutes) were achieved using the same cocktail described above, except for sequences containing aspartic acid for which a solution of 20% v/v piperidine + 0.7% v/v formic acid in DMF was used to avoid aspartimide and side products formation.

After the last deprotection, peptides were cleaved from the resin using 7 mL of a mixture trifluoroacetic acid/triisopropylsilane/mQ water (TFA/TIS/H_2_O) with the corresponding ratios 94/5/1 during three hours. Peptides were then precipitated using approximatively 25 mL of cold terbutylmethyl ether and centrifuged 10 minutes at 4400 rpm. Supernatant was removed and peptides were washed twice with 15 mL of cold terbutylmethyl ether before lyophilization.

### 4.10 Minimal inhibitory concentration

To determine the minimal inhibitory concentration (MIC), the broth microdilution method was used. A colony of bacteria was grown in LB (Lysogeny broth) medium overnight at 37 °C. The samples were prepared as stock solutions of 8 mg mL^−1^ in H_2_O, diluted to the initial concentration of 64 or 128 μg mL^−1^ in 300 μL Mueller–Hinton (MH) medium, added to the first well of 96-well microtiter plate (TPP, untreated), and diluted serially by 1/2. The concentration of the bacteria was quantified by measuring absorbance at 600 nm and diluted to OD_600_ = 0.022 in MH medium. The sample solutions (150 μL) were mixed with 4 μL diluted bacterial suspension with a final inoculation of about of 5 × 10^5^ CFU. The plates were incubated at 37 °C until satisfactory growth (∼18 h). For each test, two columns of the plate were kept for sterility control (broth only) and growth control (broth with bacterial inoculums, no antibiotics). The MIC was defined as the lowest concentration of the peptide dendrimer that inhibited visible growth of the tested bacteria, as detected after treatment with MTT.

### 4.11 Hemolysis assay

Compounds were subjected to a hemolysis assay to assess the hemolytic effect on human red blood cells (hRBCs). The blood was obtained from Interregionale Blutspende SRK AG, Bern, Switzerland. 1.5 mL of whole blood was centrifuged at 3000 rpm for 15 minutes at 4 °C. The plasma was discarded, and the hRBC pellet was re-suspended in 5 mL of PBS (pH 7.4) then centrifuged at 3000 rpm for 5 minutes at 4 °C. The washing of hRBC was repeated three times and the remaining pellet was re-suspended in 10 mL of PBS.

The samples were prepared as the initial concentration of 4000 μg mL^−1^ in PBS, added to the first well of 96-well microtiter plate (TPP, untreated) and diluted serially by 1/2. After diluted, 100 μL of sample was in each well and the final sample concentration was 4000 μg mL^−1^, 2000 μg mL^−1^, 1000 μg mL^−1^, 500 μg mL^−1^, 250 μg mL^−1^, 125 μg mL^−1^, 62.5 μg mL^−1^ and 31.3 μg mL^−1^. Controls on each plate included a blank medium control (PBS 100 μL) and a hemolytic activity control (0.1% Triton X-100). 100 μL of hRBC suspension was incubated with 100 μL of each sample in PBS in 96-well plate (Nunc 96-Well Polystyrene Conical Bottom MicroWell Plates). After the plates were incubated for 4 h at room temperature, minimal hemolytic concentration (MHC) was determined by visual inspection of the wells. 100 μL supernatants was carefully pipetted to a flat bottom, clear wells plate (TPP® tissue culture plates, polystyrene).

### 4.12 Circular dichroism spectroscopy

CD spectra were recorded using a Jasco J-715 spectrometer equipped with a PFD-350S temperature controller and a PS-150J power supply. All experiments were measured using a Hellma Suprasil R 100QS 0.1 cm cuvette. Stock solution (1.00 mg mL^−1^) of dendrimers were freshly prepared in 10 mM phosphate buffer (pH 7.4). For the measurement, the peptides were diluted to 100 μg mL^−1^ with buffer and 5 mM Dodecylphosphocholine (DPC, Avanti Polar Lipids, Inc., USA) was added when specified. The range of measurement was 185–260 nm, scan rate was 20 nm min^−1^, pitch 0.5 nm, response 16 s and band 1.0 nm. The nitrogen flow was kept above 10 L min^−1^. The blank was recorded under the same conditions and subtracted manually. Each sample was subjected to two accumulations. The cuvettes were washed with 1 M HCl, mQ-H_2_O and buffer before each measurement. Percentage of different secondary structure was calculated by DichroWeb.

### 4.13 Transmission electron microscopy

Exponential phase of *Pseudomonas aeruginosa* PAO1 and *A. baumannii* were washed with MH medium and treated with **GN1** at the concentration of 10 × MIC. After 2 h incubation, 1 mL of the bacteria (OD_600_ = 1) were centrifuged at 12 000 rpm for 3 min and fixed overnight with 2.5% glutaraldehyde (Agar Scientific, Stansted, Essex, UK) in 0.15 M HEPES (Fluka, Buchs, Switzerland) with an osmolarity of 670 mOsm and adjusted to a pH of 7.35. The next day, PAO1 were washed with 0.15 M HEPES three times for 5 min, postfixed with 1% OsO4 (SPI Supplies, West Chester, USA) in 0.1 M Na-cacodylate-buffer (Merck, Darmstadt, Germany) at 4 °C for 1 h. Thereafter, bacteria cells were washed in 0.1 M Na-cacodylate-buffer three times for 5 min and dehydrated in 70, 80, and 96% ethanol (Alcosuisse, Switzerland) for 15 min each at room temperature. Subsequently, they were immersed in 100% ethanol (Merck, Darmstadt, Germany) three times for 10 min, in acetone (Merck, Darmstadt, Germany) two times for 10 min, and finally in acetone–Epon (1 : 1) overnight at room temperature. The next day, bacteria cells were embedded in Epon (Fluka, Buchs, Switzerland) and hardened at 60 °C for 5 days.

Sections were produced with an ultramicrotome UC6 (Leica Microsystems, Vienna, Austria), first semithin sections (1um) for light microscopy which were stained with a solution of 0.5% toluidine blue O (Merck, Darmstadt, Germany) and then ultrathin sections (70–80 nm) for electron microscopy. The sections, mounted on single-slot copper grids, were stained with 1% uranyl acetate at 40 °C for 30 min and 3% lead citrate at RT for 20 min or UranyLess (Electron Microscopy Sciences, Hatfield, UK) at 40 °C for 10 min and 3% lead citrate at 25 °C for 10 min with an ultrostainer (Leica Microsystems, Vienna, Austria). Sections were then examined with a Tecnai Spirit transmission electron microscope equipped with two digital cameras (Olympus-SIS Veleta CCD Camera, FEI Eagle CCD Camera).

### 4.14 Molecular dynamics

Molecular dynamics (MD) simulations were performed for the peptides **GN1**, **GP1** and **GN2** using GROMACS software version 2018.1 and the gromos53a6 force field.^44,54^ The starting topologies were obtained from the α-helical secondary structures built in PyMol Molecular Graphics System. A dodecahedral box was created around the peptide 1.0 nm from the edge of the peptide and filled with extended simple point charge water molecules. Sodium and chloride ions were added to produce an electroneutral solution at a final concentration of 0.15 M NaCl. The energy was minimized using a steepest gradient method to remove any close contacts before the system was subjected to a two-phase position-restrained MD equilibration procedure. The system was first allowed to evolve for 100 ps in a canonical NVT (N is the number of particles, V the system volume, and T the temperature) ensemble at 300 K before pressure coupling was switched on and the system was equilibrated for an additional 100 ps in the NPT (P is the system pressure) ensemble at 1.0 bar.

#### 4.14.1 MD in presence of DPC micelle

MD simulations in the presence of a DPC (*n*-dodecylphosphocholine) micelle were performed as follows. Parameters and references for the DPC molecule^55^ for the GROMOS53a6 forcefield are given in the ESI (Note 4†). Peptides were manually placed at a distance from the pre-equilibrated micelle (of 65 DPC molecules) equal to the diameter of said peptide. Box, solvation and NVT equilibration procedures were performed as explained previously. For each peptide/micelle system, 10 runs of 50 ns were generated to show the possibility for the peptide to either interact or diffuse away from the micelle. Then, runs of interest were extended up to 250 ns.

#### 4.14.3 Clustering of stable structures

To obtain a representative conformer for each MD run, the last 100 ns (10 001 frames) of each run were clustered using an RMSD cut-off adapted to get a good balance between the number of clusters and the size of the main cluster. A large number of clusters combined with a very large percentage of structures in the top cluster is an indication of the stability of the one main conformer in each case. The PyMol Molecular Graphics System, version 1.8 (Schrödinger, LLC), was used to create structural models.

## Data availability

The source code and dataset used for this study are available at https://github.com/reymond-group/MLpeptide.

## Author contributions

AC designed the study, performed the machine learning studies, and wrote the paper. XC co-designed the study, performed peptides synthesis and tests, and wrote the paper. HP performed peptide synthesis, molecular dynamics simulations, and wrote the paper. JLR co-designed and supervised the study and wrote the paper. All authors read and approved the final manuscript.

## Conflicts of interest

There are no conflicts to declare.

## Supplementary Material

SC-012-D1SC01713F-s001
