# Supplementary material for: Machine learning designs non-hemolytic antimicrobial peptides
Source: Chem Sci. 2021 Jun 7;12(26):9221–32. doi: 10.1039/d1sc01713f (PMC8285431; doi:10.1039/d1sc01713f)
Supplement: SC-012-D1SC01713F-s001 [file SC-012-D1SC01713F-s001.pdf]

Electronic Supplementary Material (ESI) for Chemical Science.
This journal is © The Royal Society of Chemistry 2021

Supporting Information for:

Machine Learning Designs Non-Hemolytic Antimicrobial Peptides

Alice Capecchi,[a) ¥] Xingguang Cai,[a) ¥] Hippolyte Personne,[a)] Thilo Köhler,[b, c)] Christian van Delden[b,c)] and Jean-Louis Reymond[a)]*

[a)] *Department of Chemistry, Biochemistry and Pharmaceutical Sciences, University of Bern, Freiestrasse 3, 3012 Bern, Switzerland;* [b)] *Department of Microbiology and Molecular Medicine, University of Geneva;* [c)] *Service of Infectious Diseases, University Hospital of Geneva, Geneva, Switzerland*

¥) *These two authors contributed equally.*

*E-mail: jean-louis.reymond@dcb.unibe.ch

Supporting information Note 1: Tables

Table S1. Performance on the test of the NB, RF, SVM, RNN, and RNN with scrambled labels (RNN scr.) models for the AMP activity (act.) and hemolysis (hem.) classification tasks.

**Classifier**	**ROC AUC**	**Accuracy** [a)]	**Precision** [a)]	**Recall** [a)]	**F1 score** [a)]	**MCC** [a)]
NB act.	0.55	0.55	0.59	0.32	0.42	0.11
SVM act.	0.75	0.68	0.68	0.68	0.68	0.36
RF act.	0.81	0.71	0.70	0.75	0.73	0.44
**RNN act.**	**0.84**	**0.76**	**0.74**	**0.80**	**0.77**	**0.53**
RNN scr. act.	0.51	0.49	0.35	0.03	0.05	-0.06
NB hem.	0.58	0.56	0.48	**0.76**	0.59	0.19
SVM hem.	0.69	0.73	0.72	0.58	0.65	0.44
RF hem.	0.80	**0.77**	**0.81**	0.60	0.69	**0.53**
**RNN hem.**	**0.87**	**0.76**	0.70	**0.76**	**0.73**	**0.52**
RNN scr. hem.	0.45	0.61	0.41	0.05	0.10	0.01

a) The probabilistic prediction values were converted into binary classification values using a threshold of 0.5. The best values and the selected classifiers are reported in bold.

Table S2. Classifiers optimization

**Classifier**	**Hyperparameters optimization** [a)]
SMV AMP activity	C = 0.1, 1, **10**, 100
	γ = 0.1, 0.01, **0.001**
RF AMP activity	maximum depth = **10**, 30, 50, 70, 90, None
	no. estimators = 10, 100, 250, 500, 750, **1000**, 1500, 2000
RNN AMP activity	embedding dimensions = 2, 21, 42, **100**
	GRU dimensions = 50, 100, 200, 300, **400**
	no. layers = 1, **2**, 3
	epoch = [1, 2, 3, ..., 150]; best epoch = **38**
RNN AMP activity	embedding dimensions **2**, 21, 42, 100
scrambled labels	GRU dimensions = **50**, 100, 200, 300, 400
	no. layers = **1**, 2, 3
	epoch = [1, 2, 3, ..., 150]; best epoch = **1**
SMV Hemolysis	C = 0.1, **1**, 10, 100
	γ = 0.1, 0.01, **0.001**
RF Hemolysis	maximum depth = **10**, 30, 50, 70, 90, None
	no. estimators = 10, 100, 250, **500**, 750, 1000, 1500, 2000
RNN Hemolysis	embedding dimensions = 2, 21, 42, **100**
	GRU dimensions = 50, 100, 200, 300, **400**

	no. layers = **1**, 2, 3
	epoch = [1, 2, 3, ..., 150]; best epoch = **95**
RNN Hemolysis scrambled labels	embedding dimensions **2**, 21, 42, 100
	GRU dimensions = **50**, 100, 200, 300, 400
	no. layers = 1, **2**, 3
	epoch = [1, 2, 3, ..., 150]; best epoch = **150**

a) The selected hyperparameters are highlighted in bold. All hyperparameters that have not been discussed have been used in their default values.

Supporting information Note 2: Figures

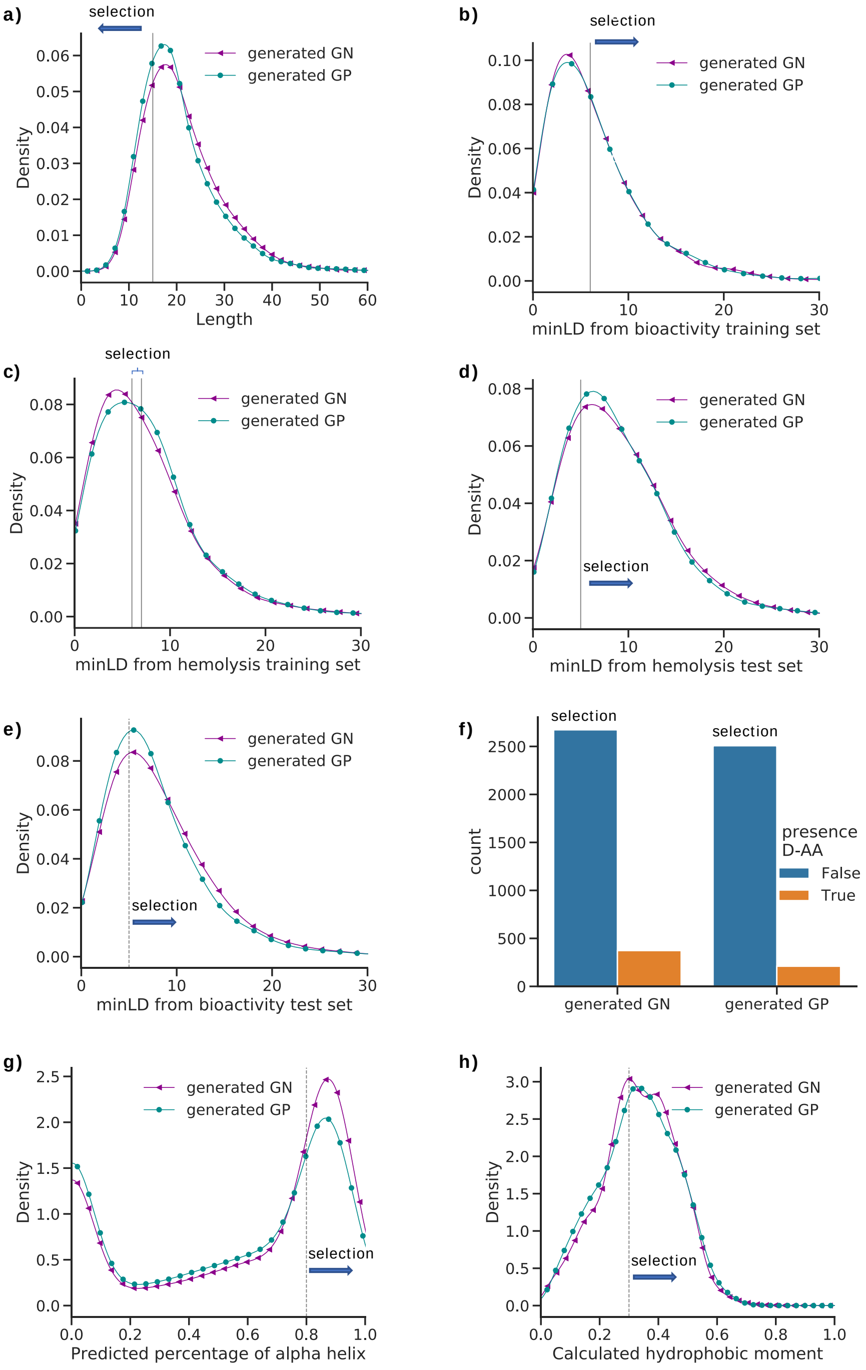

Figure S1. Properties distribution and filters. (a) length, (b-e) minimum Levenshtein distance (minLD) from training and test sets, (f) presence of D-amino acids (D-AA), (g, h) Amphiphilic helix estimation, of the 3,046 predicted active and non-hemolytic sequences derived from the model fined tuned for *A. baumannii* and *P. aeruginosa* (Generated GN) and the 2,717 predicted active and non-hemolytic sequences derived from the model finetuned for *S. aureus*. (a-d) Solid vertical lines indicate that the threshold values were included. (e, g, h) Dashed vertical lines indicated that the threshold lines were excluded.

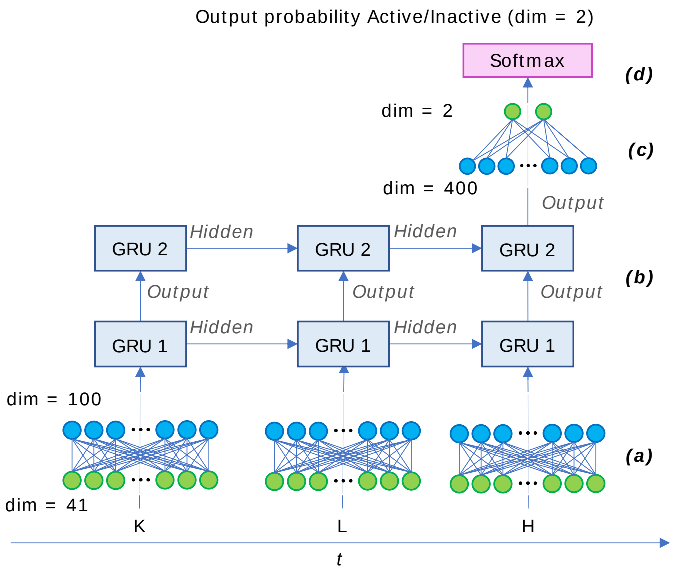

Figure S2. RNN AMP activity classifier architecture. The tokenized and "one-hot" encoded sequences enter an 100 dimensions (dim) embedding layer (a), then they are processed through two layers of 400 dimensions GRU cells (b), and finally, a linear transformation layer shapes the last GRU output into two dimensions (c), followed by a softmax function that normalizes it into a probability (d). The architecture of the hemolytic classifier differs only by having one layer of GRU cells.

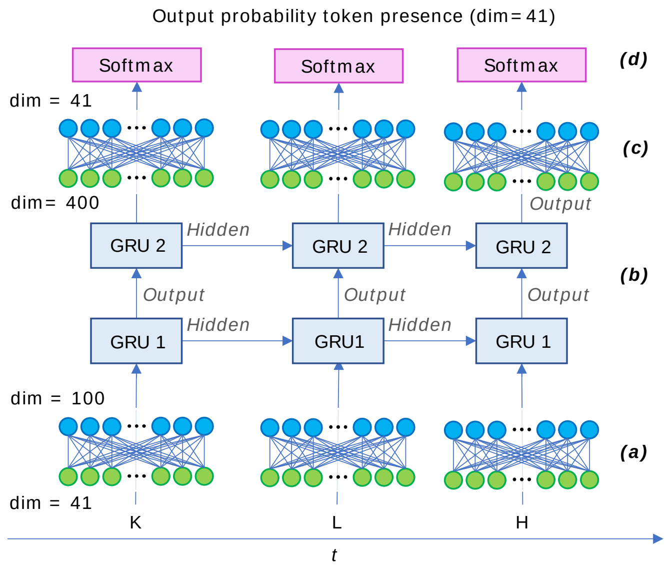

Figure S3. RNN generative models architecture. The tokenized and "one-hot" encoded sequences enter an 100 dimensions (dim) embedding layer (a), then they are processed through two layers of 400 dimensions GRU cells (b), and finally, a linear transformation layer shapes the last GRU output into 41 dimensions (c), followed by a softmax function that normalizes it into a probability (d). The architecture of the hemolytic classifier differs only by having one layer of GRU cells.

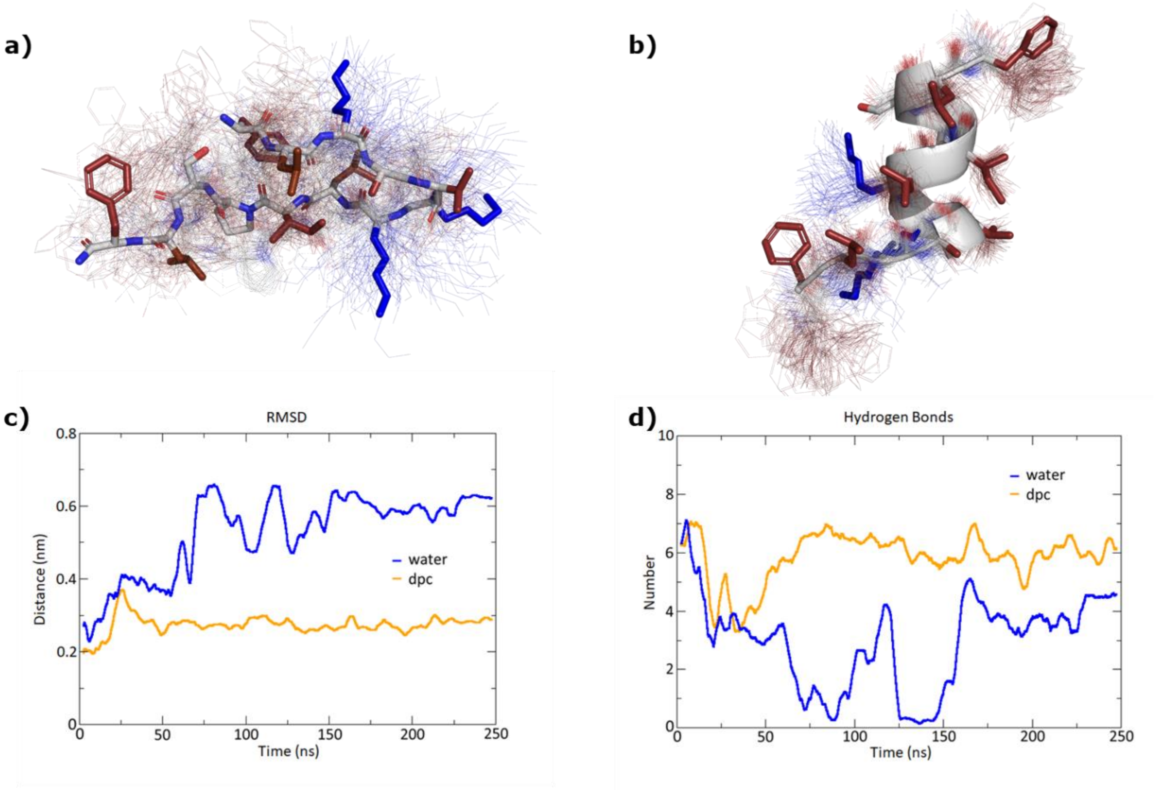

Figure S4: MD simulations of **GP1** in water and in presence of a DPC micelle over 250 ns using GROMACS. (**a**) Average structure (stick model) in water over 100 structures sampled over the last 100 ns (thin lines). Hydrophobic side chains are colored in red and cationic side chains in blue. (**b**) Average structure (cartoon model for backbone and stick model for side chains) with DPC micelle over 100 structures sampled over the last 100 ns (thin lines). (**c**) RMSD (root mean square deviation) of the peptide backbone atoms relative to the starting α-helical conformation. (**d**) Number of intramolecular hydrogen bonds. The DPC micelle was omitted for clarity.

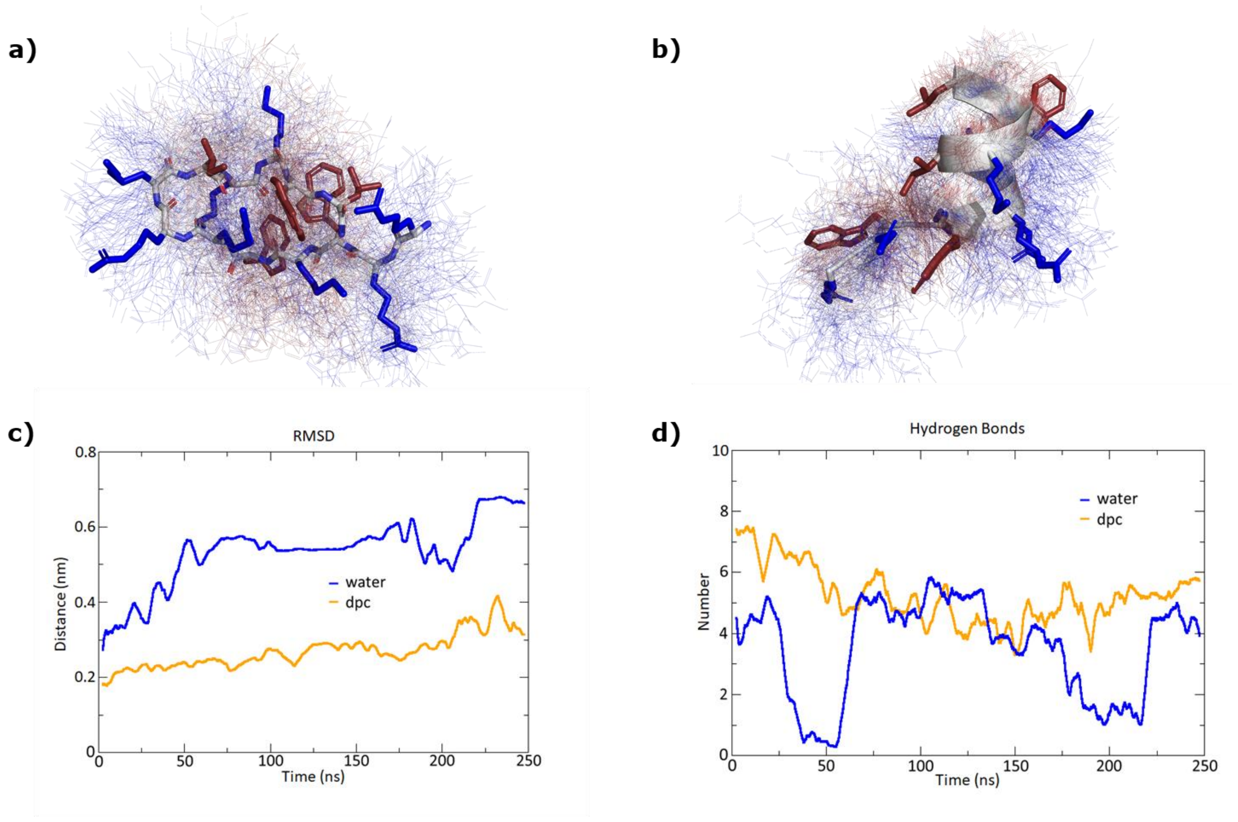

Figure S5: MD simulations of **GN2** in water and in presence of a DPC micelle over 250 ns using GROMACS. (**a**) Average structure (stick model) in water over 100 structures sampled over the last 100 ns (thin lines). Hydrophobic side chains are colored in red and cationic side chains in blue. (**b**) Average structure (cartoon model for backbone and stick model for side chains) with DPC micelle over 100 structures sampled over the last 100 ns (thin lines). (**c**) RMSD (root mean square deviation) of the peptide backbone atoms relative to the starting α-helical conformation. (**d**) Number of intramolecular hydrogen bonds. The DPC micelle was omitted for clarity.

Supporting information Note 3: HPLC/MS and HRMS spectra

GN1 (AKRIRKLIKKIFKKI-NH_2) was obtained from Rink Amide AM resin LL (400 mg, 0.26 mmol/g), the peptide was obtained as a white foamy solid after preparative RP-HPLC purification (302.5 mg, 9.6%). Analytical RP-HPLC: t_R = 1.35 min (100% A to 100% D in 3.5 min, λ= 214 nm). MS (ESI+): $C_{90}H_{168}N_{28}O_{15}$ calc./obs. 1881.32/1881.33 $[M]^+$.

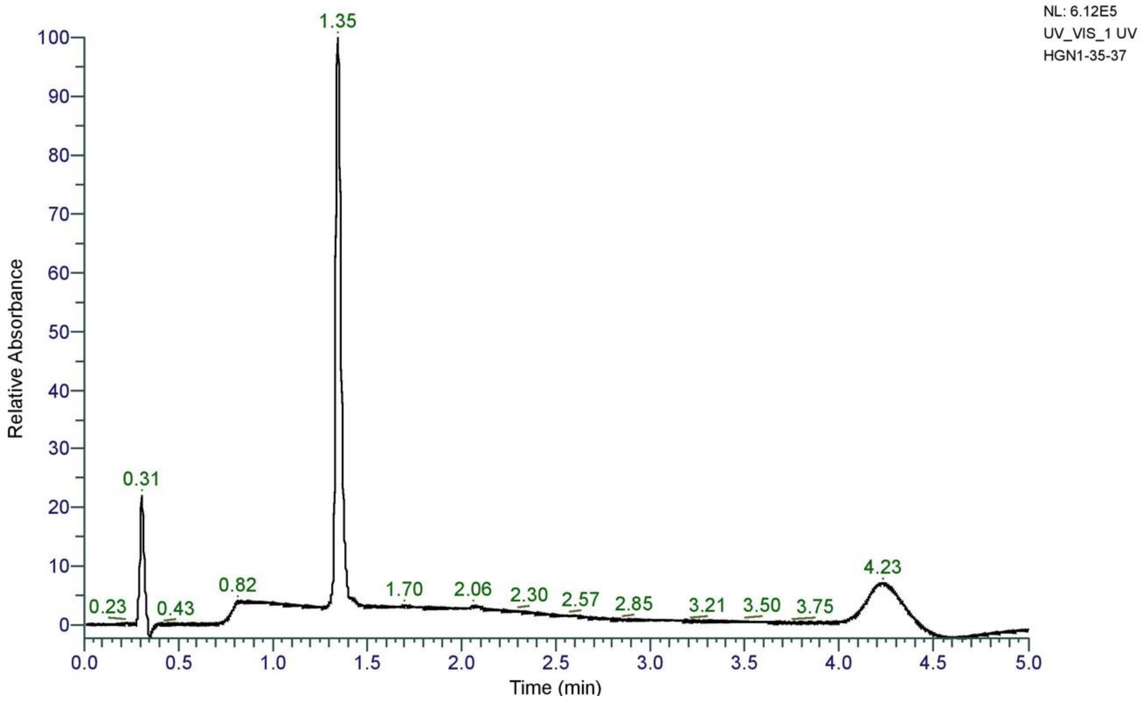

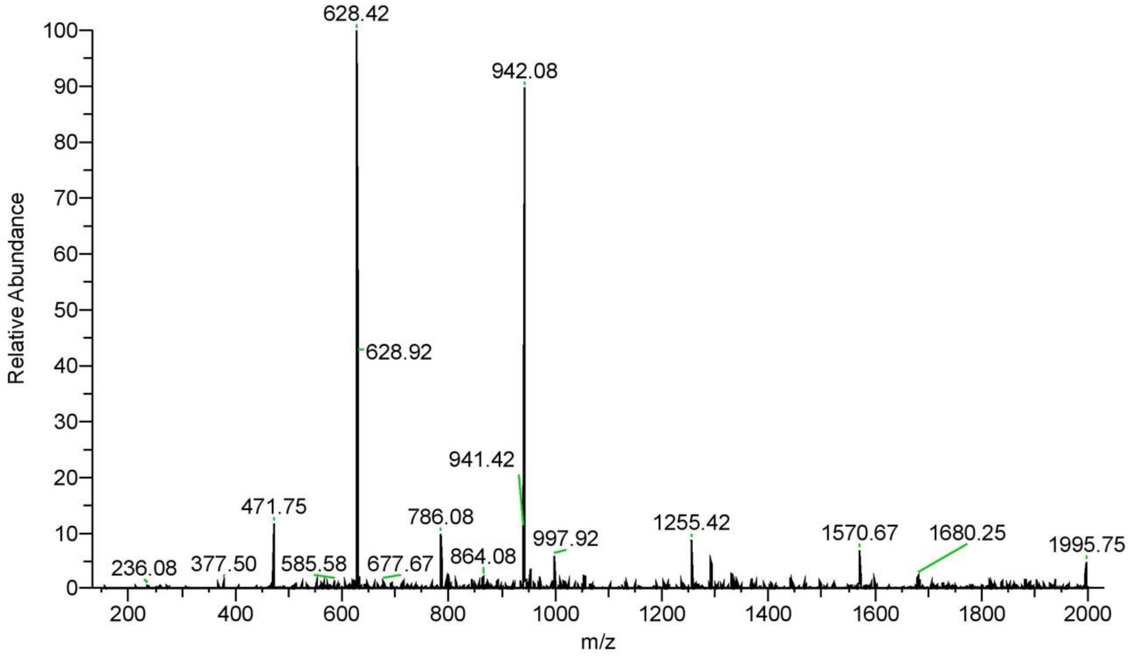

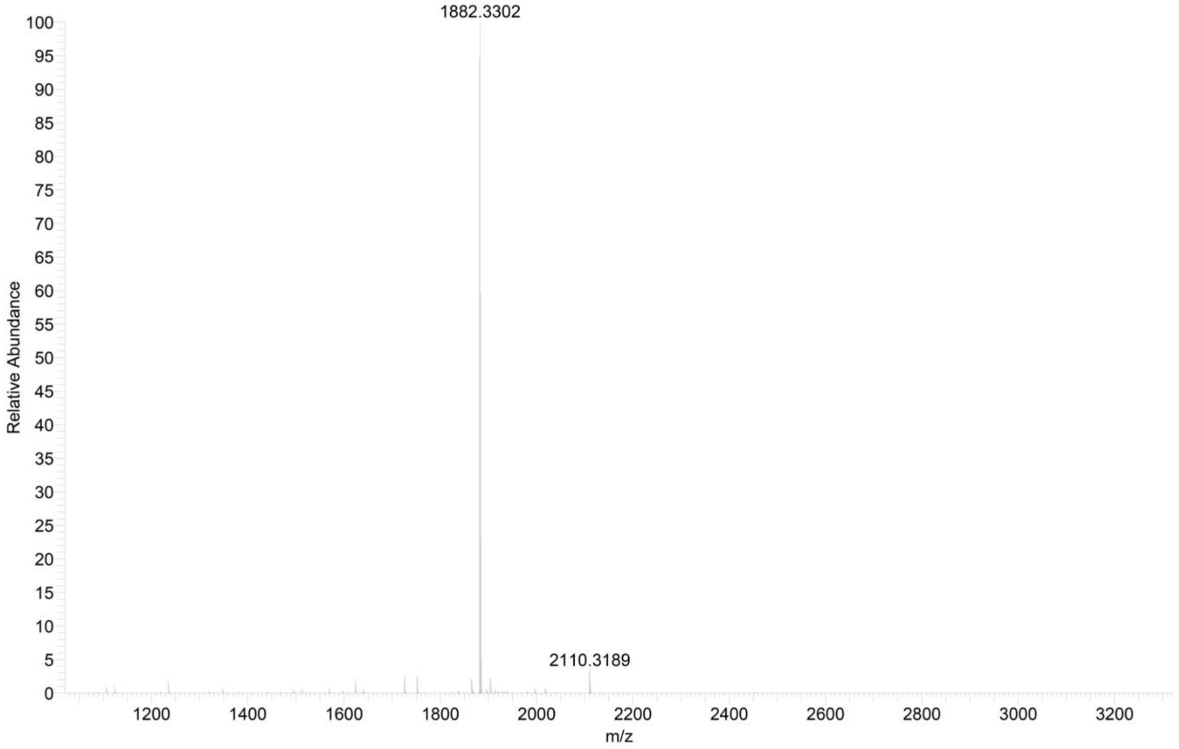
1882.3302
2110.3189
Relative Abundance
m/z

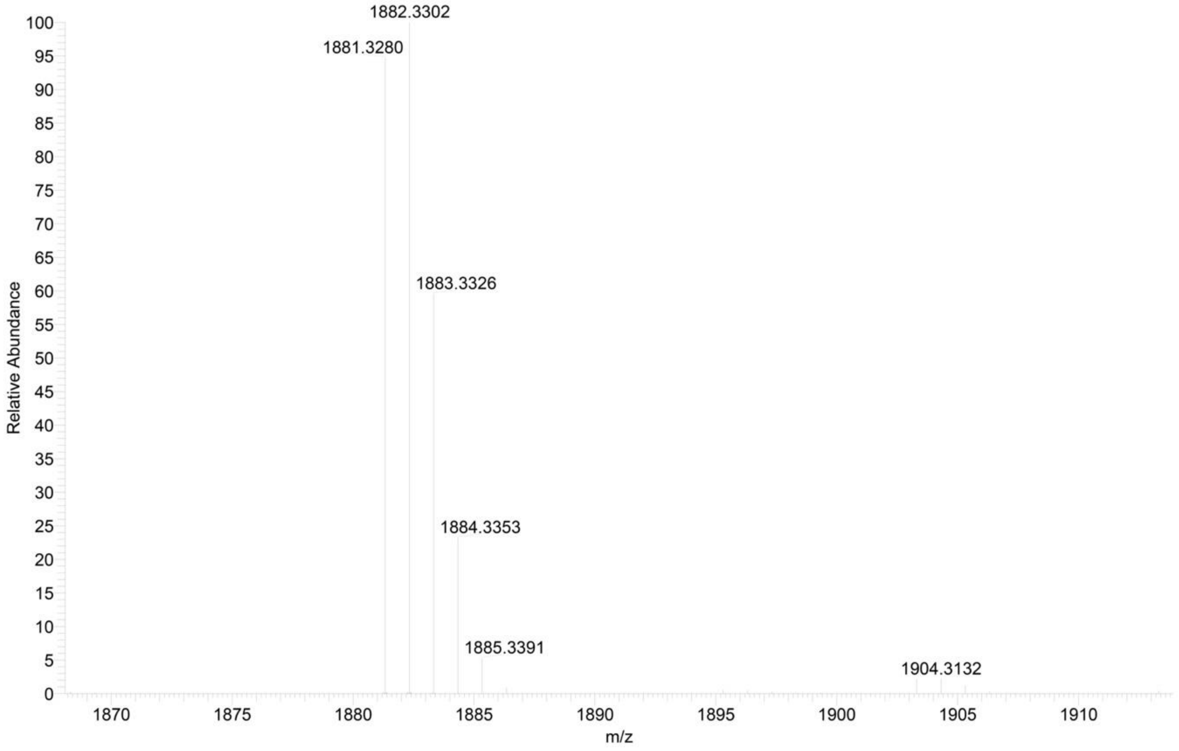
1882.3302
1881.3280
1883.3326
1884.3353
1885.3391
1904.3132
Relative Abundance
m/z

GN2 (RRWKWRRKIKKWL-NH_2) was obtained from Rink Amide AM resin LL (400 mg, 0.26 mmol/g), the peptide was obtained as a white foamy solid after preparative RP-HPLC purification (308.4 mg, 39.0%). Analytical RP-HPLC: t_R = 1.29 min (100% A to 100% D in 3.5 min, λ= 214 nm). MS (ESI+): $C_{93}H_{151}N_{33}O_{13}$ calc./obs. 1938.22/1938.22 $[M]^+$.

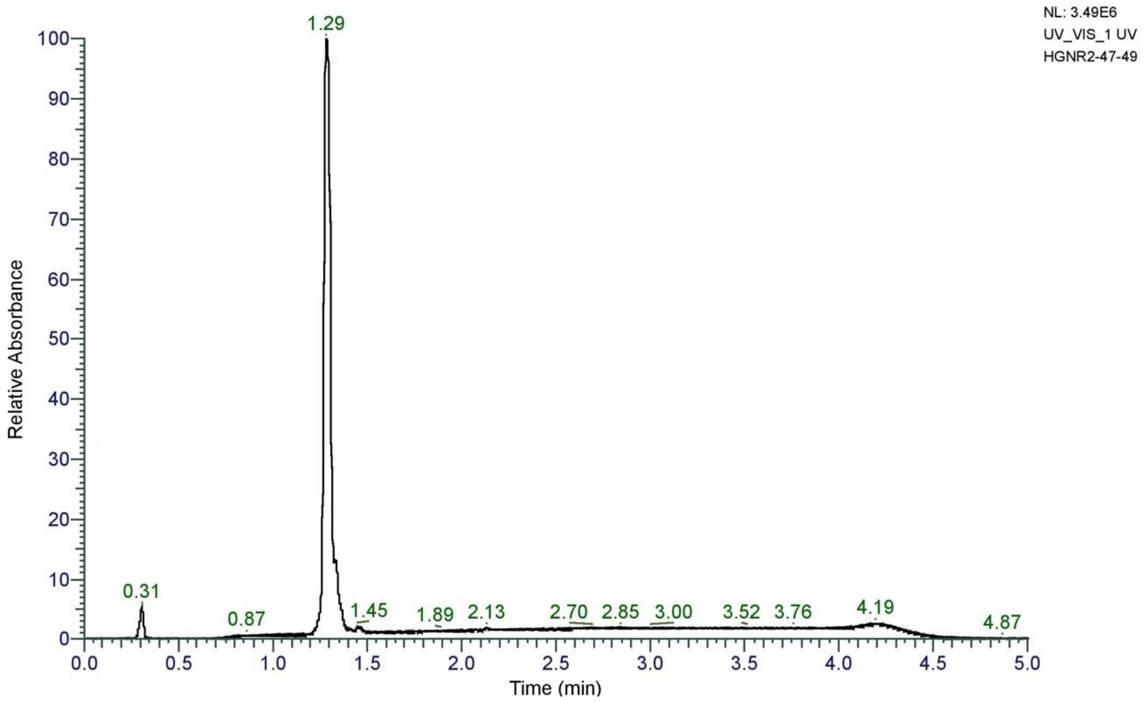

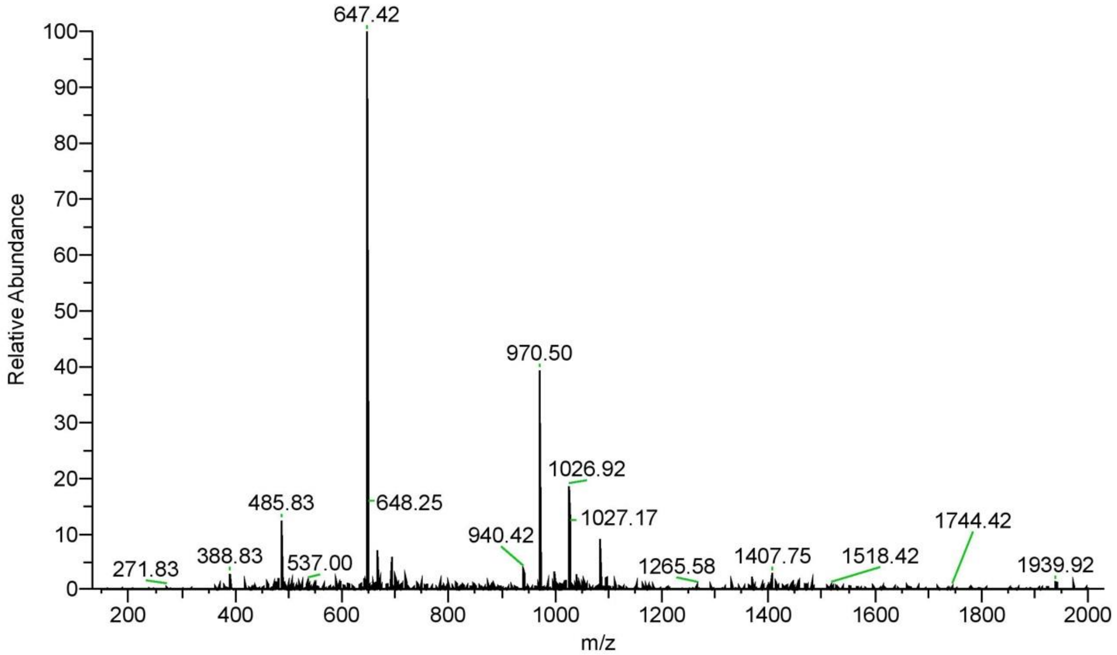

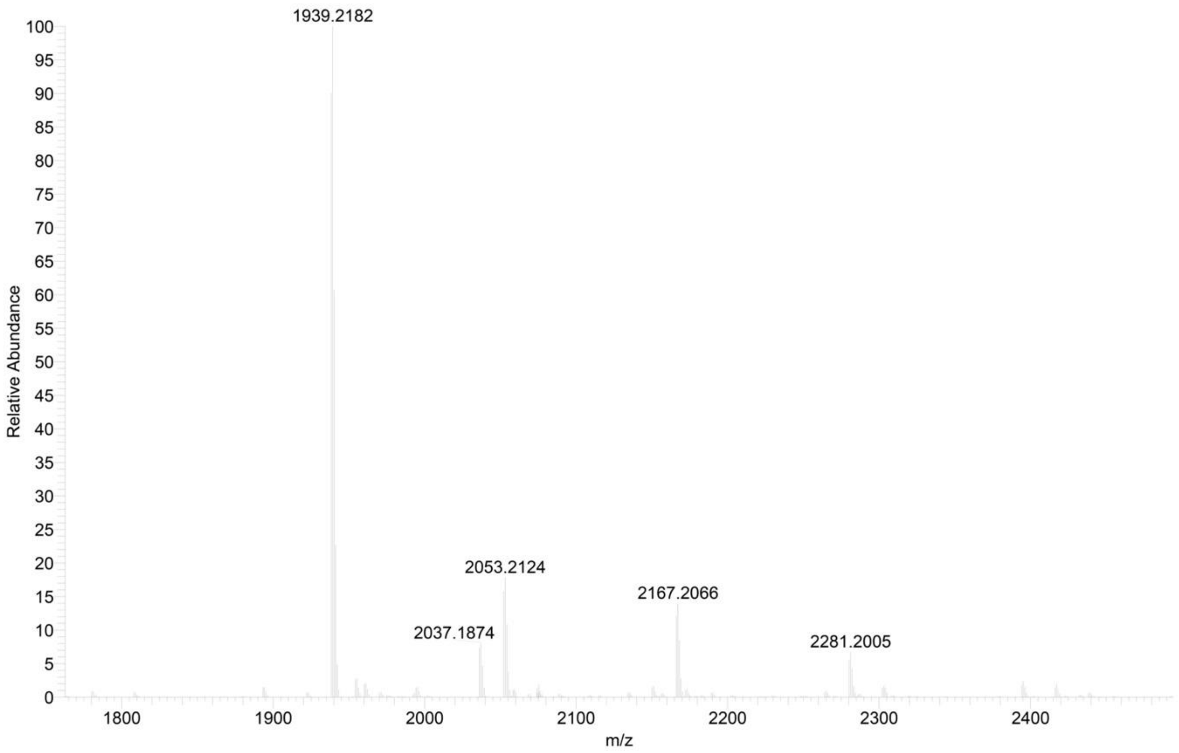
1939.2182
2053.2124
2167.2066
2037.1874
2281.2005
Relative Abundance
m/z

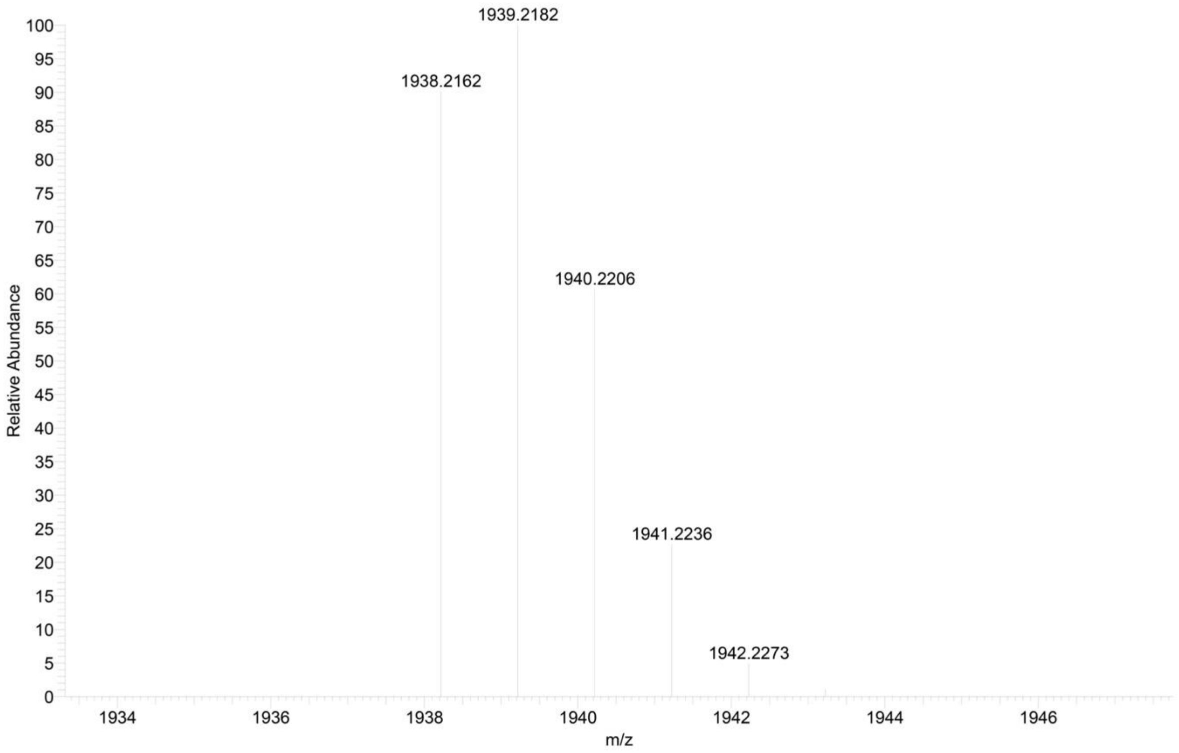
1939.2182
1938.2162
1940.2206
1941.2236
1942.2273
Relative Abundance
m/z

GN3 (IDKWKAAFKKIKNLF-NH_2) was obtained from Rink Amide AM resin LL (400 mg, 0.26 mmol/g), the peptide was obtained as a white foamy solid after preparative RP-HPLC purification (263.4 mg, 4.9%). Analytical RP-HPLC: t_R = 1.48 min (100% A to 100% D in 3.5 min, λ= 214 nm). MS (ESI+): $C_{91}H_{145}N_{23}O_{18}$ calc./obs. 1848.11/1848.11 $[M]^+$.

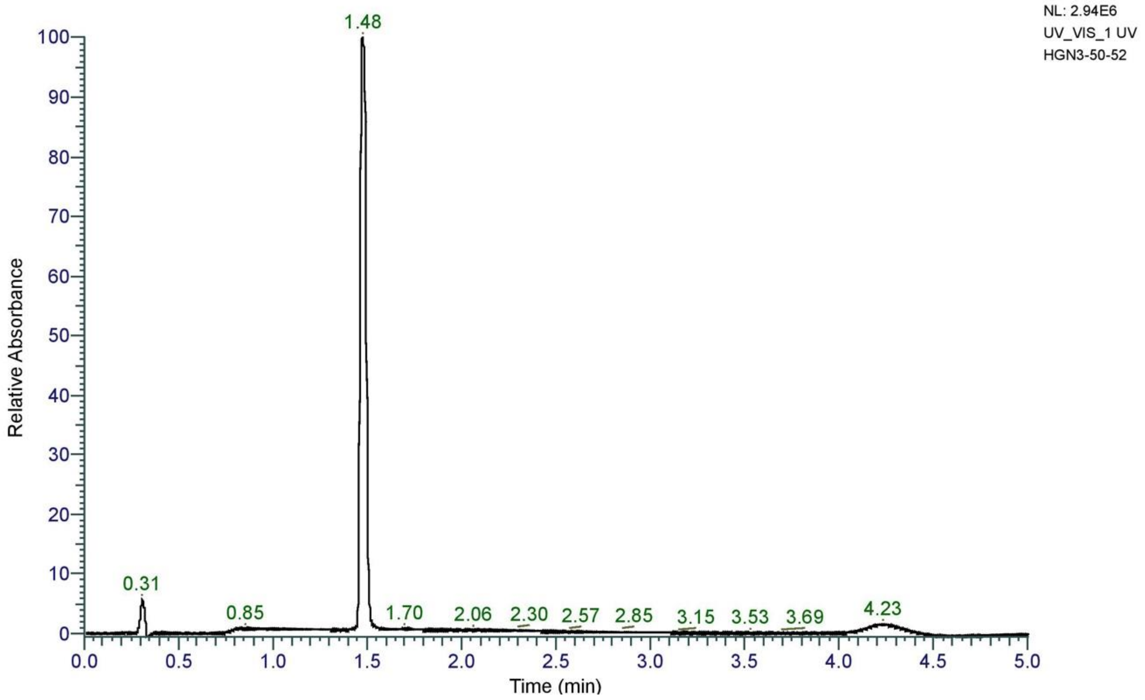

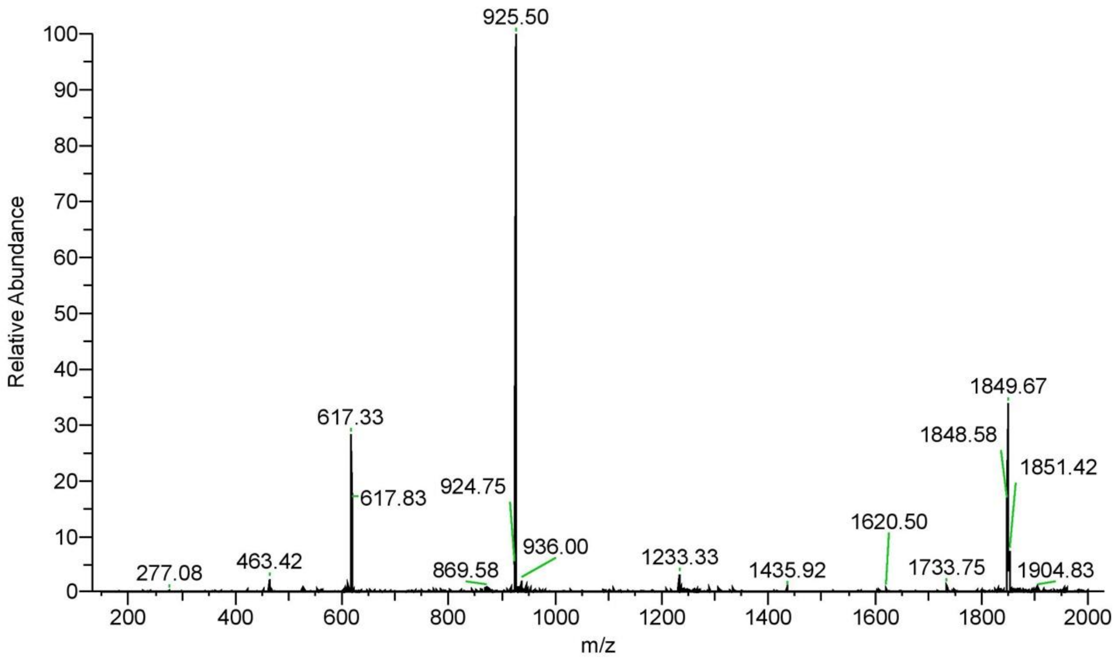

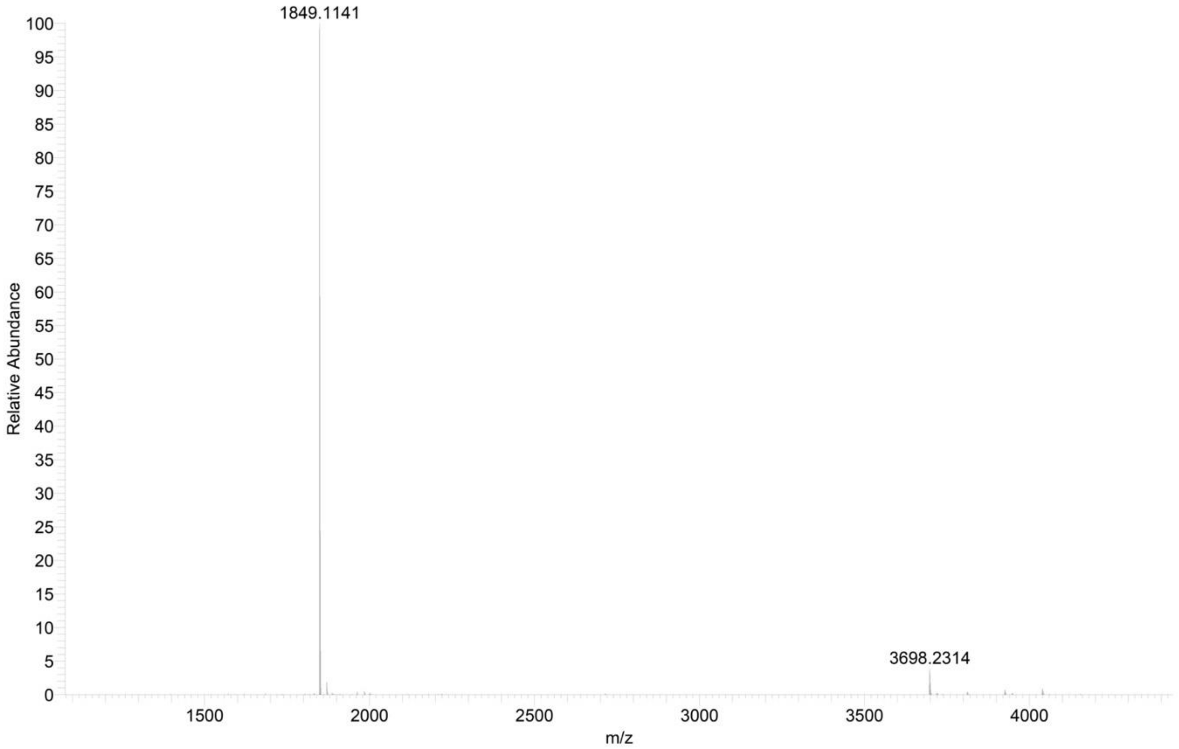
1849.1141
3698.2314
Relative Abundance
m/z

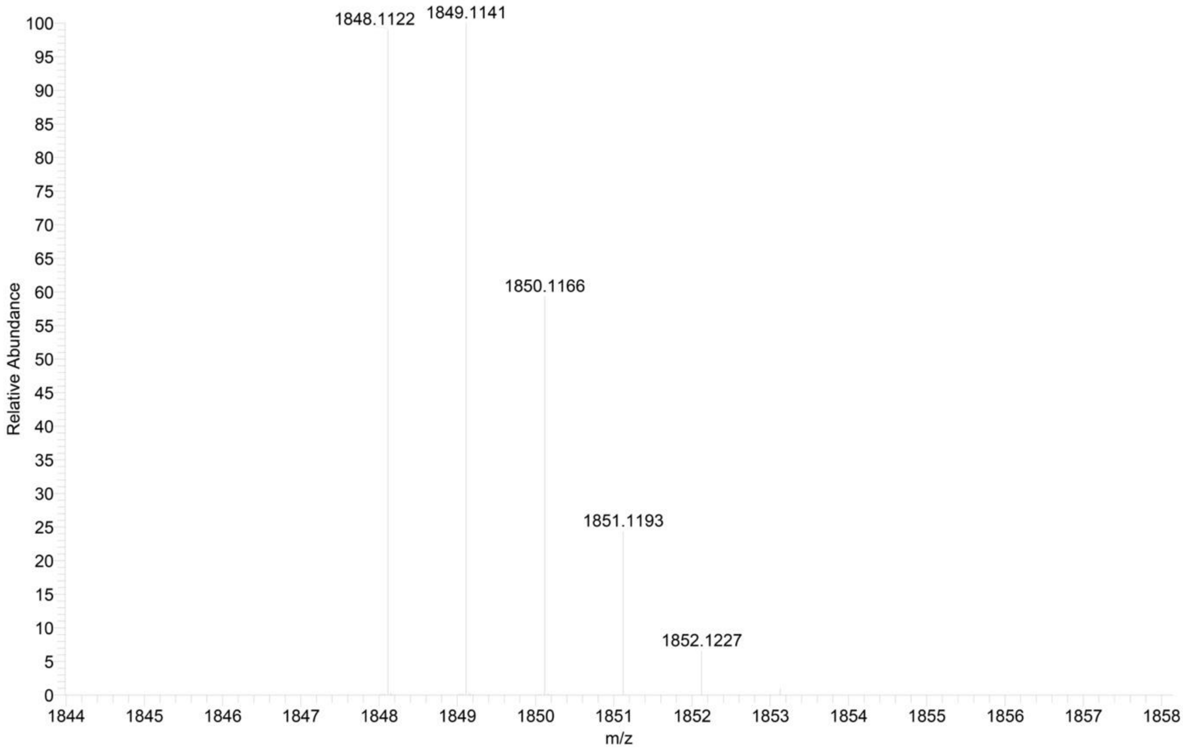
1848.1122
1849.1141
1850.1166
1851.1193
1852.1227
Relative Abundance
m/z

GN4 (LNALKKVFQKIRQGL-NH_2) was obtained from Rink Amide AM resin LL (400 mg, 0.26 mmol/g), the peptide was obtained as a white foamy solid after preparative RP-HPLC purification (241.8 mg, 28.8%). Analytical RP-HPLC: t_R = 1.53 min (100% A to 100% D in 3.5 min, λ= 214 nm). MS (ESI+): $C_{81}H_{143}N_{25}O_{18}$ calc./obs. 1745.10/1745.11 $[M]^+$.

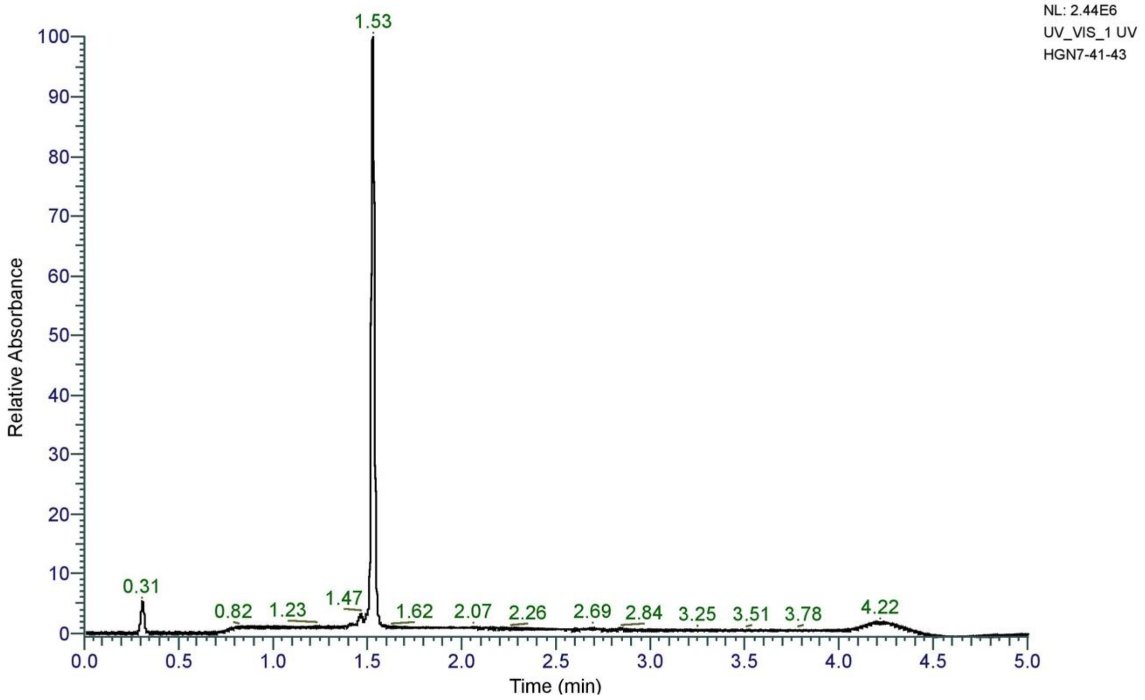

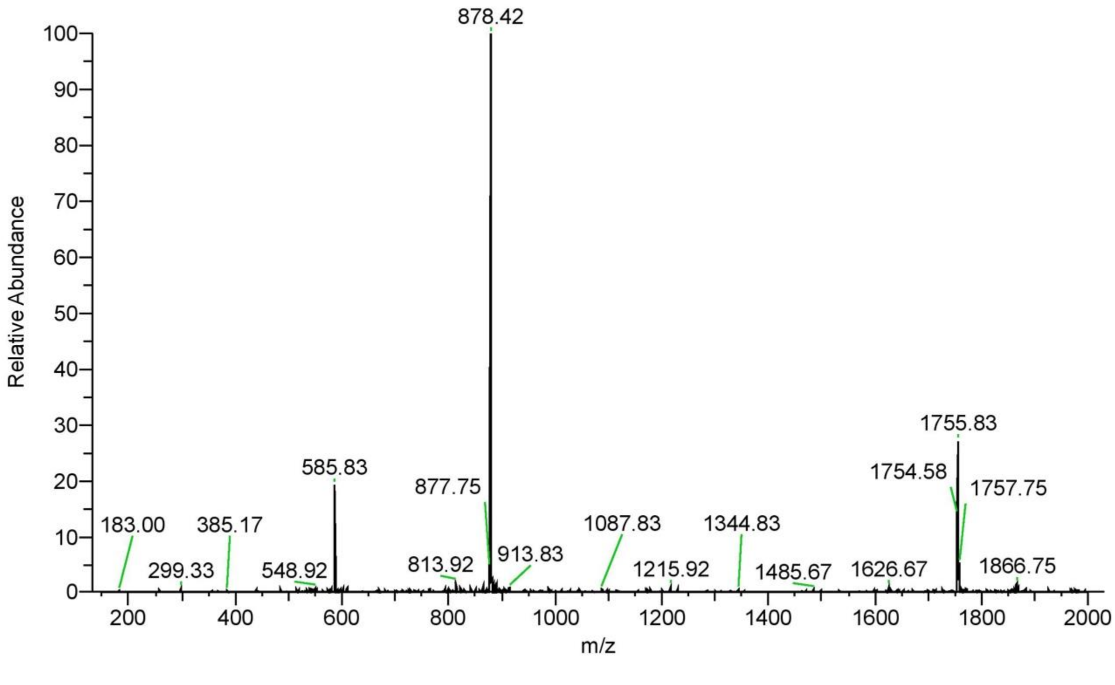

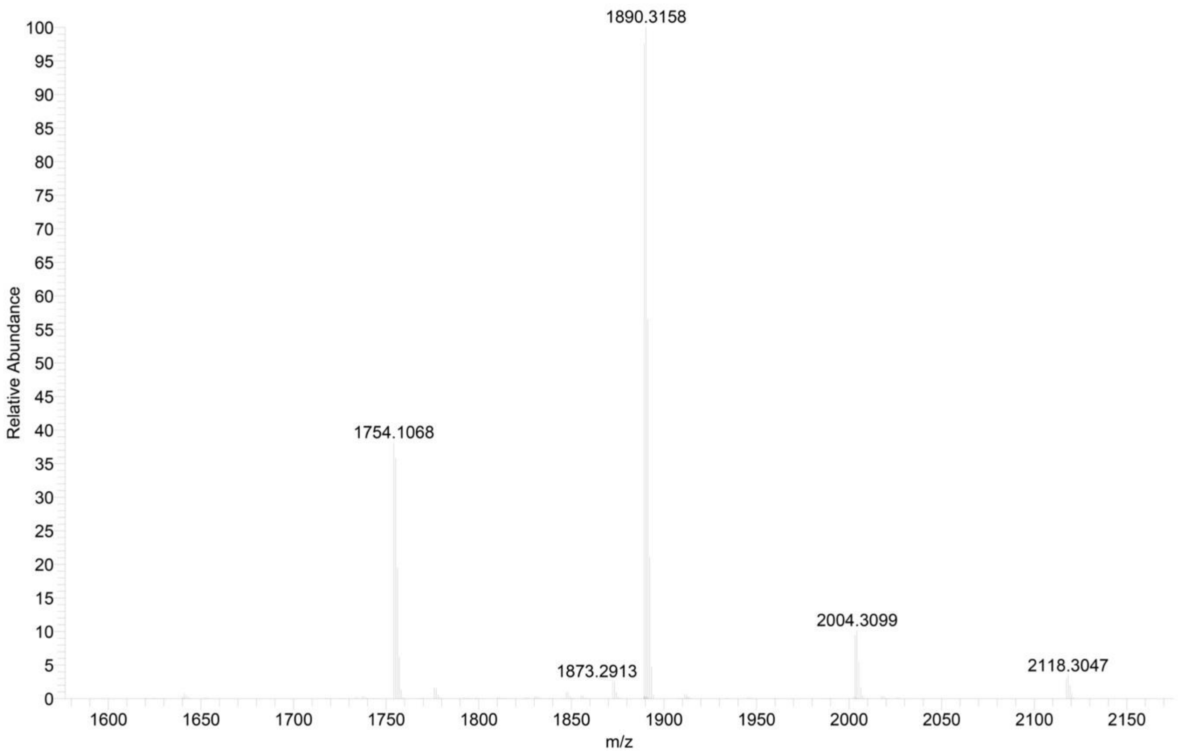
1890.3158
1754.1068
1873.2913
2004.3099
2118.3047
Relative Abundance
m/z

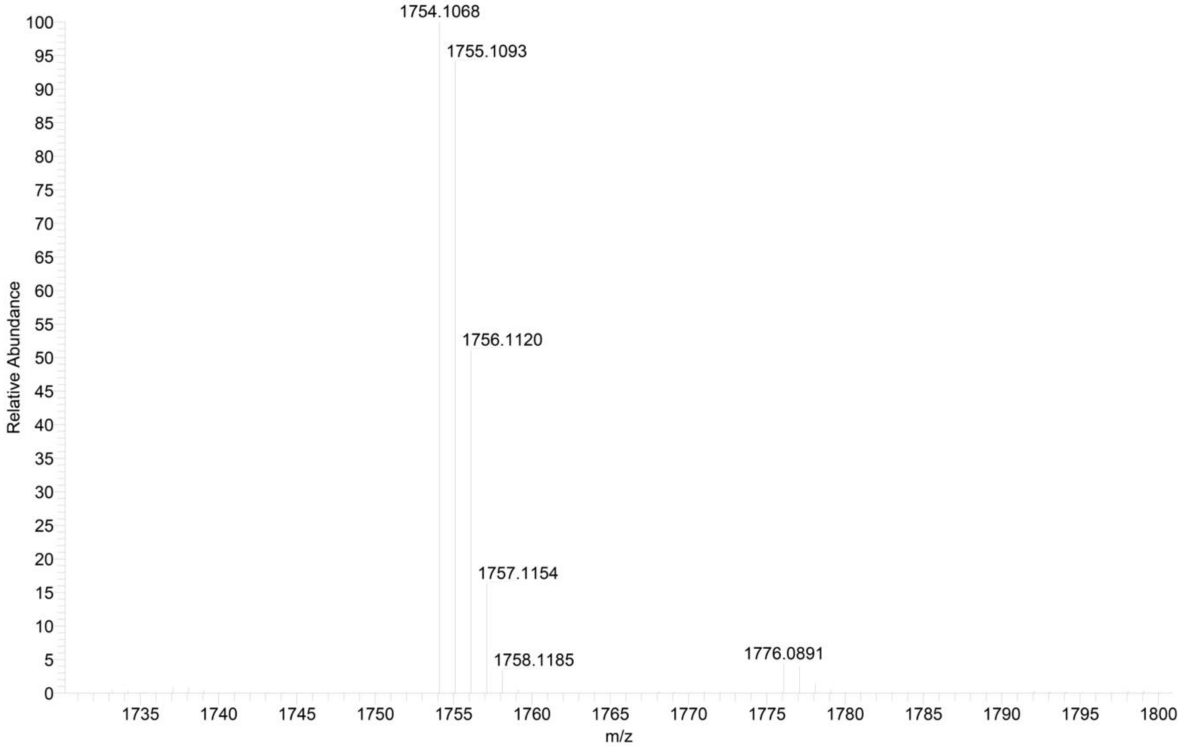
1754.1068
1755.1093
1756.1120
1757.1154
1758.1185
1776.0891
Relative Abundance
m/z

GN5 (KFFRKLKKLVKK-NH_2) was obtained from Rink Amide AM resin LL (400 mg, 0.26 mmol/g), the peptide was obtained as a white foamy solid after preparative RP-HPLC purification (257.3 mg, 33.5%). Analytical RP-HPLC: t_R = 1.24 min (100% A to 100% D in 3.5 min, λ= 214 nm). MS (ESI+): $C_{77}H_{136}N_{22}O_{12}$ calc./obs. 1561.07/1561.07 $[M]^+$.

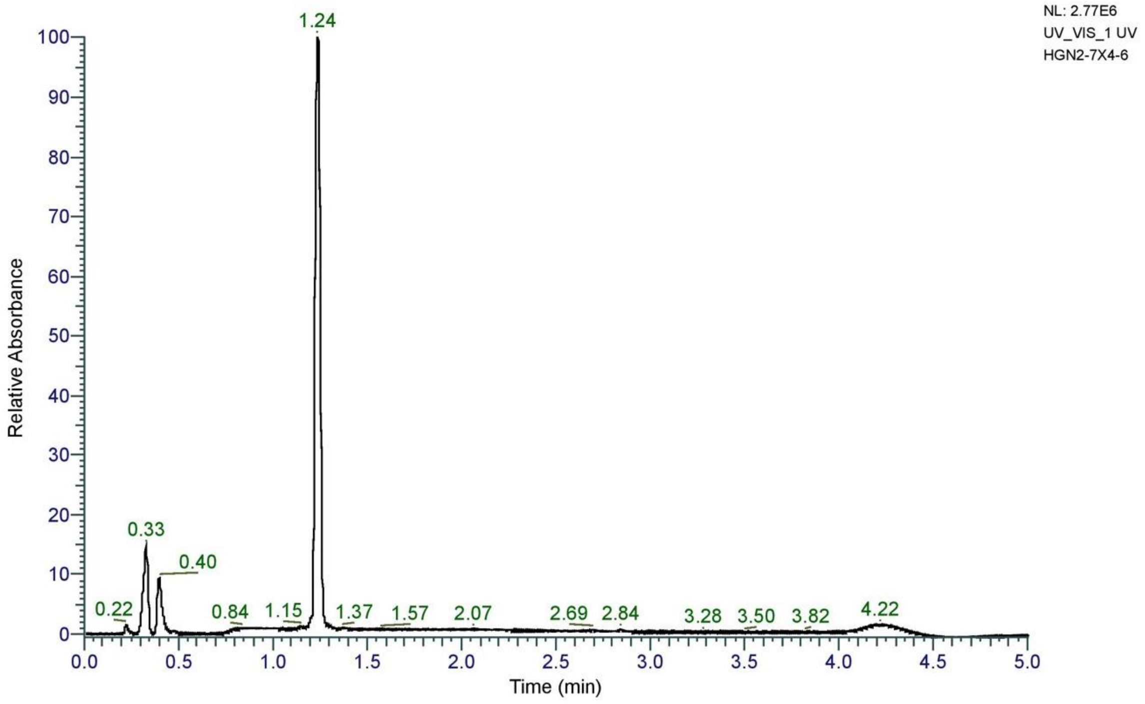

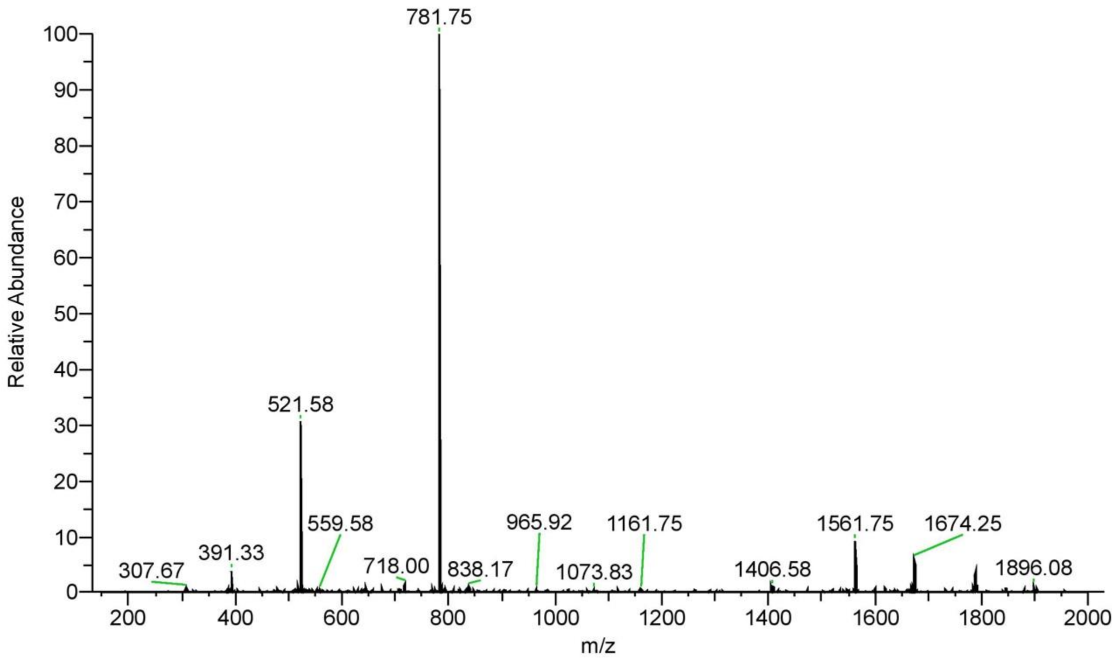

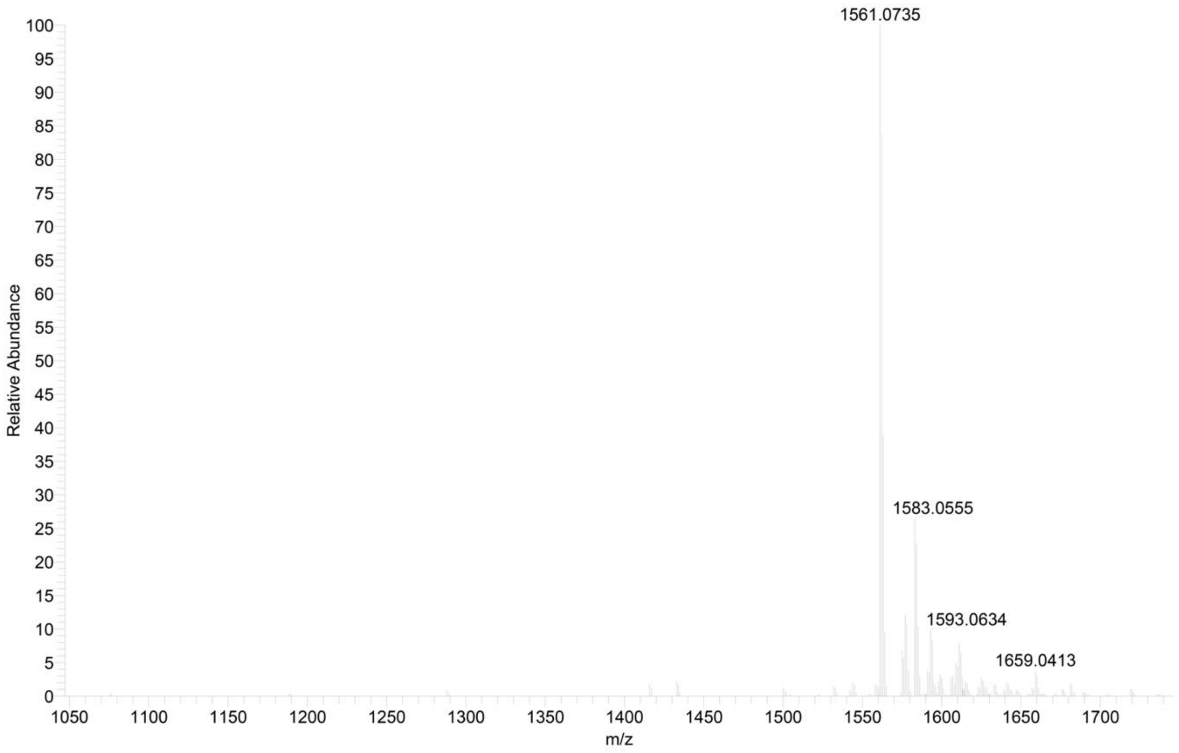
1561.0735
1583.0555
1593.0634
1659.0413
Relative Abundance
m/z

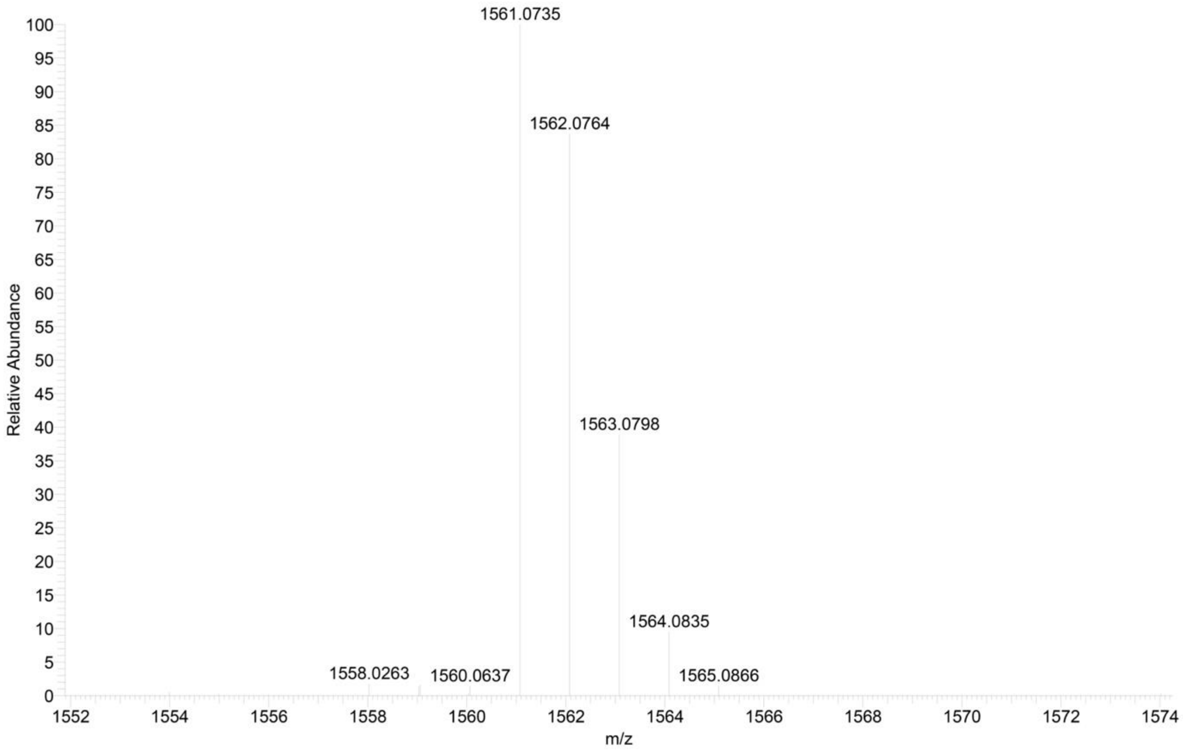
1561.0735
1562.0764
1563.0798
1564.0835
1558.0263
1560.0637
1565.0866
Relative Abundance
m/z

GN6 (RLRKKWRKLKKLL-NH_2) was obtained from Rink Amide AM resin LL (400 mg, 0.26 mmol/g), the peptide was obtained as a white foamy solid after preparative RP-HPLC purification (290.3 mg, 31.4%). Analytical RP-HPLC: t_R = 1.30 min (100% A to 100% D in 3.5 min, λ= 214 nm). MS (ESI+): $C_{83}H_{153}N_{29}O_{13}$ calc./obs. 1764.22/1764.22 $[M]^+$.

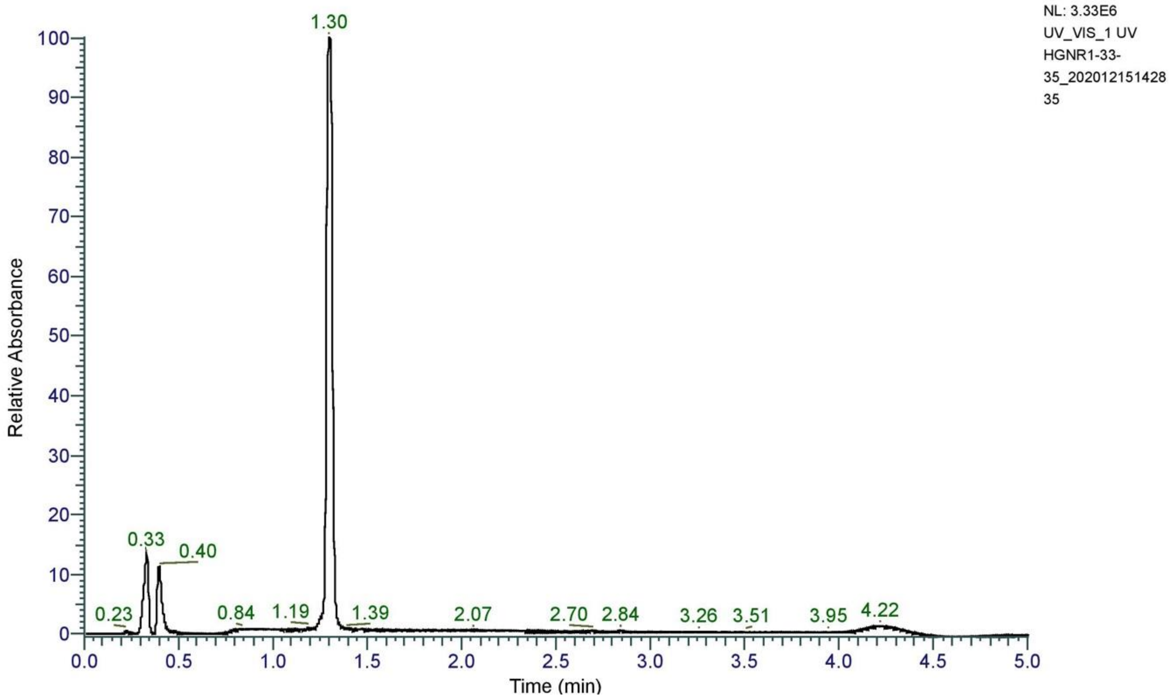

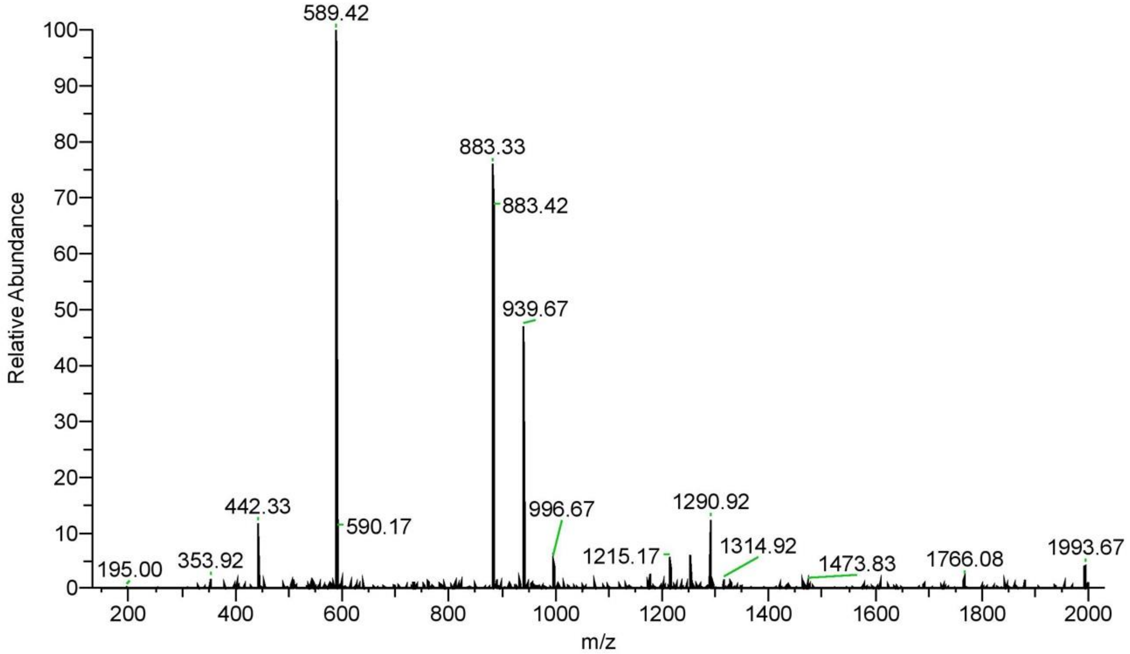

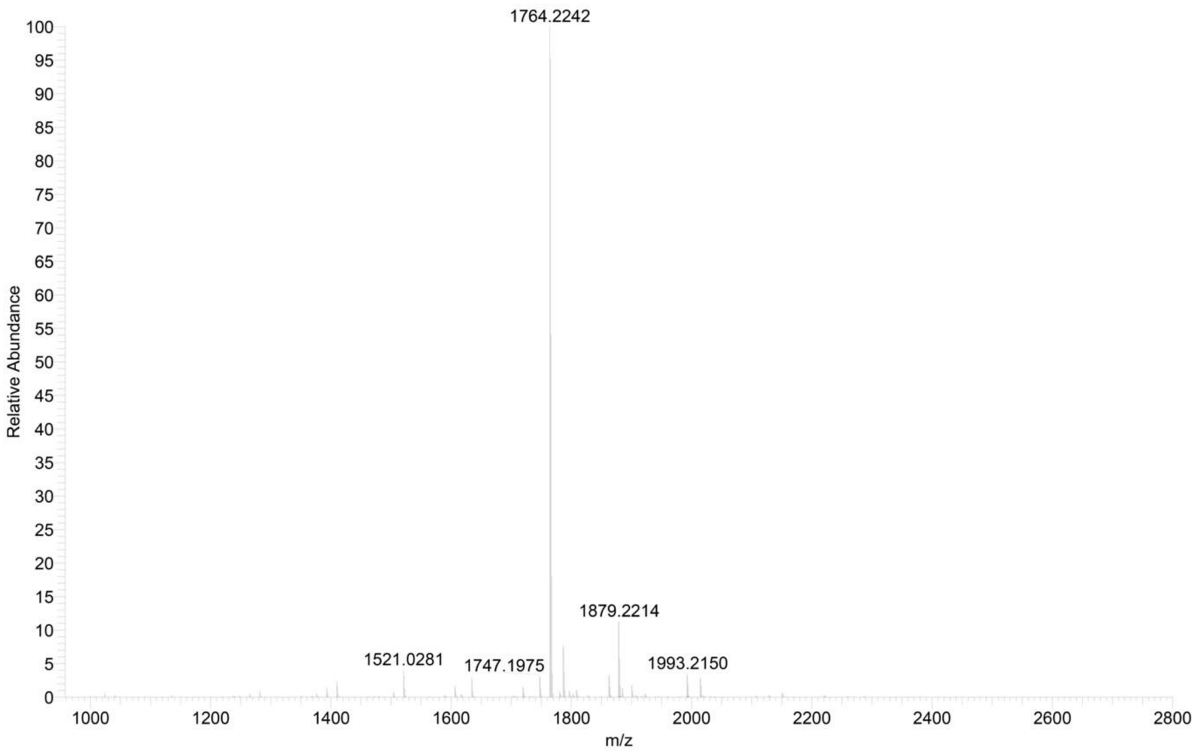
1764.2242
1879.2214
1521.0281
1747.1975
1993.2150
Relative Abundance
m/z

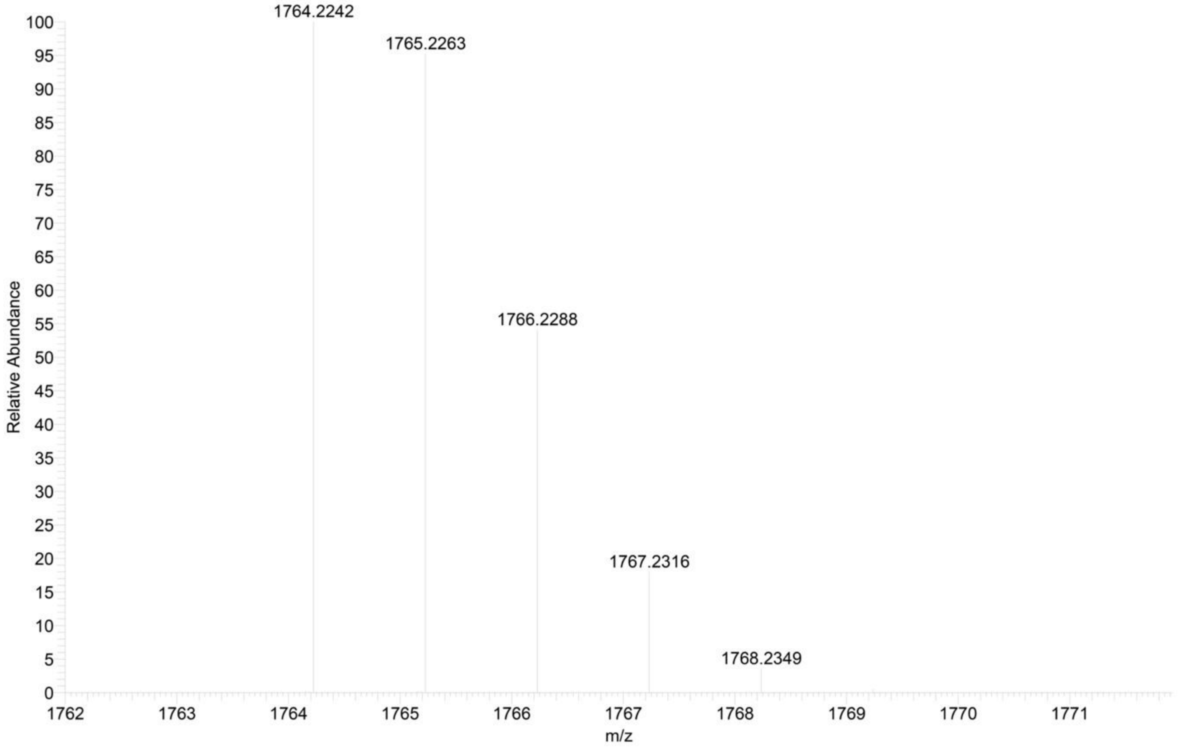
1764.2242
1765.2263
1766.2288
1767.2316
1768.2349
Relative Abundance
m/z

GN7 (KRIRKWVRRILKKL-NH_2) was obtained from Rink Amide AM resin LL (400 mg, 0.26 mmol/g), the peptide was obtained as a white foamy solid after preparative RP-HPLC purification (291.7 mg, 19.4%). Analytical RP-HPLC: t_R = 1.37 min (100% A to 100% D in 3.5 min, λ= 214 nm). MS (ESI+): $C_{88}H_{162}N_{32}O_{14}$ calc./obs. 1891.29/1891.30 $[M]^+$.

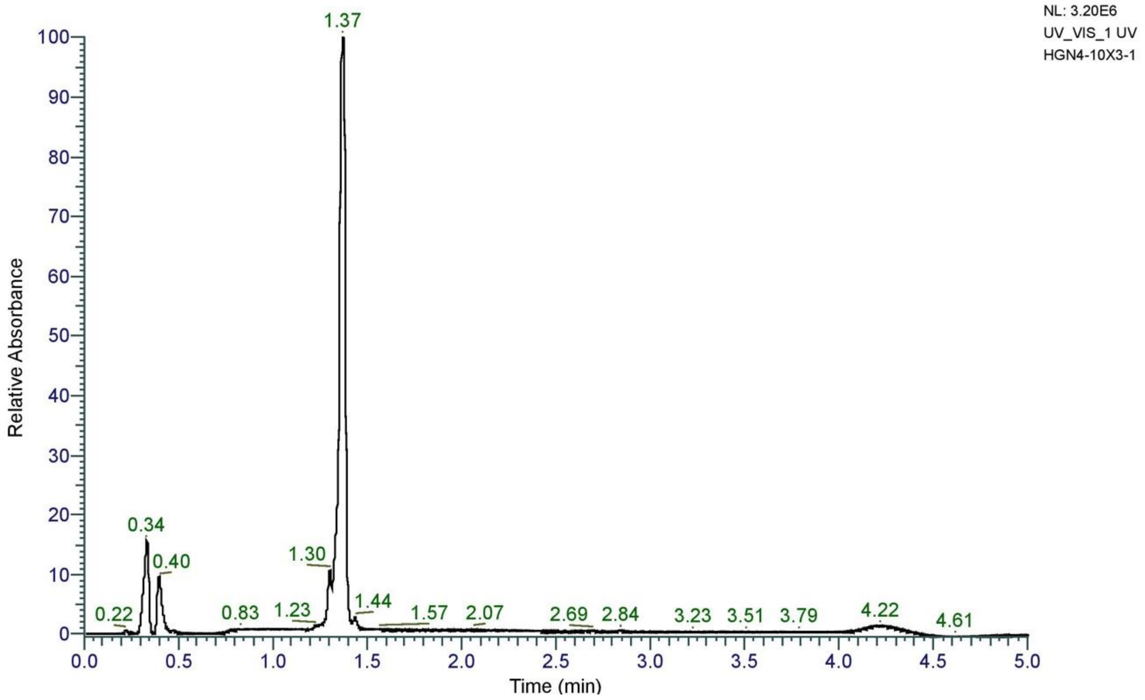

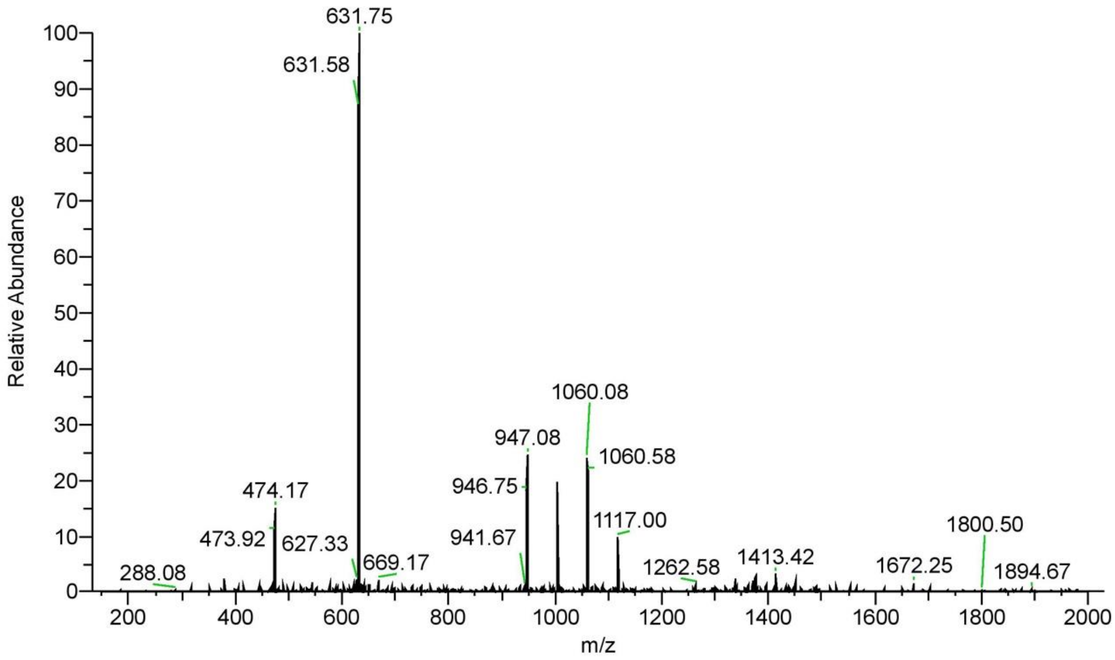

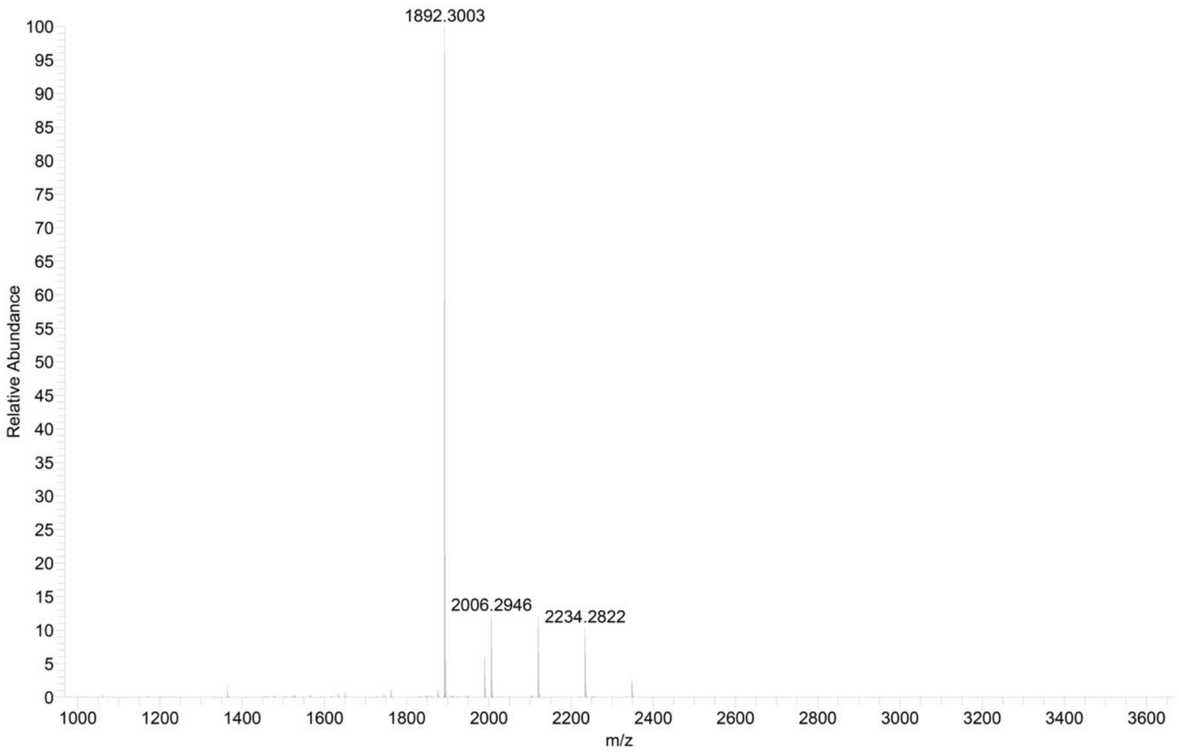
1892.3003
2006.2946
2234.2822
Relative Abundance
m/z

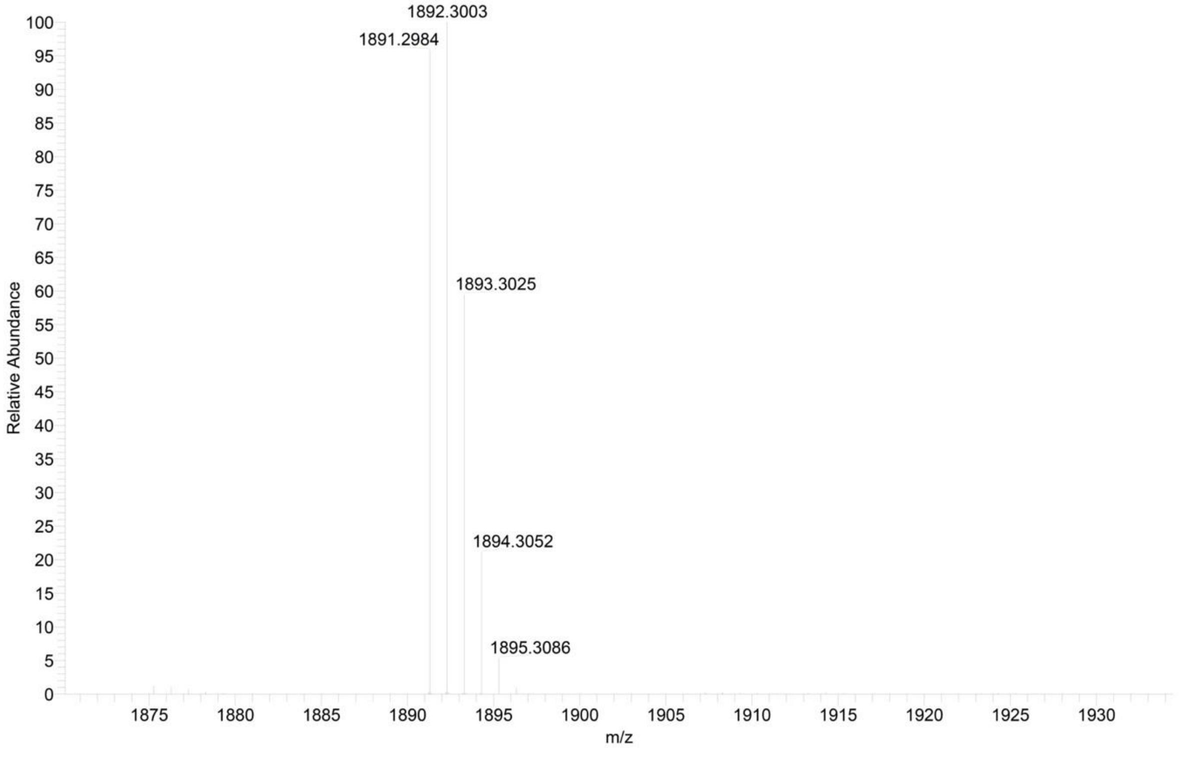
1892.3003
1891.2984
1893.3025
1894.3052
1895.3086
Relative Abundance
m/z

GN8 (LRKFWKKIRKFLKKI-NH_2) was obtained from Rink Amide AM resin LL (400 mg, 0.26 mmol/g), the peptide was obtained as a white foamy solid after preparative RP-HPLC purification (372.0 mg, 17.2%). Analytical RP-HPLC: t_R = 1.46 min (100% A to 100% D in 3.5 min, λ= 214 nm). MS (ESI+): $C_{101}H_{171}N_{29}O_{15}$ calc./obs. 2030.35/2030.35 $[M]^+$.

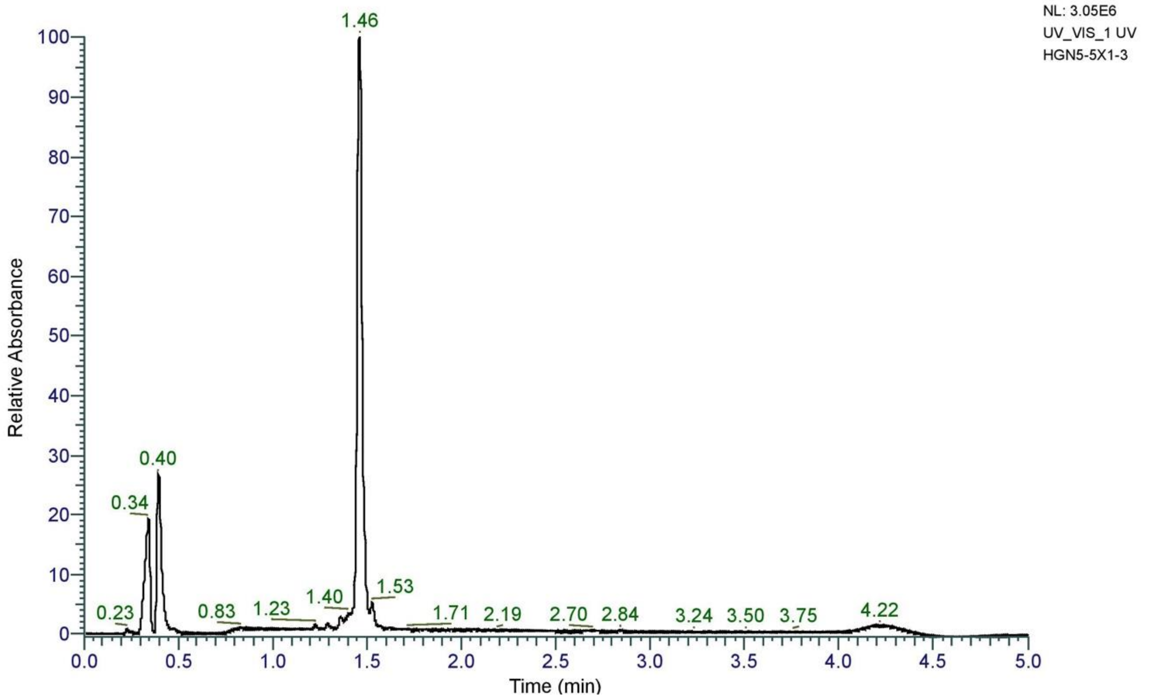

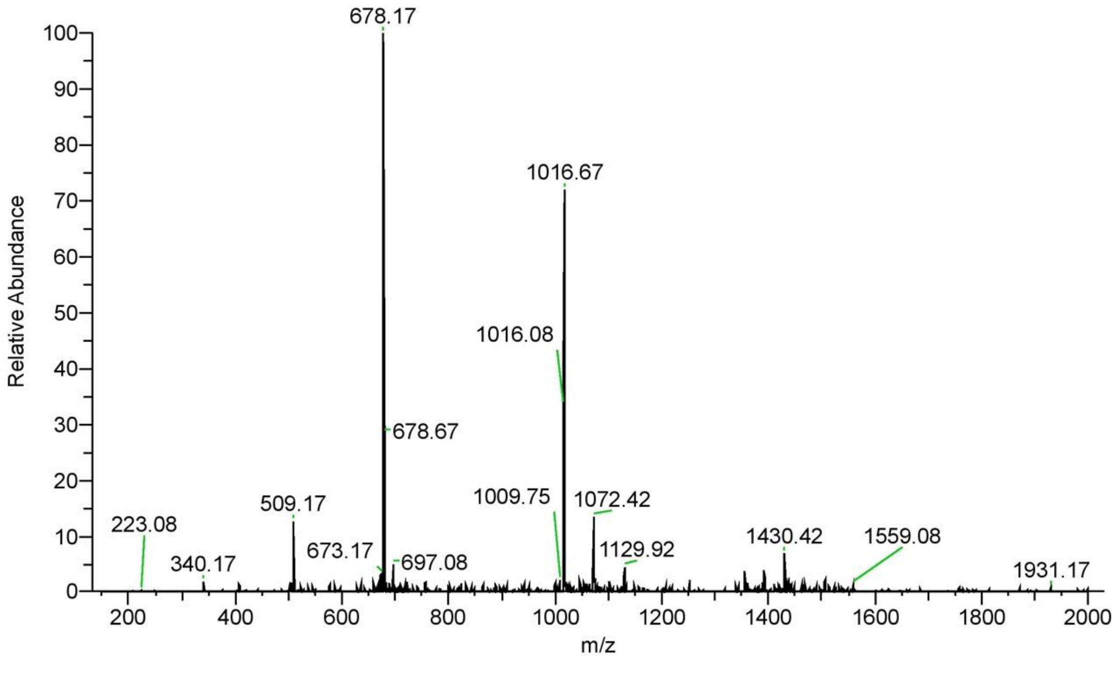

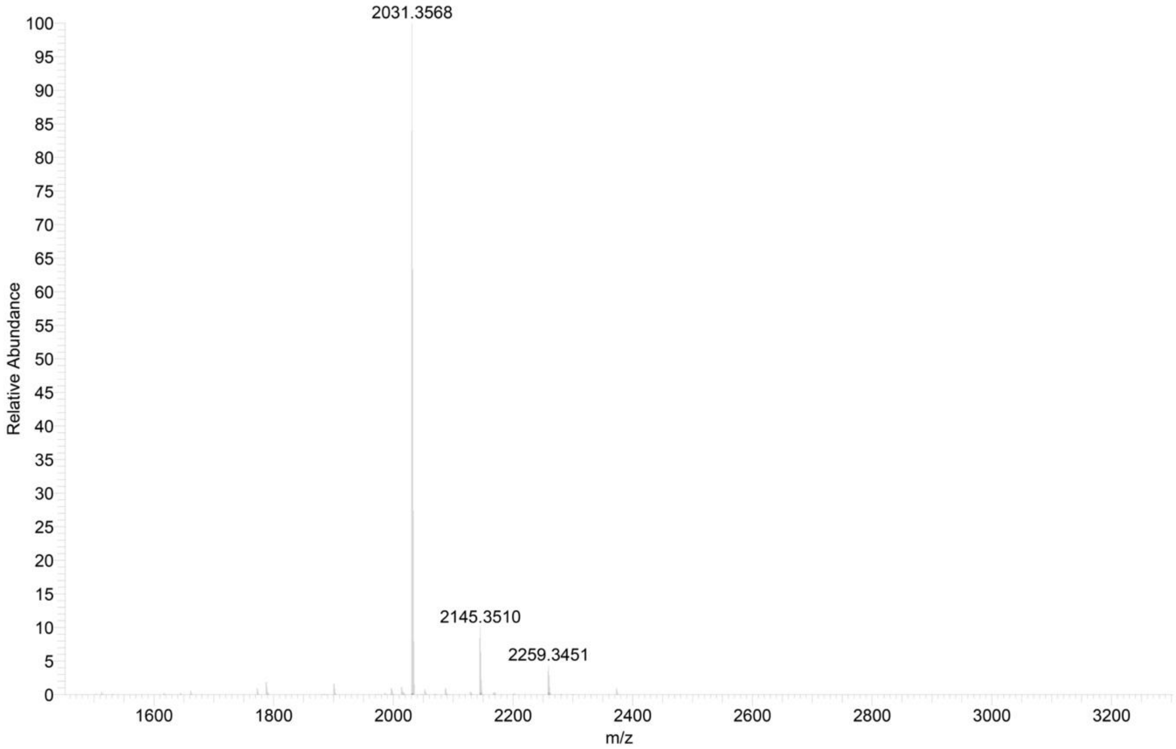
2031.3568
2145.3510
2259.3451
Relative Abundance
m/z

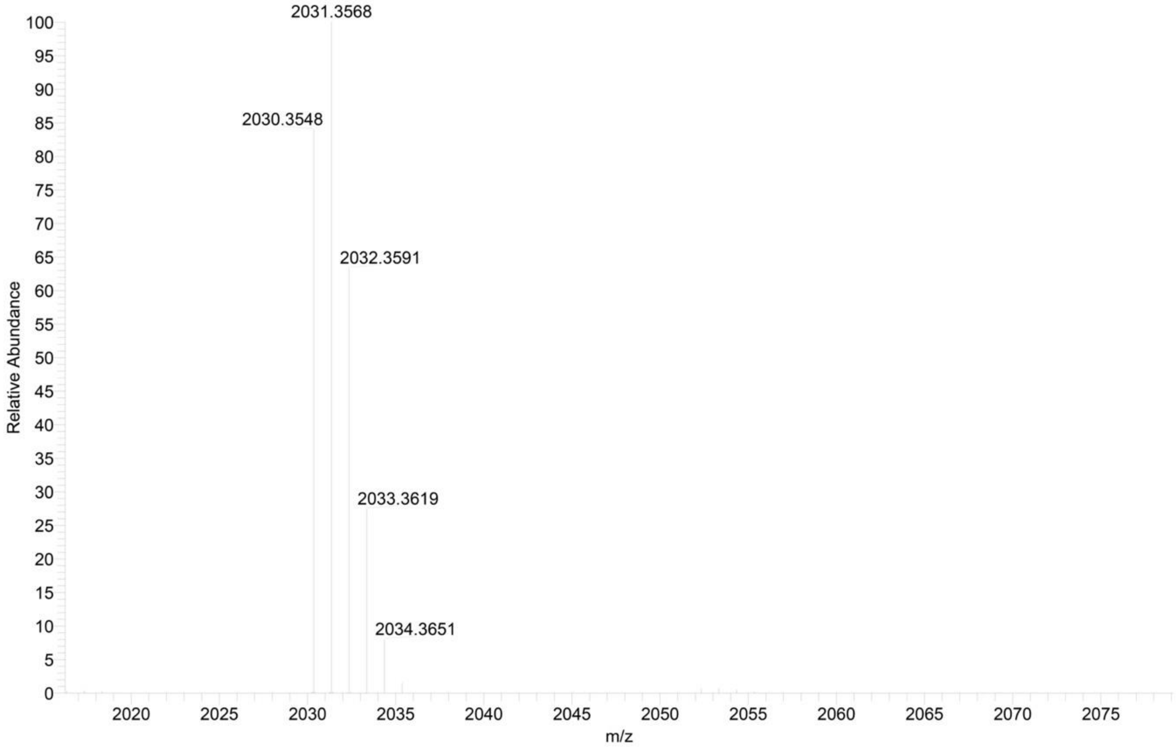
2031.3568
2030.3548
2032.3591
2033.3619
2034.3651
Relative Abundance
m/z

GN9 (KRLWKRIYRLLKK-NH_2) was obtained from Rink Amide AM resin LL (400 mg, 0.26 mmol/g), the peptide was obtained as a white foamy solid after preparative RP-HPLC purification (282.0 mg, 8.8%). Analytical RP-HPLC: t_R = 1.36 min (100% A to 100% D in 3.5 min, λ= 214 nm). MS (ESI+): $C_{86}H_{150}N_{28}O_{14}$ calc./obs. 1799.19/1799.18 $[M]^+$.

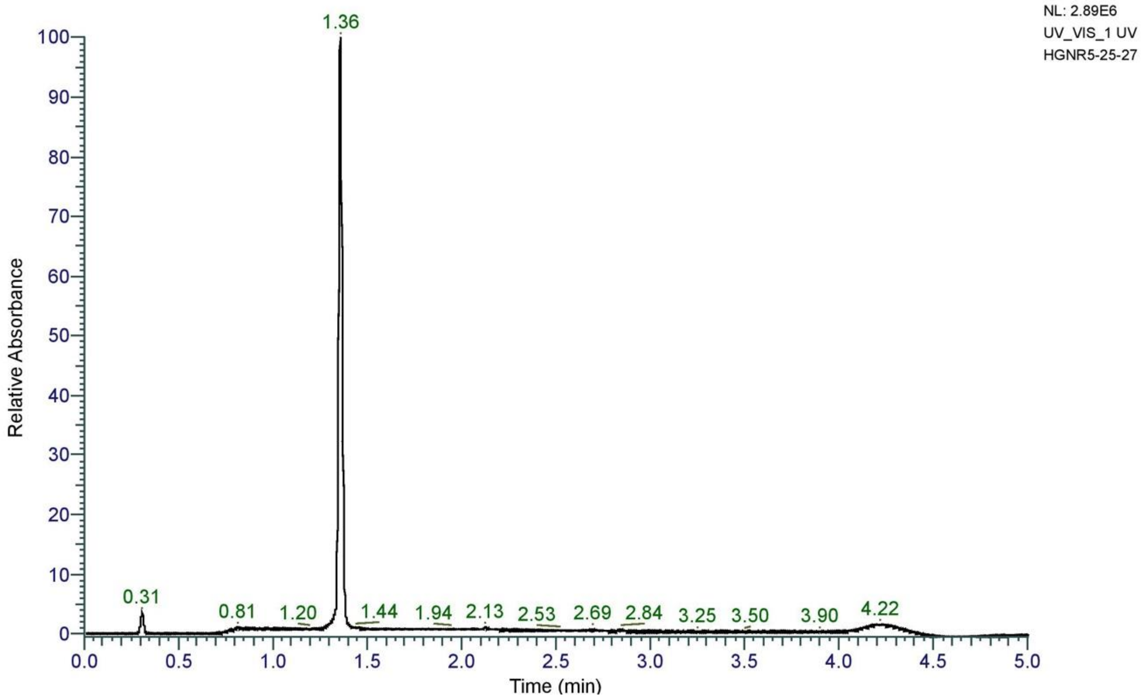

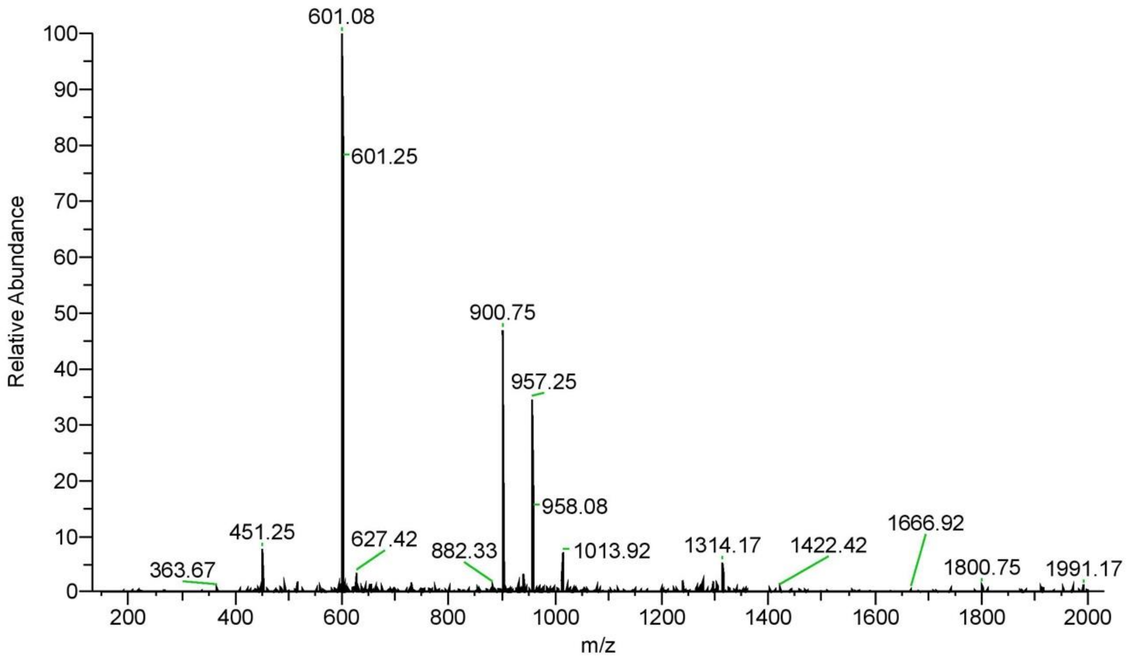

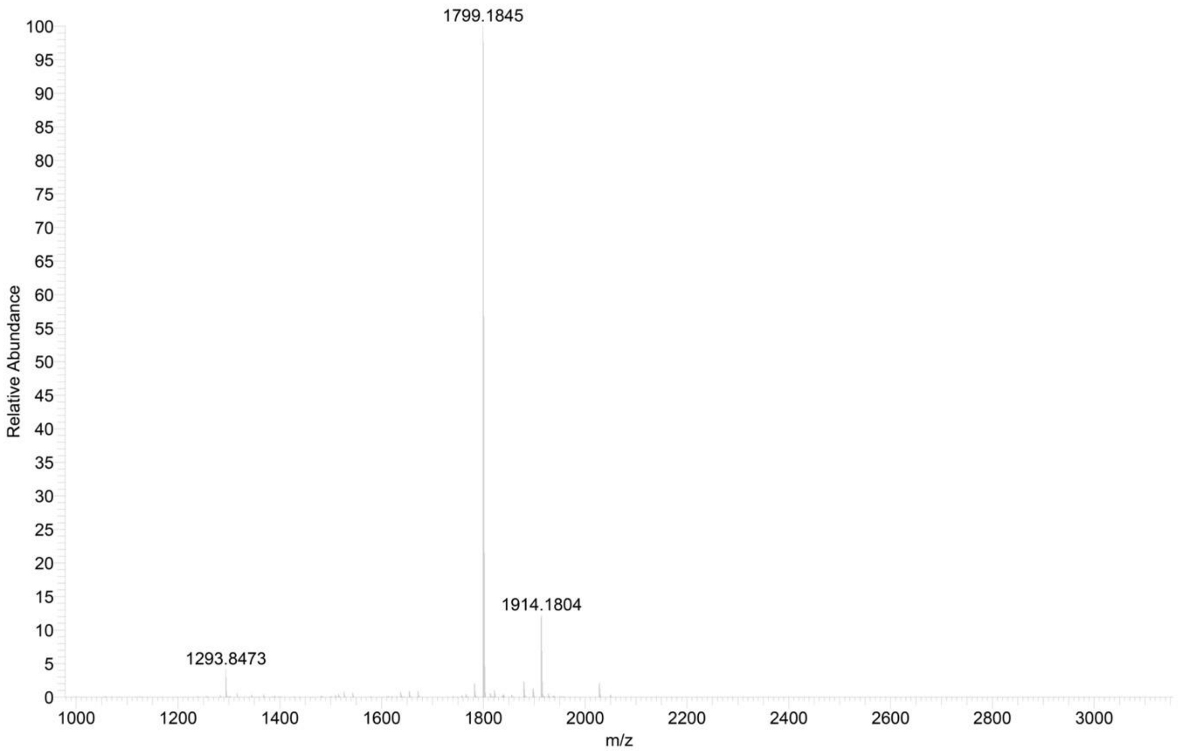
1799.1845
1914.1804
1293.8473
Relative Abundance
m/z

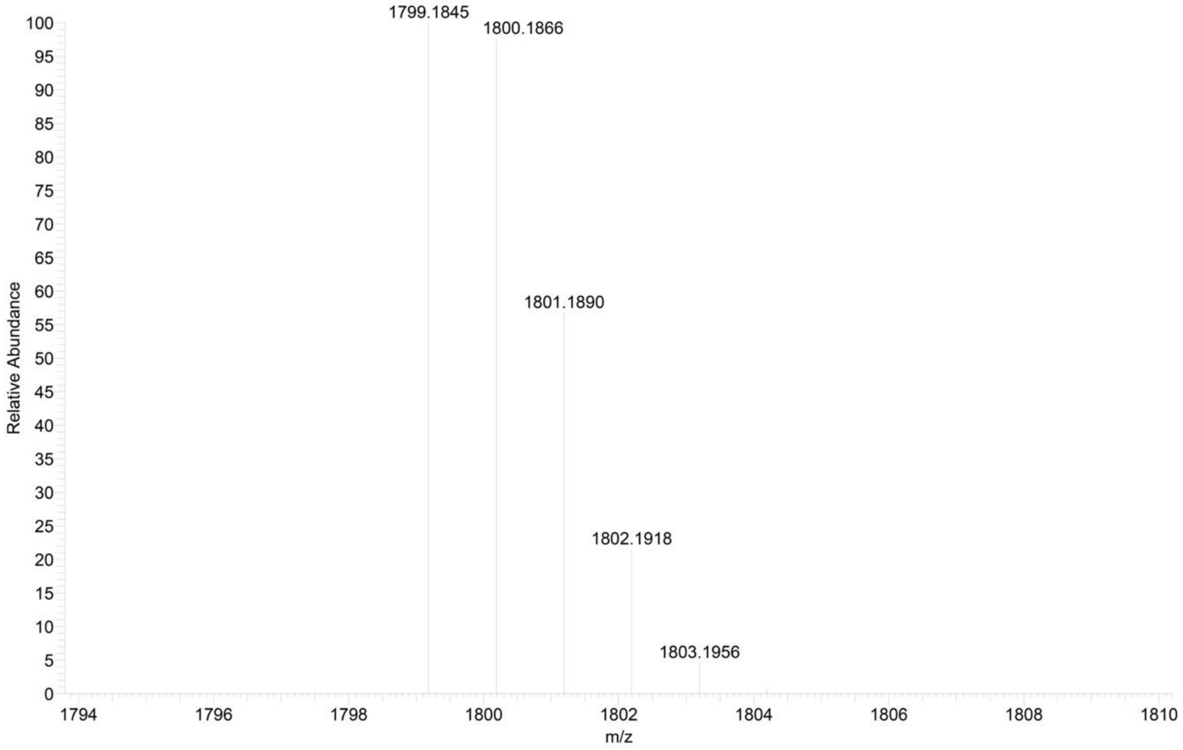
1799.1845
1800.1866
1801.1890
1802.1918
1803.1956
Relative Abundance
m/z

GN10 (IRRIRKKIKKIFKKI-NH_2) was obtained from Rink Amide AM resin LL (400 mg, 0.26 mmol/g), the peptide was obtained as a white foamy solid after preparative RP-HPLC purification (335.0 mg, 8.2%). Analytical RP-HPLC: t_R = 1.26 min (100% A to 100% D in 3.5 min, λ= 214 nm). MS (ESI+): $C_{93}H_{175}N_{31}O_{15}$ calc./obs. 1966.39/1966.39 $[M]^+$.

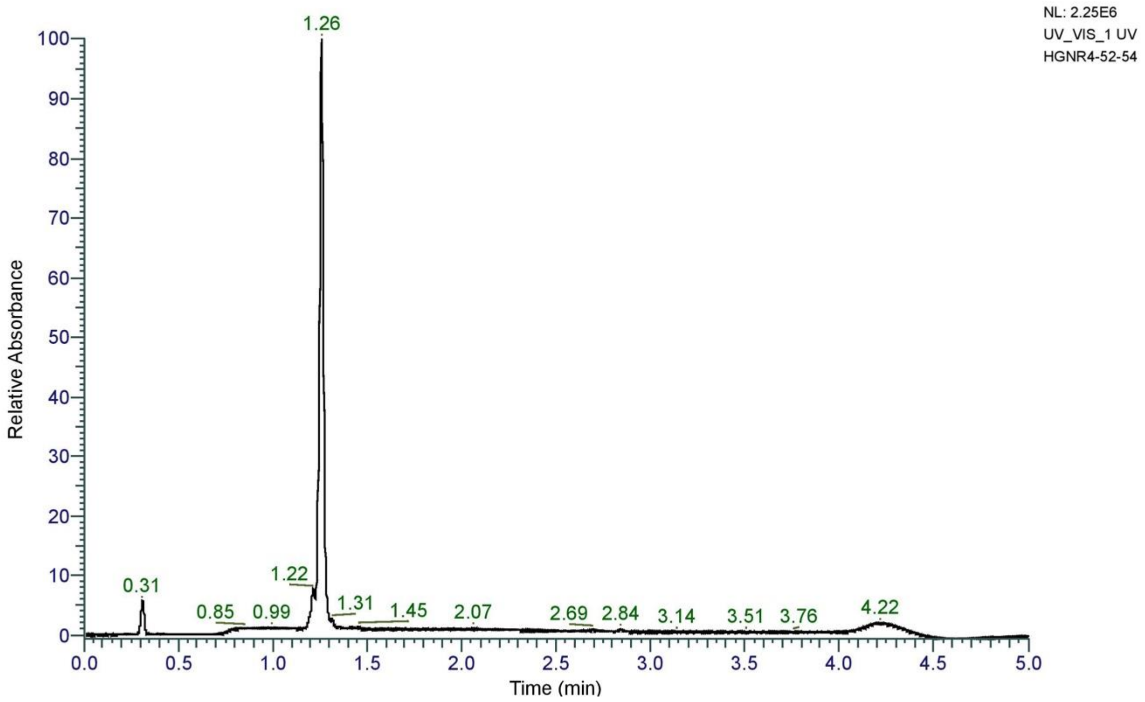

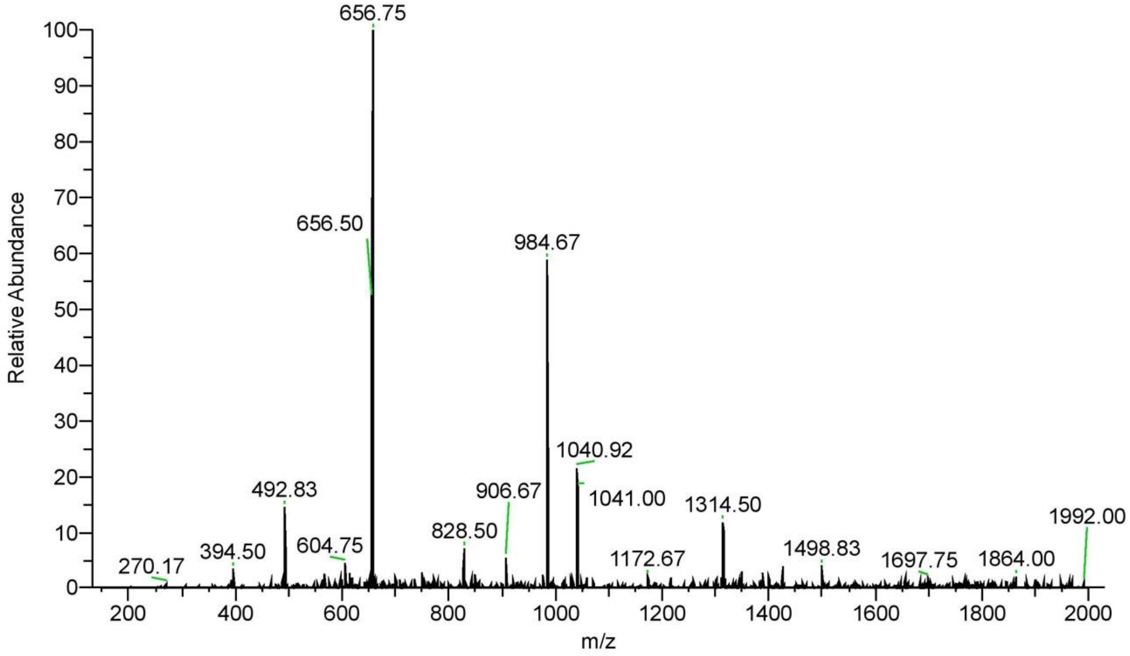

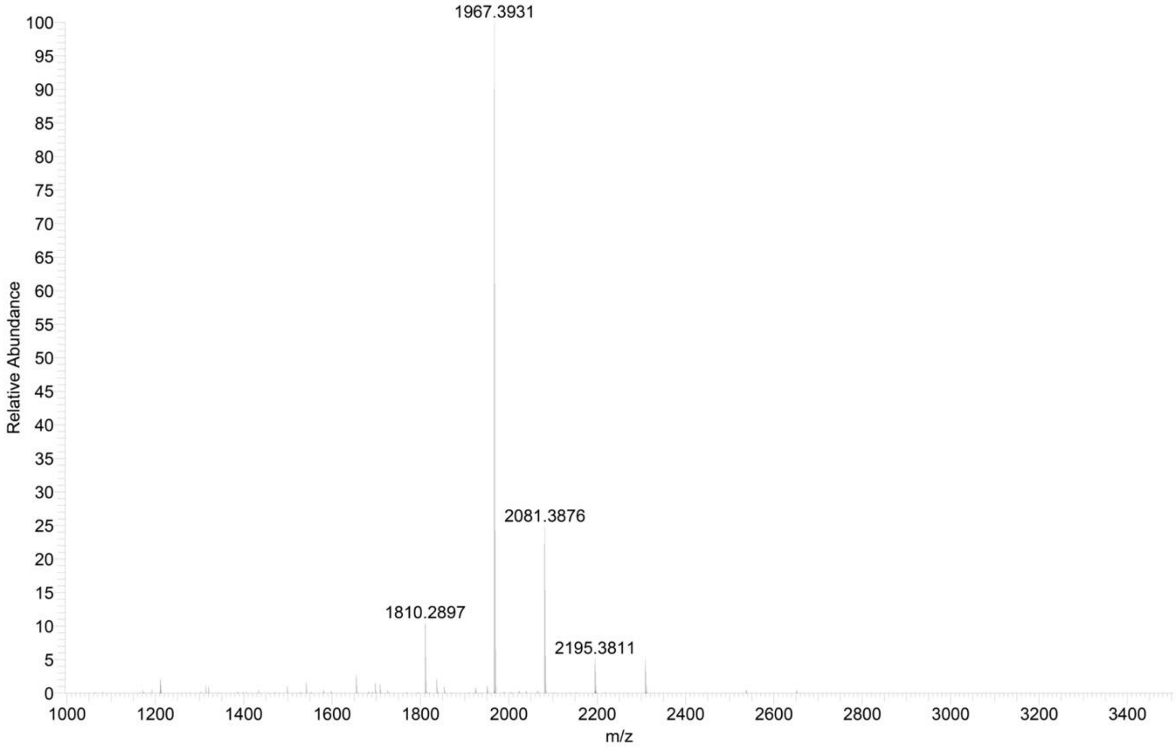
1967.3931
2081.3876
1810.2897
2195.3811
Relative Abundance
m/z

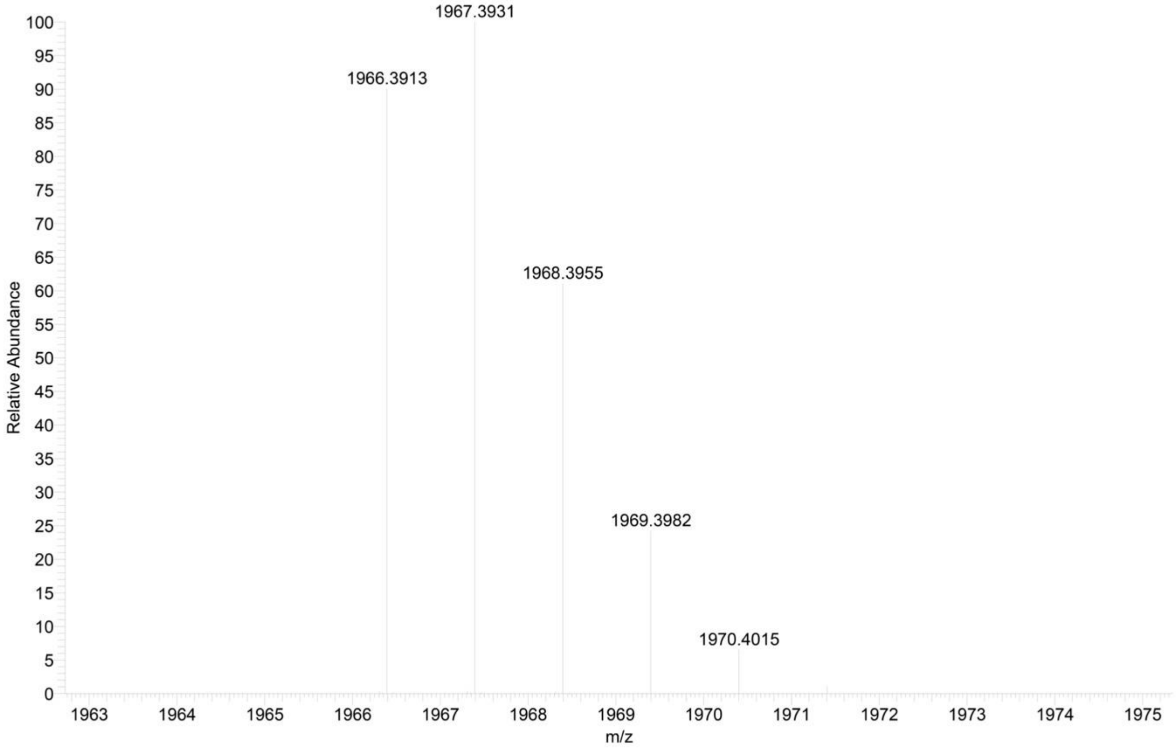
1967.3931
1966.3913
1968.3955
1969.3982
1970.4015
Relative Abundance
m/z

GN11 (LRKARRLLKKLRARL-NH_2) was obtained from Rink Amide AM resin LL (400 mg, 0.26 mmol/g), the peptide was obtained as a white foamy solid after preparative RP-HPLC purification (303.3 mg, 21.4%). Analytical RP-HPLC: t_R = 1.28 min (100% A to 100% D in 3.5 min, λ= 214 nm). MS (ESI+): $C_{84}H_{164}N_{34}O_{15}$ calc./obs. 1889.31/1889.31 $[M]^+$.

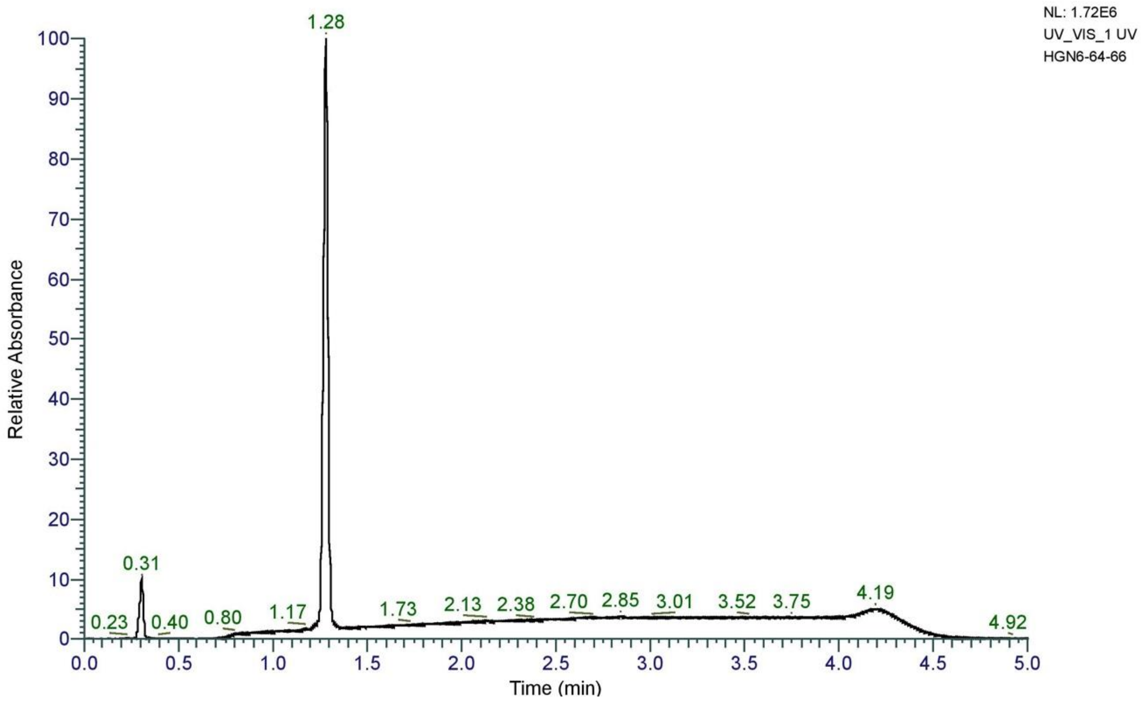

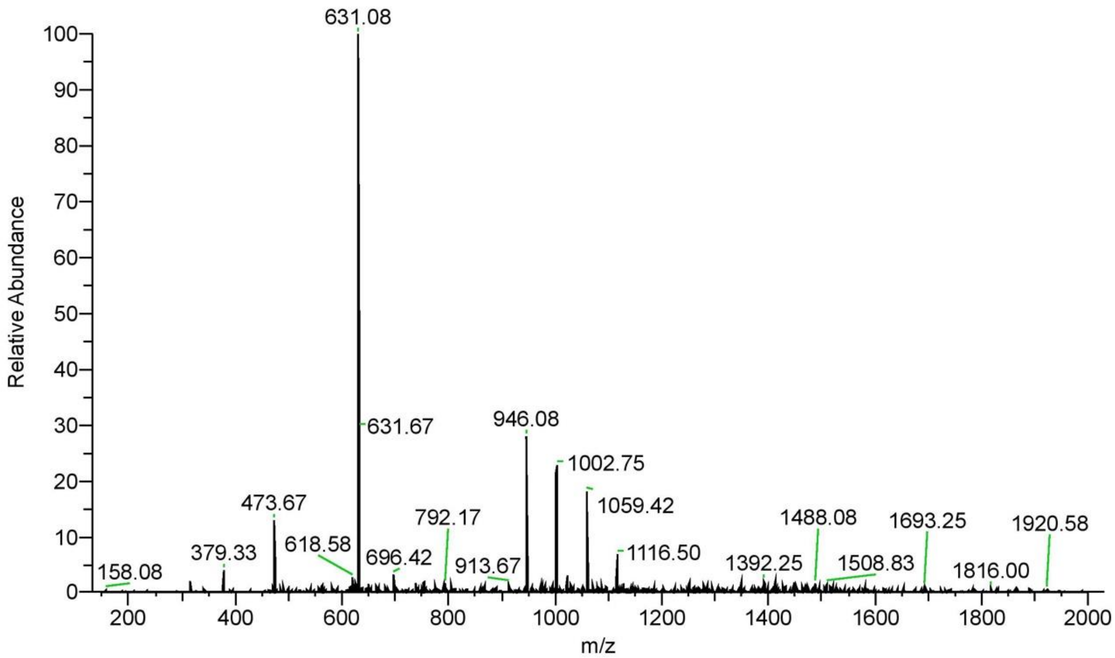

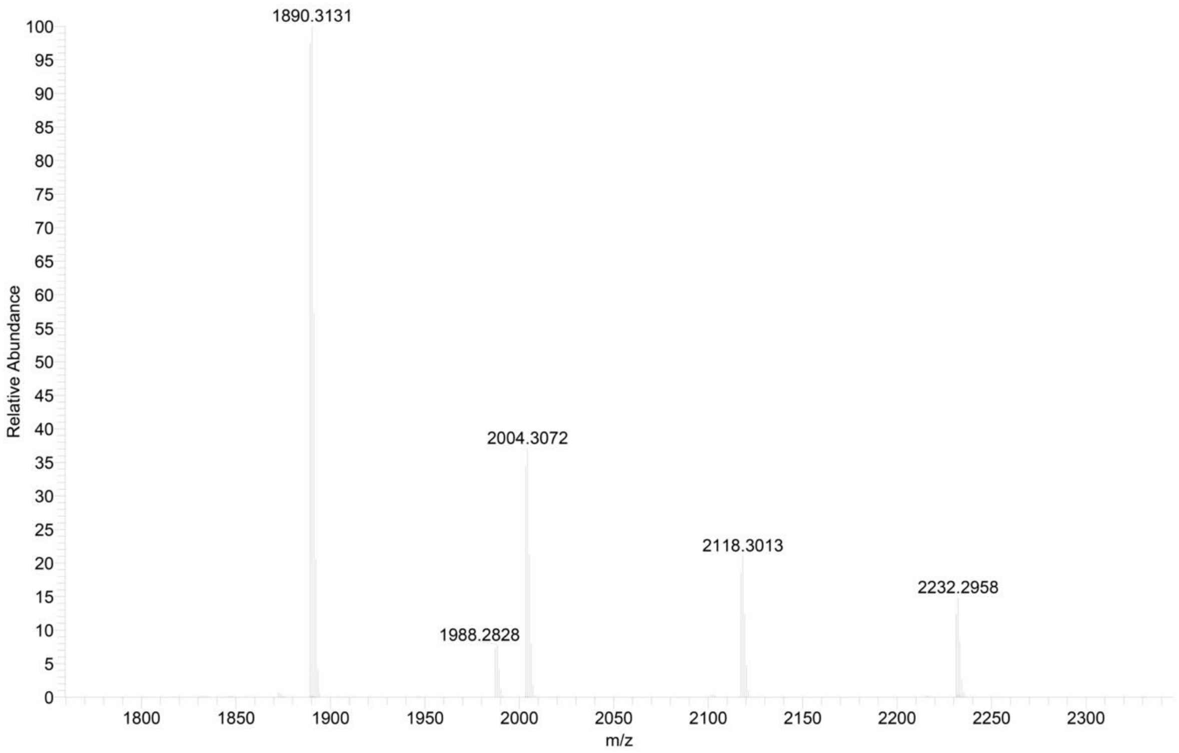
1890.3131
2004.3072
2118.3013
2232.2958
1988.2828
Relative Abundance
m/z

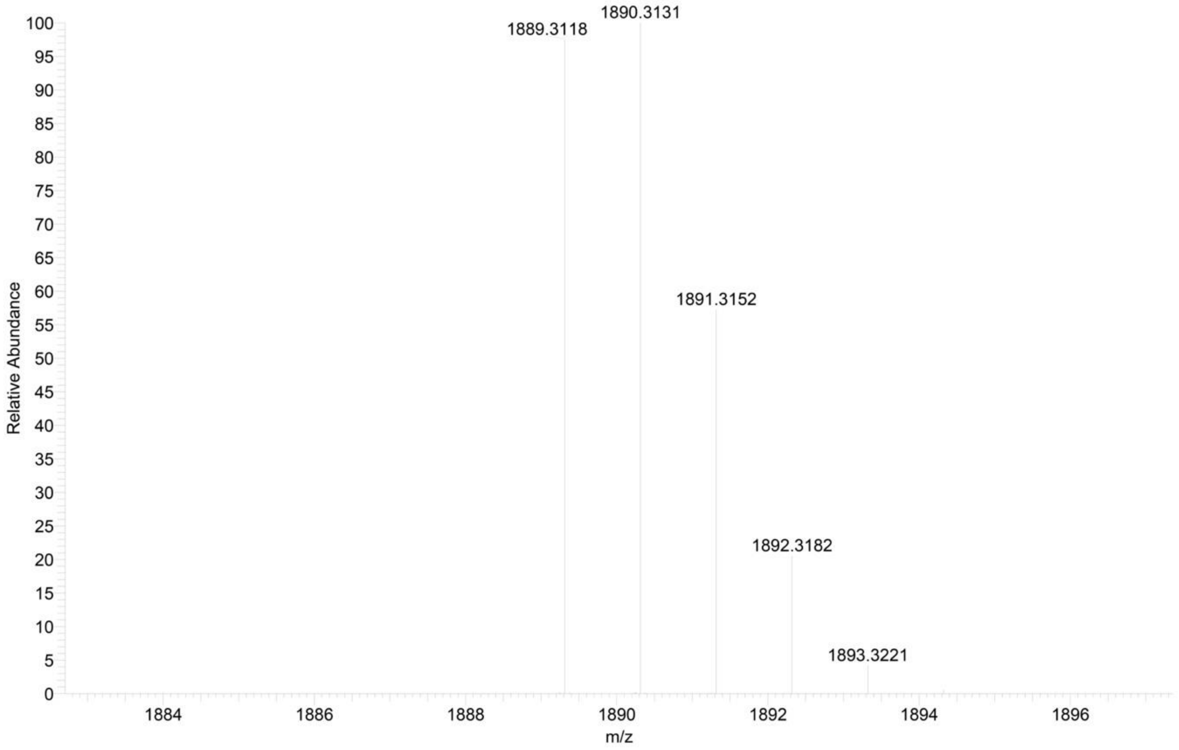
1890.3131
1889.3118
1891.3152
1892.3182
1893.3221
Relative Abundance
m/z

GN12 (GNWRKIVHKIKKAG-NH_2) was obtained from Rink Amide AM resin LL (400 mg, 0.26 mmol/g), the peptide was obtained as a white foamy solid after preparative RP-HPLC purification (252.9 mg, 36.9%). Analytical RP-HPLC: t_R = 1.24 min (100% A to 100% D in 3.5 min, λ= 214 nm). MS (ESI+): $C_{75}H_{128}N_{26}O_{15}$ calc./obs. 1633.01/1633.01 $[M]^+$.

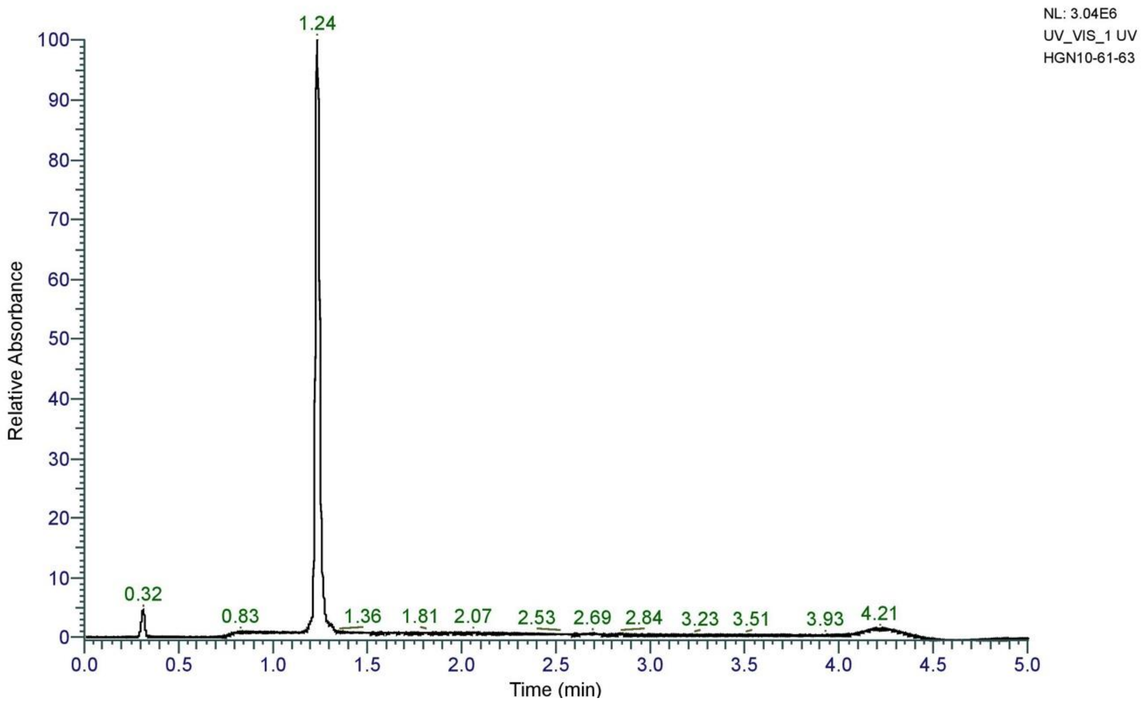

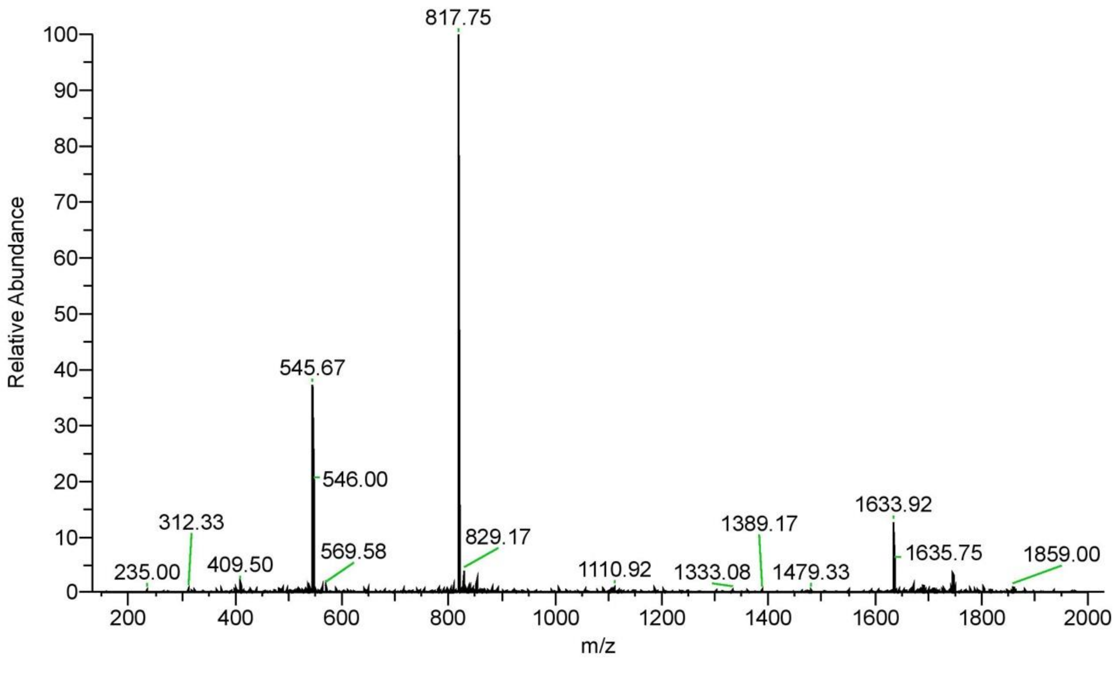

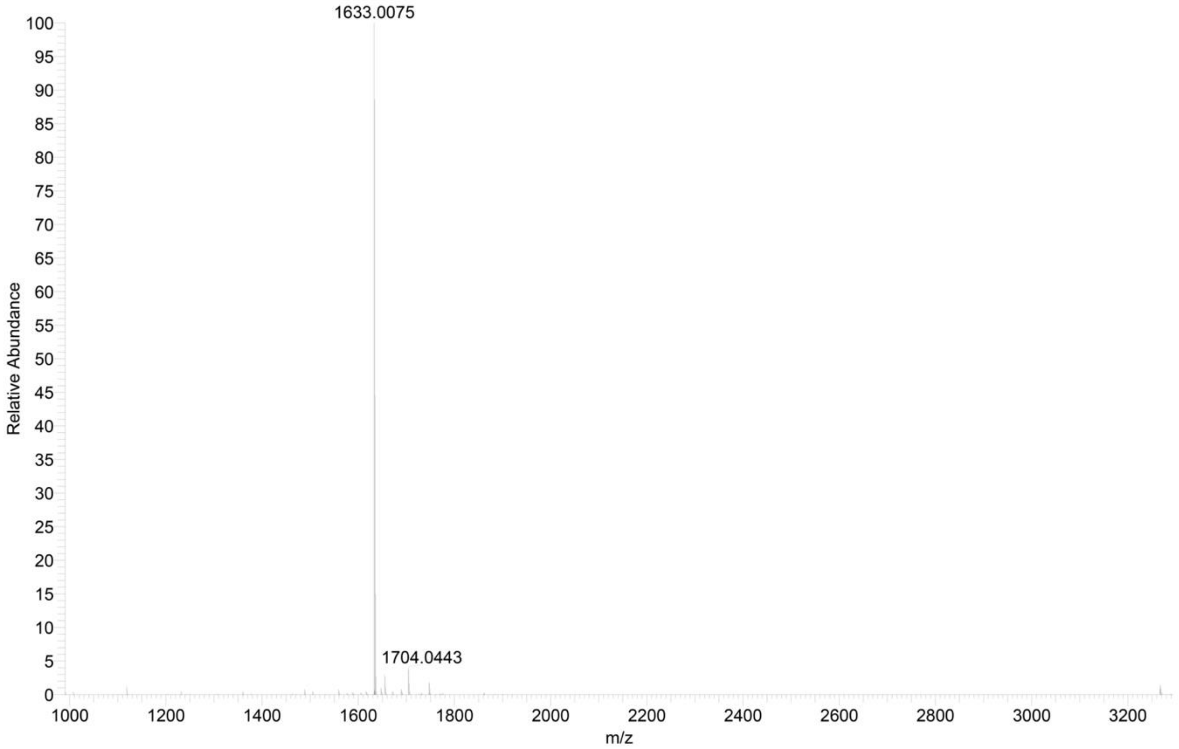
1633.0075
1704.0443
Relative Abundance
m/z

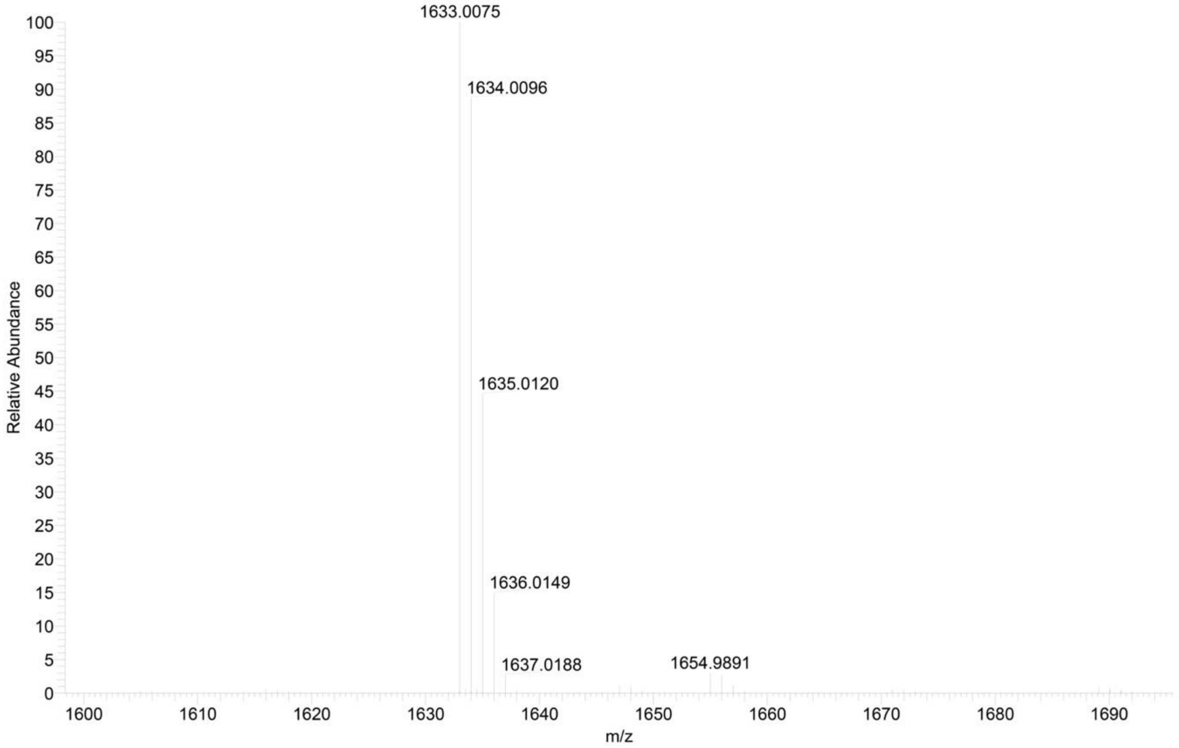
1633.0075
1634.0096
1635.0120
1636.0149
1637.0188
1654.9891
Relative Abundance
m/z

GN13 (AGRLQKVFKVIAK-NH_2) was obtained from Rink Amide AM resin LL (400 mg, 0.26 mmol/g), the peptide was obtained as a white foamy solid after preparative RP-HPLC purification (210.8 mg, 23.7%). Analytical RP-HPLC: t_R = 1.33 min (100% A to 100% D in 3.5 min, λ= 214 nm). MS (ESI+): $C_{68}H_{121}N_{21}O_{14}$ calc./obs. 1455.94/1455.94 $[M]^+$.

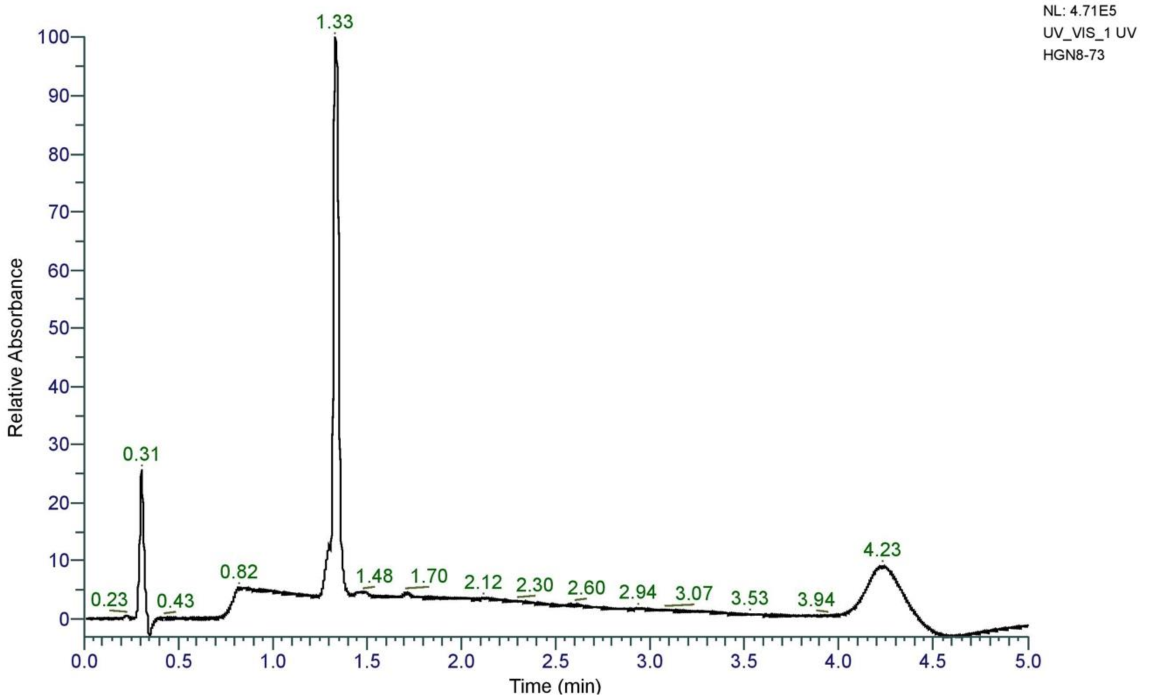

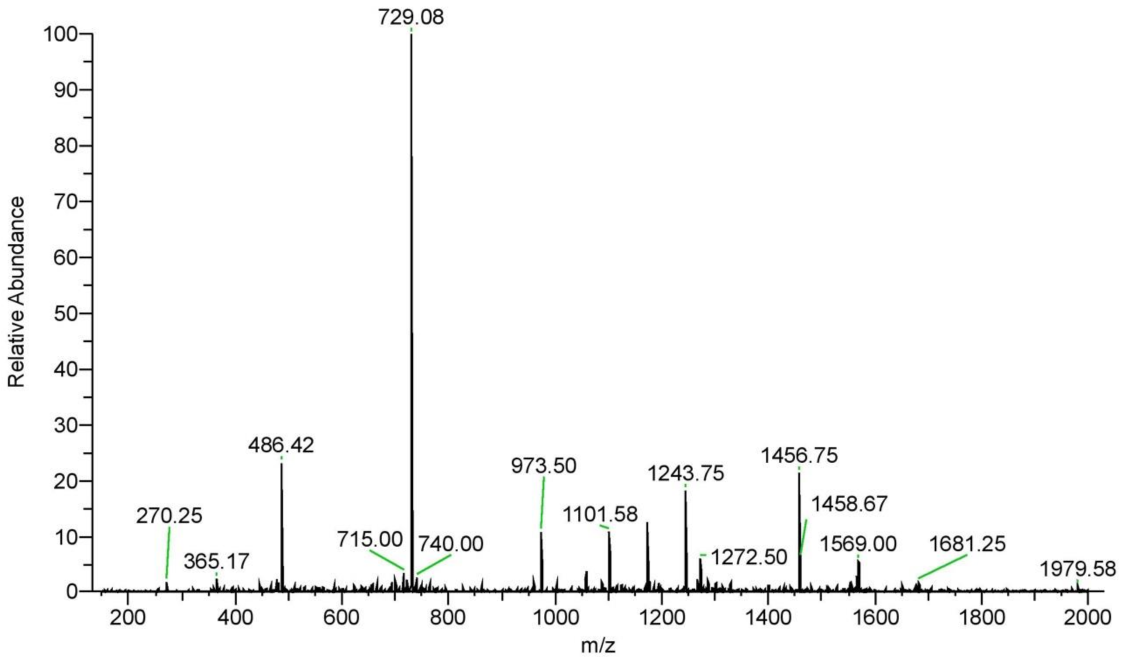

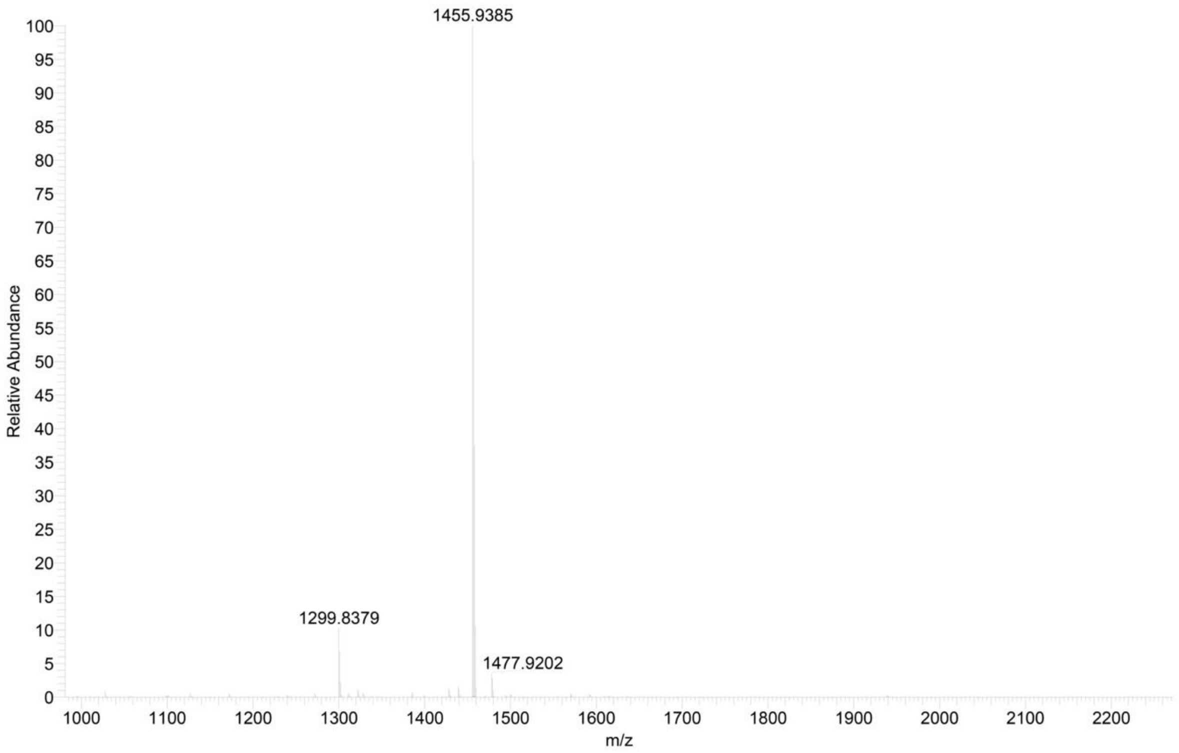
1455.9385
1299.8379
1477.9202
Relative Abundance
m/z

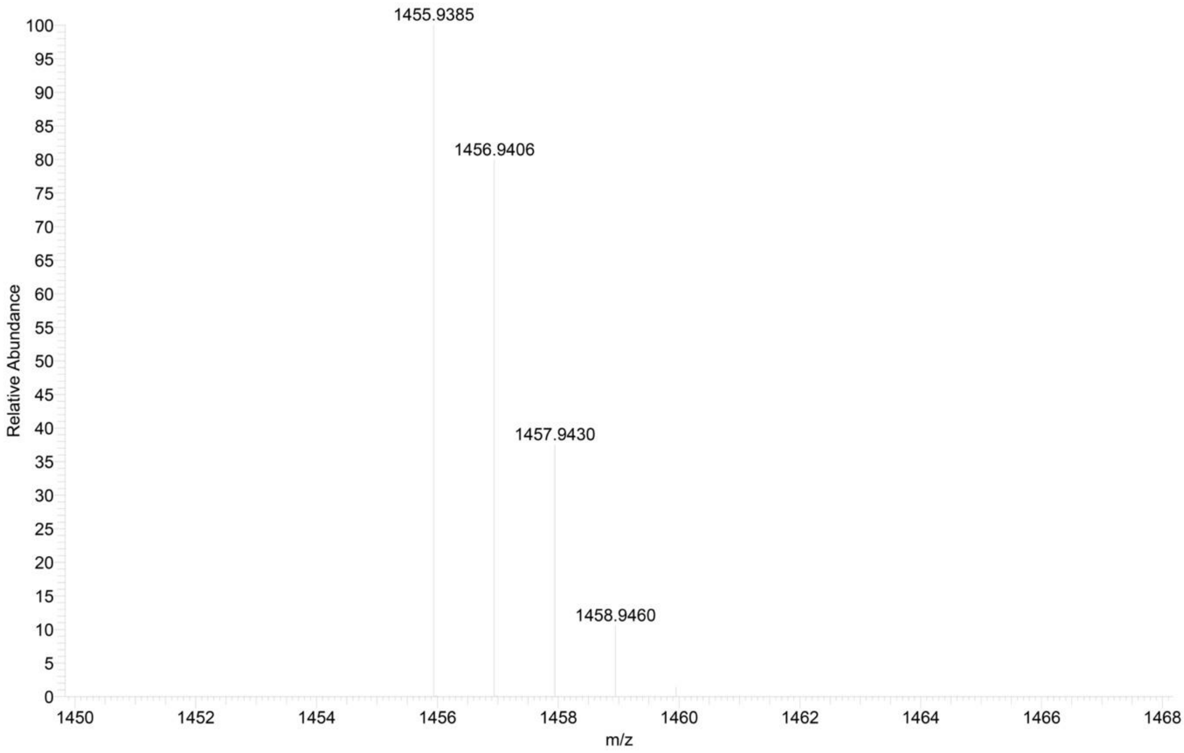
1455.9385
1456.9406
1457.9430
1458.9460
Relative Abundance
m/z

GN14 (IHKLAKLAKNVL-NH_2) was obtained from Rink Amide AM resin LL (400 mg, 0.26 mmol/g), the peptide was obtained as a white foamy solid after preparative RP-HPLC purification (199.4 mg, 18.0%). Analytical RP-HPLC: t_R = 1.32 min (100% A to 100% D in 3.5 min, λ= 214 nm). MS (ESI+): $C_{63}H_{115}N_{19}O_{13}$ calc./obs. 1345.89/1345.89 $[M]^+$.

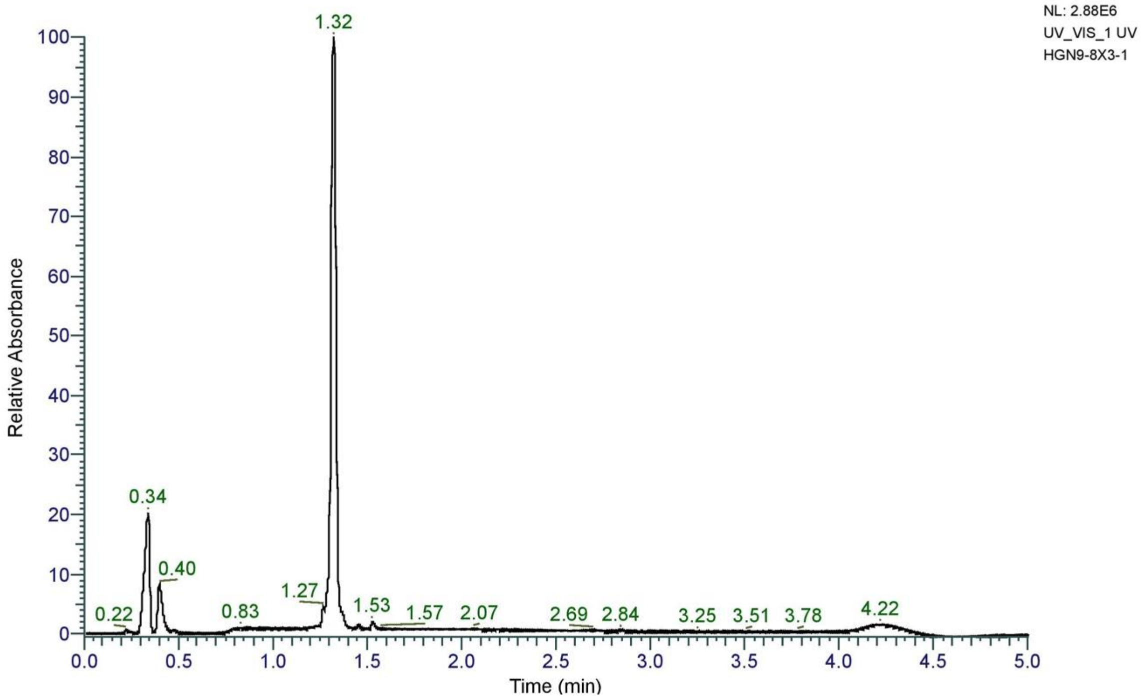

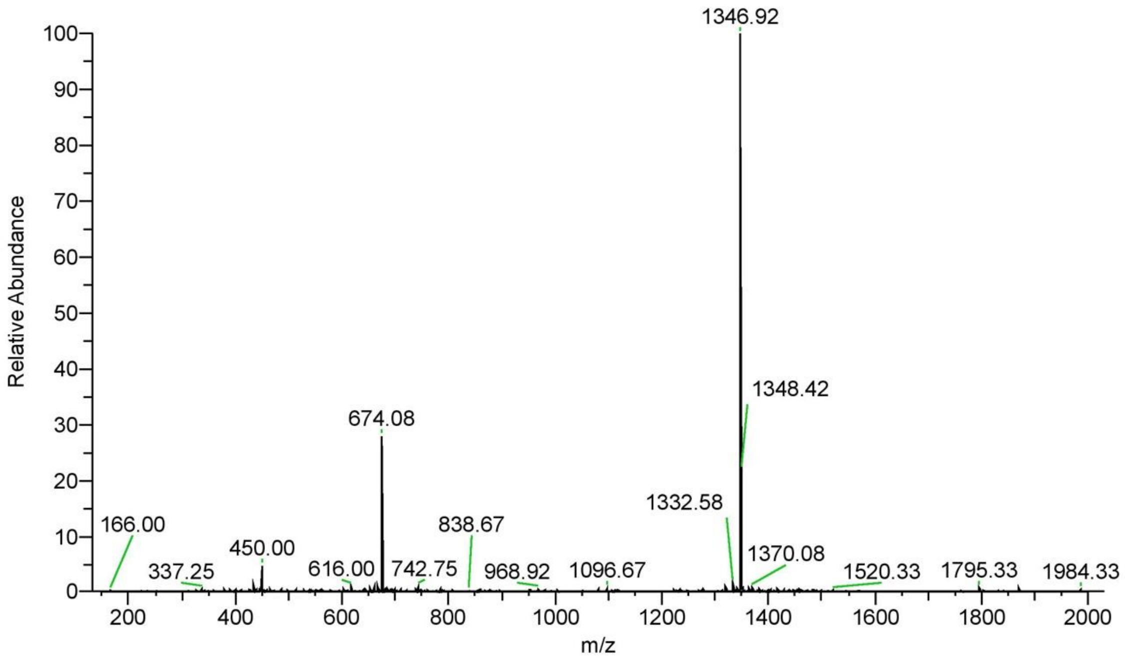

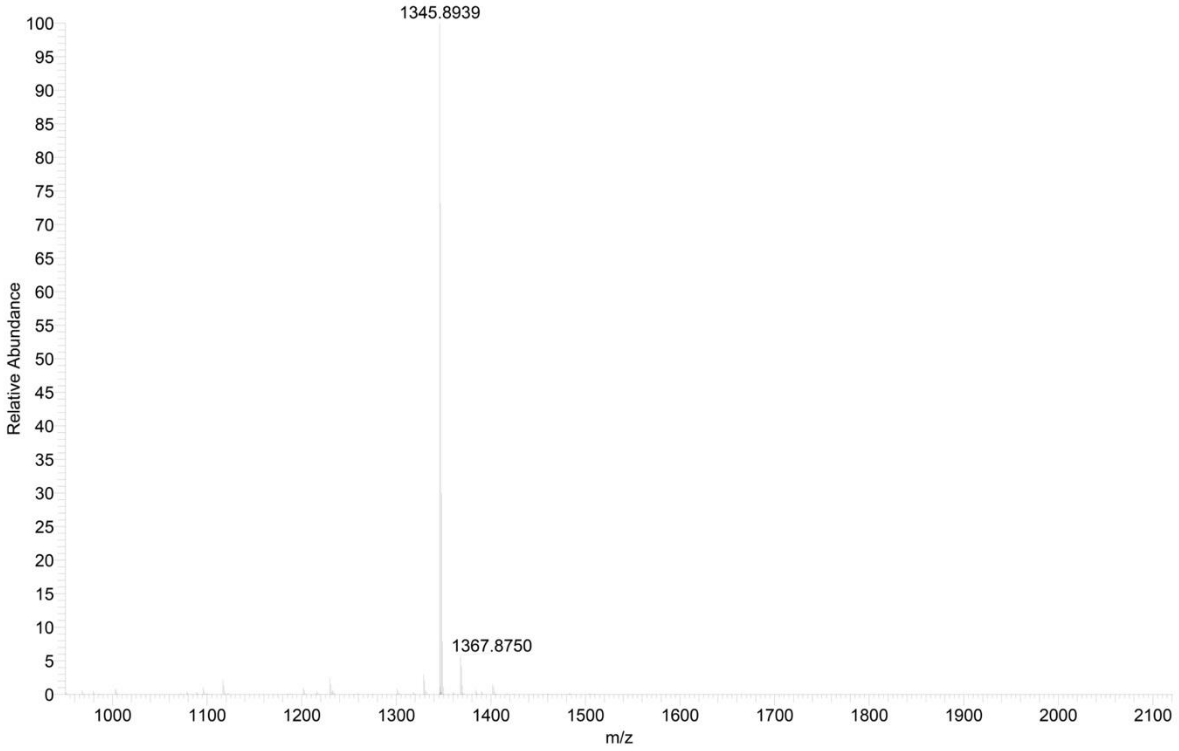
1345.8939
1367.8750
Relative Abundance
m/z

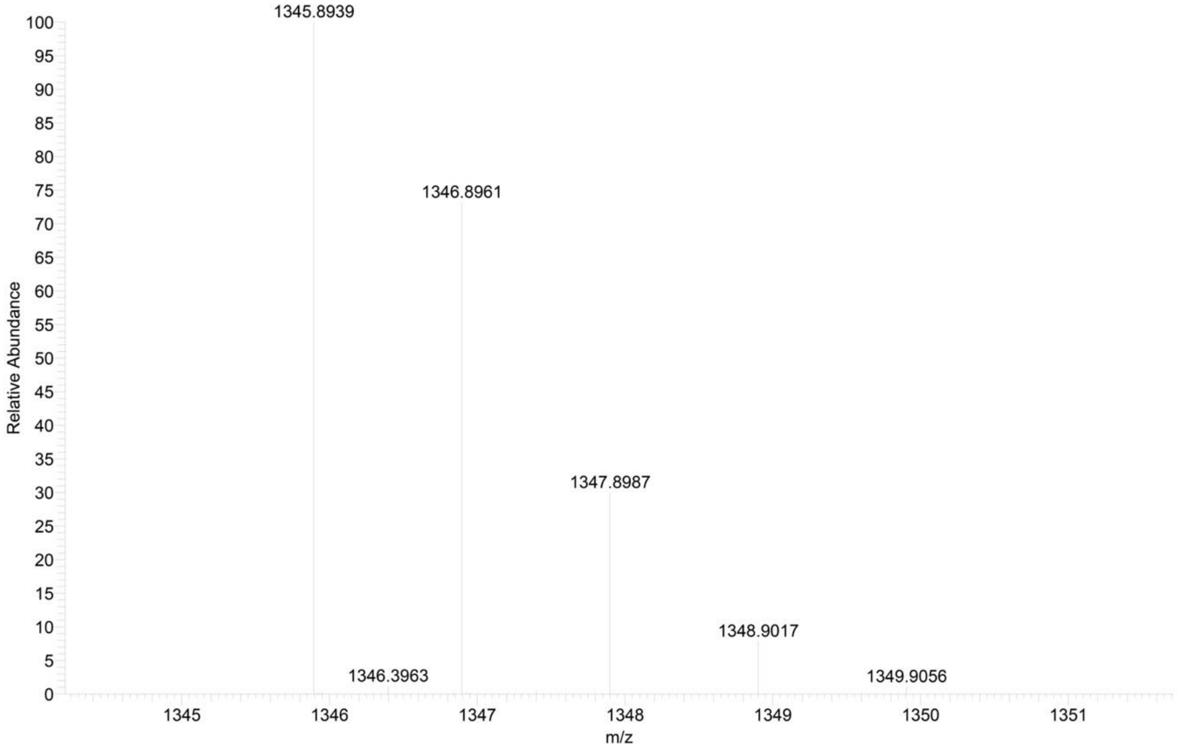
1345.8939
1346.8961
1347.8987
1348.9017
1346.3963
1349.9056
Relative Abundance
m/z

GP1 (FLKAVKKLIPSLF-NH_2) was obtained from Rink Amide AM resin LL (400 mg, 0.26 mmol/g), the peptide was obtained as a white foamy solid after preparative RP-HPLC purification (203.7 mg, 6.9%). Analytical RP-HPLC: t_R = 1.56 min (100% A to 100% D in 3.5 min, λ= 214 nm). MS (ESI+): $C_{76}H_{127}N_{17}O_{14}$ calc./obs. 1501.97/1501.97 $[M]^+$.

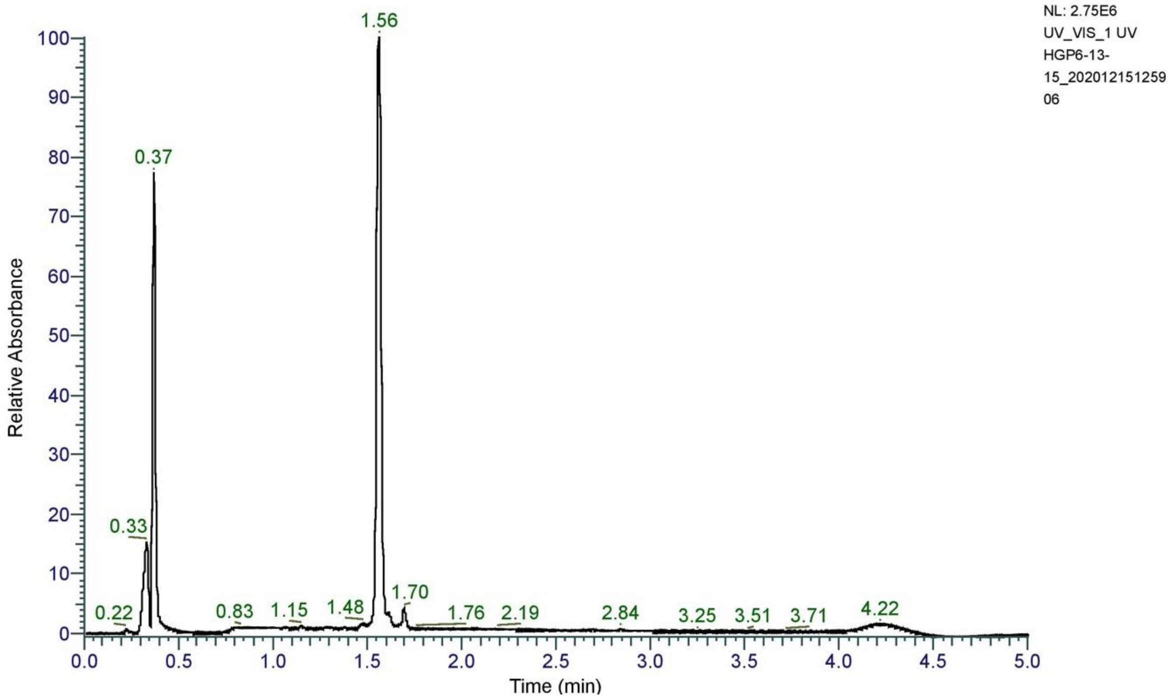

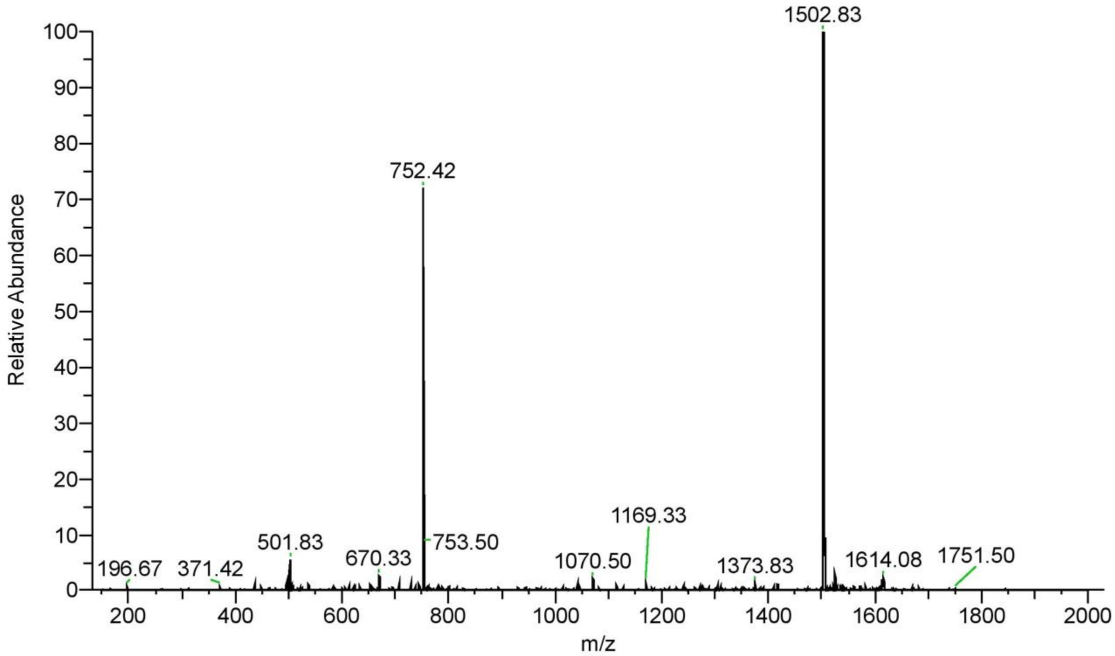

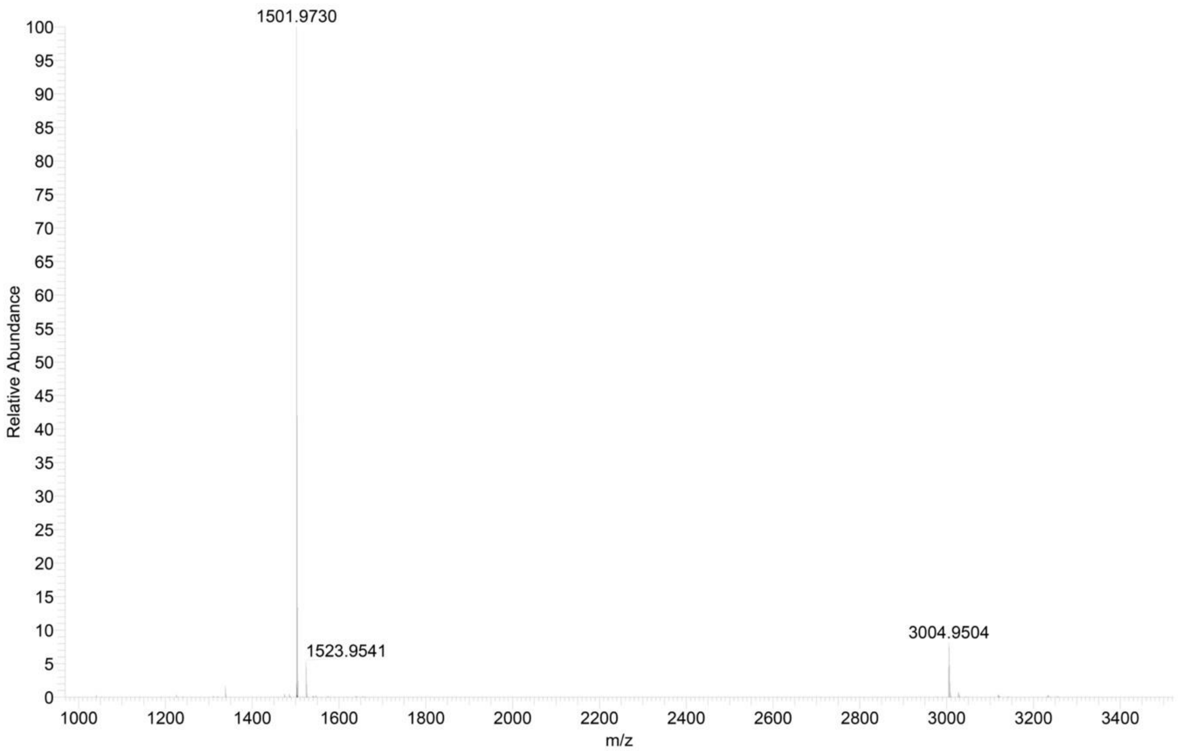
1501.9730
1523.9541
3004.9504
Relative Abundance
m/z

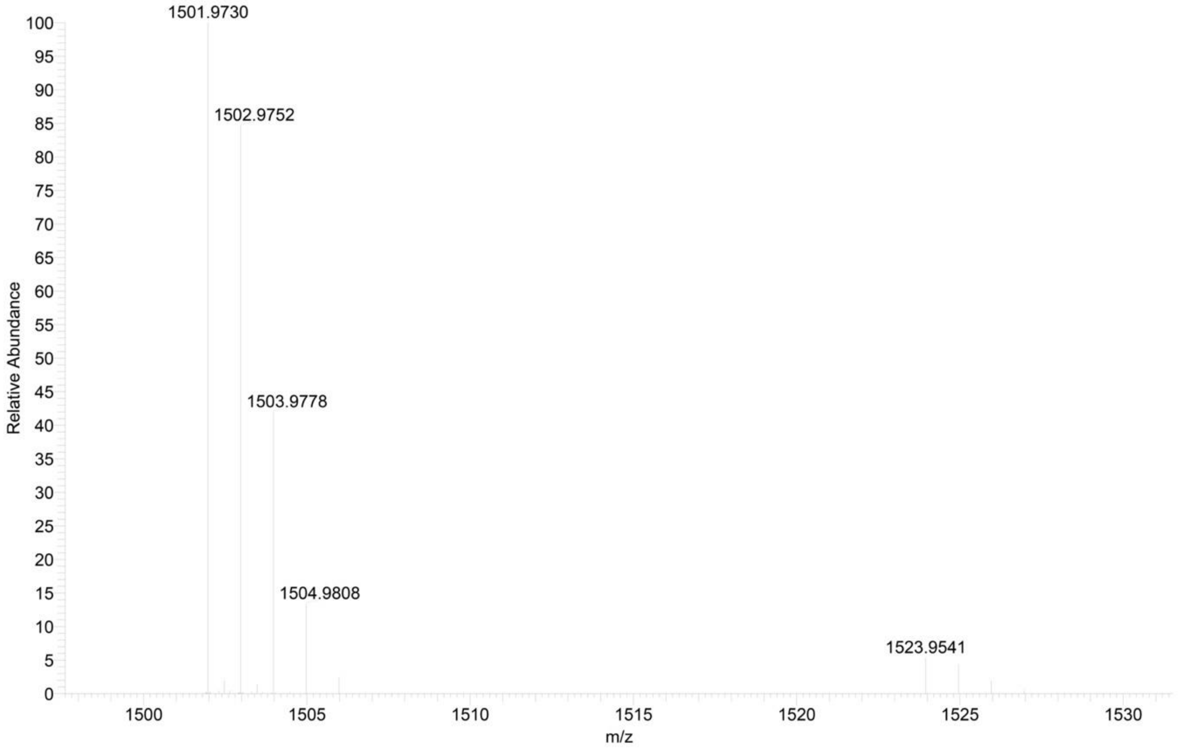
1501.9730
1502.9752
1503.9778
1504.9808
1523.9541
Relative Abundance
m/z

GP2 (RWRWPILGRILR-NH_2) was obtained from Rink Amide AM resin LL (400 mg, 0.26 mmol/g), the peptide was obtained as a white foamy solid after preparative RP-HPLC purification (227.9 mg, 18.3%). Analytical RP-HPLC: t_R = 1.54 min (100% A to 100% D in 3.5 min, λ= 214 nm). MS (ESI+): $C_{77}H_{125}N_{27}O_{12}$ calc./obs. 1620.00/1620.00 $[M]^+$.

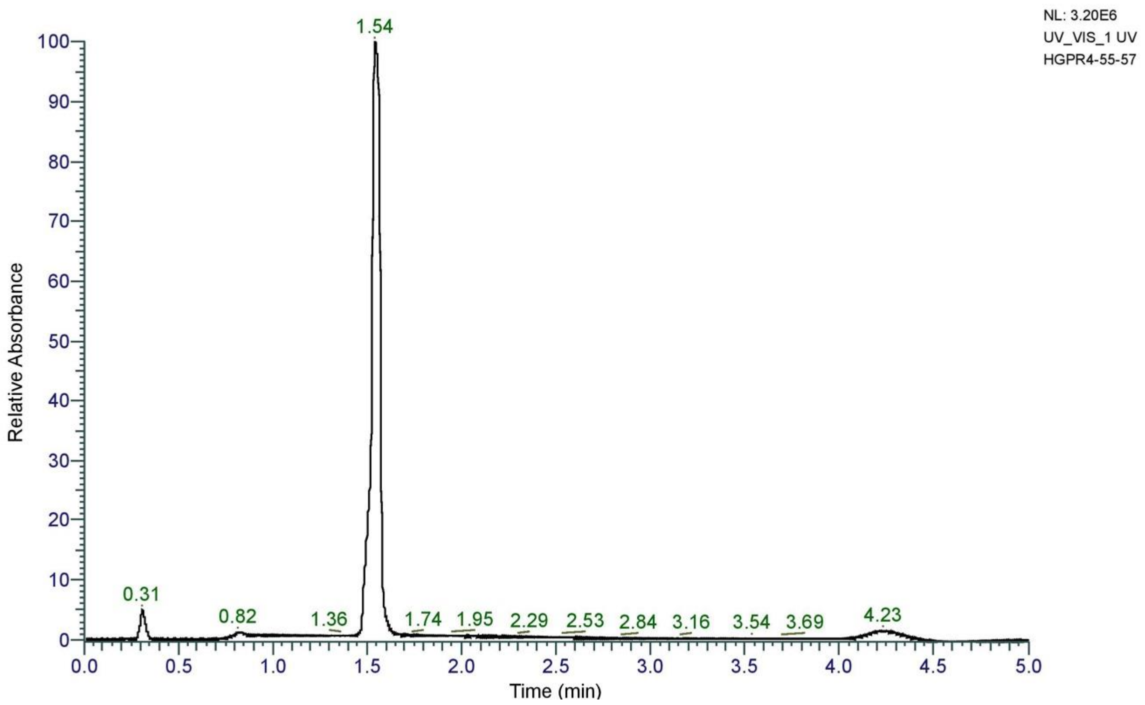

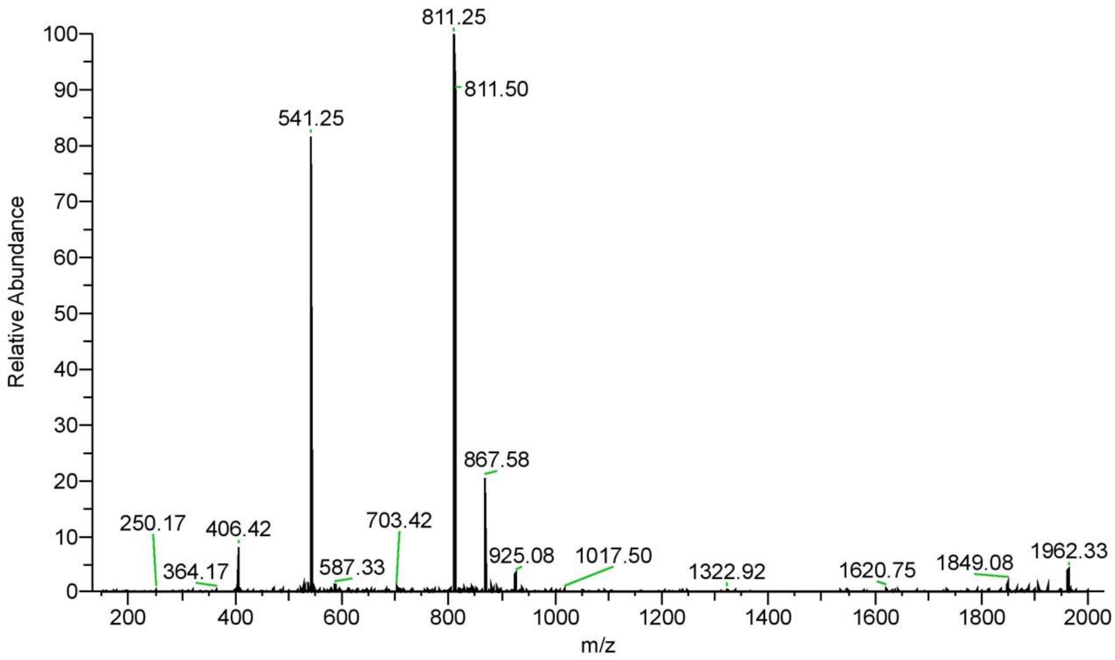

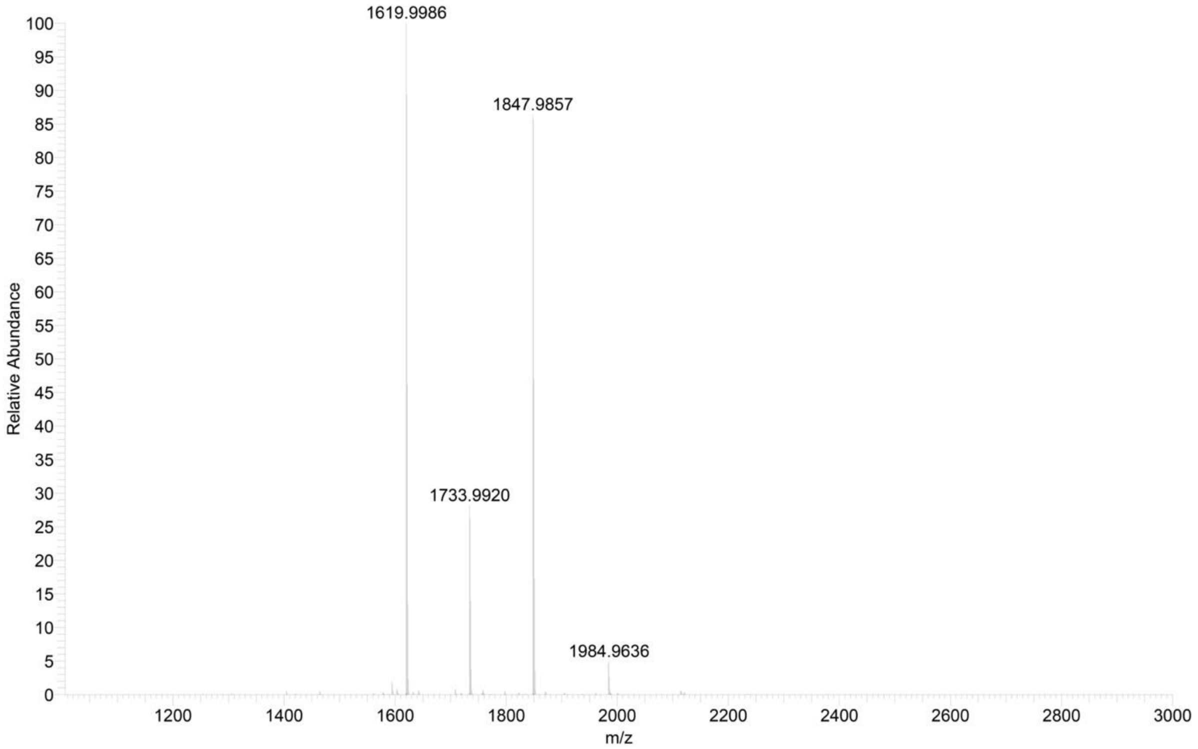
1619.9986
1847.9857
1733.9920
1984.9636
Relative Abundance
m/z

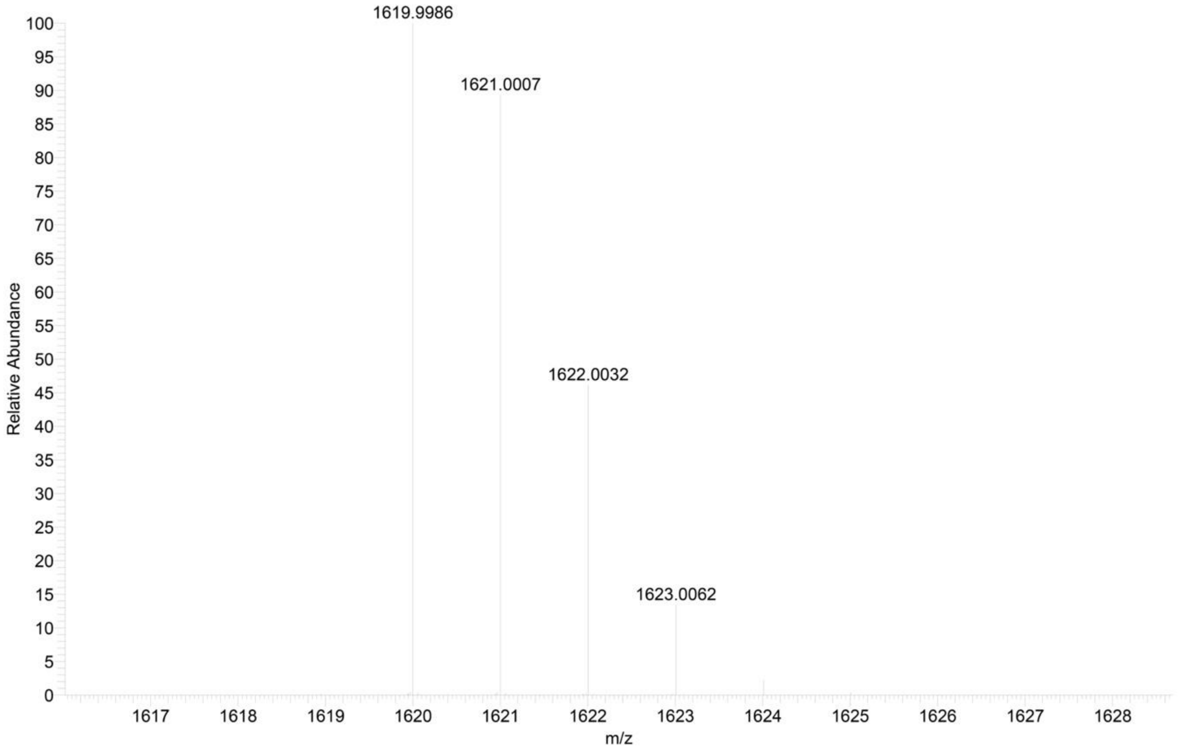
1619.9986
1621.0007
1622.0032
1623.0062
Relative Abundance
m/z

GP3 (FLHSIGKAIGRLLR-NH_2 was obtained from Rink Amide AM resin LL (400 mg, 0.26 mmol/g), the peptide was obtained as a white foamy solid after preparative RP-HPLC purification (223.6 mg, 9.3%). Analytical RP-HPLC: t_R = 1.51 min (100% A to 100% D in 3.5 min, λ= 214 nm). MS (ESI+): $C_{73}H_{126}N_{24}O_{15}$ calc./obs. 1578.98/1578.99 $[M]^+$.

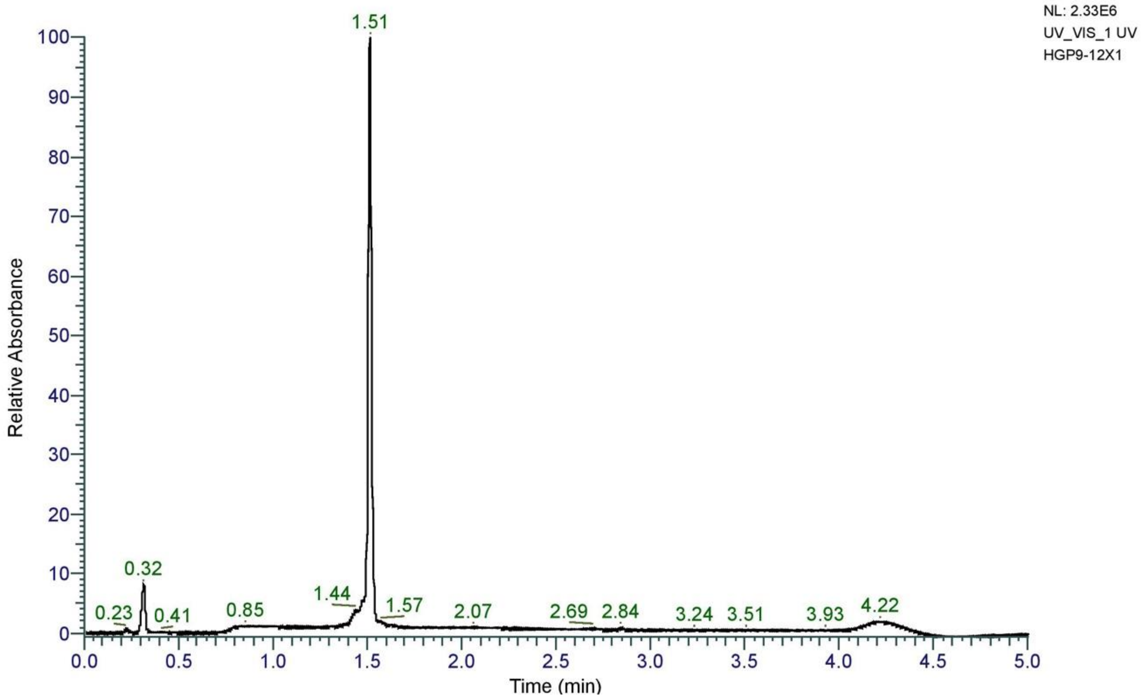

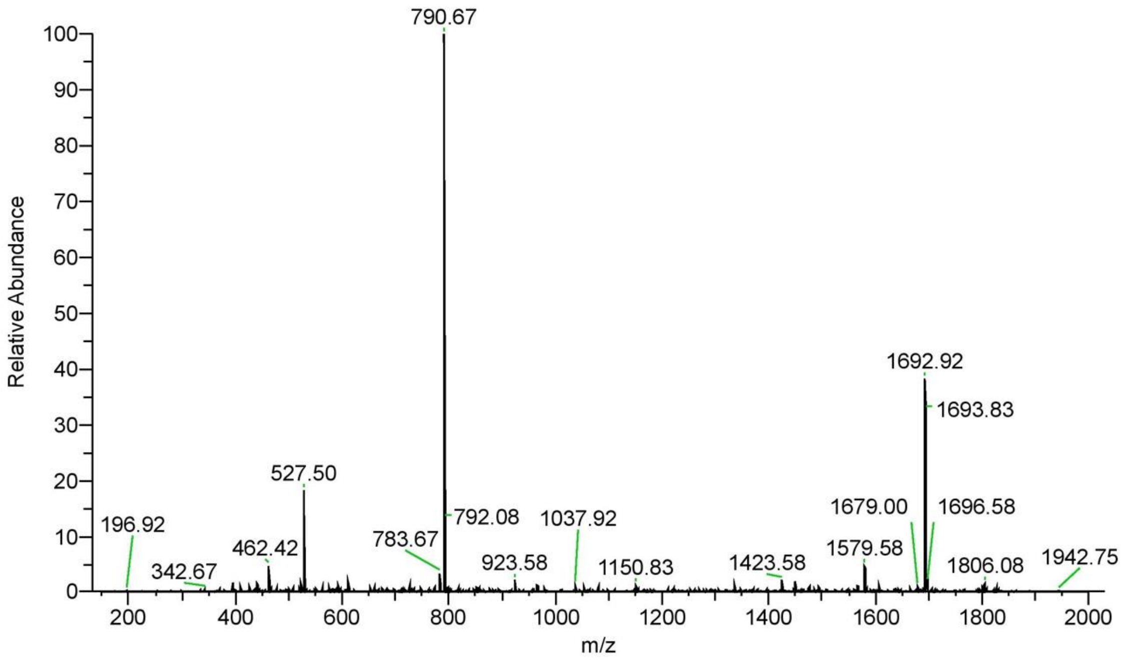

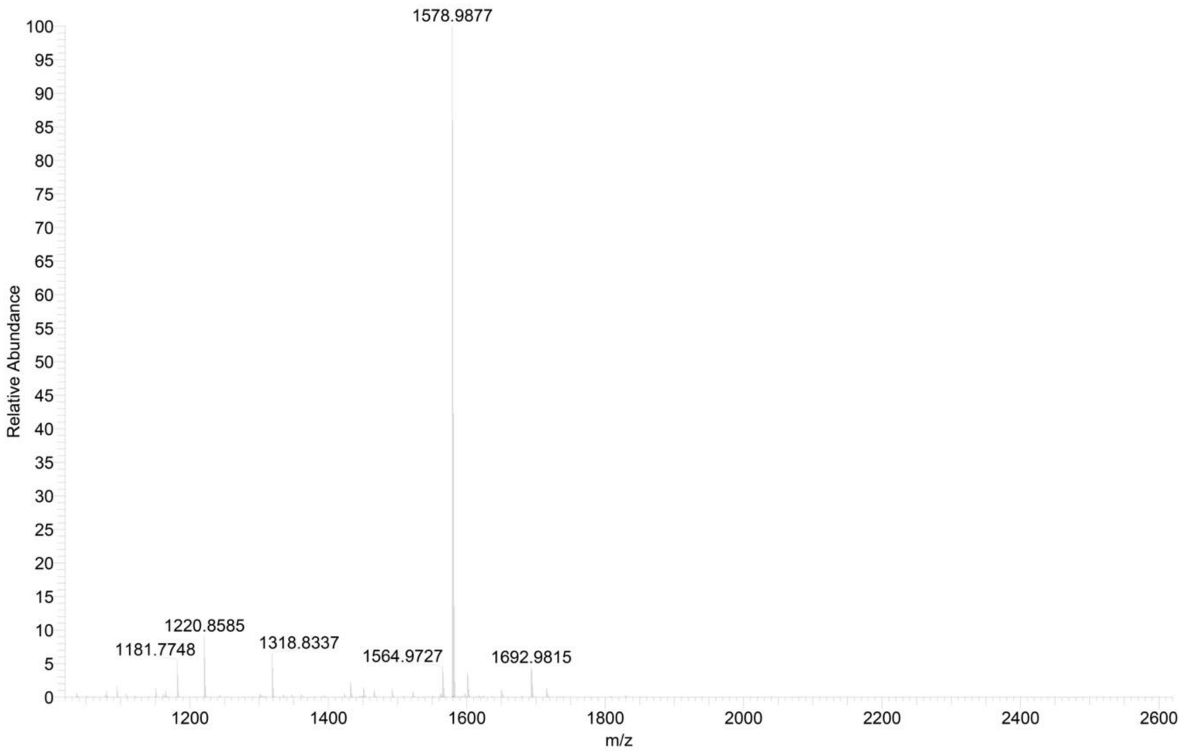
1578.9877
1220.8585
1181.7748
1318.8337
1564.9727
1692.9815
Relative Abundance
m/z

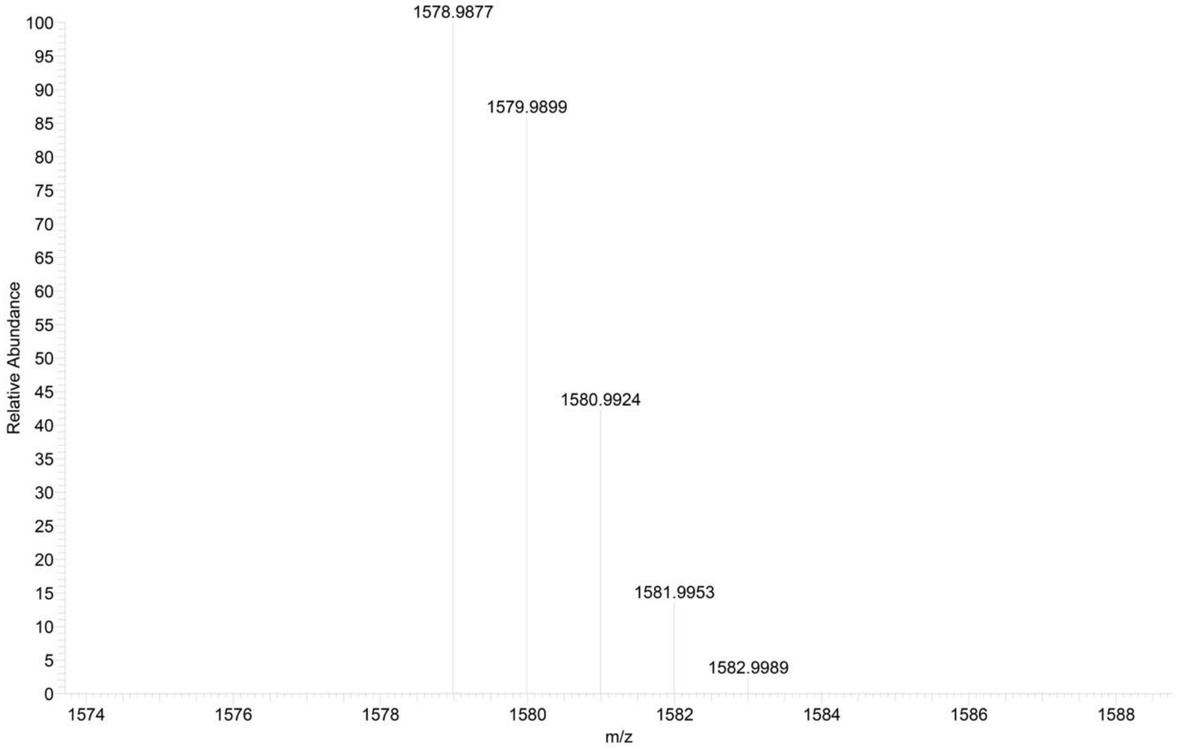
1578.9877
1579.9899
1580.9924
1581.9953
1582.9989
Relative Abundance
m/z

GP4 (GIGAVLNVAKKLL-NH_2) was obtained from Rink Amide AM resin LL (400 mg, 0.26 mmol/g), the peptide was obtained as a white foamy solid after preparative RP-HPLC purification (170.2 mg, 3.3%). Analytical RP-HPLC: t_R = 1.70 min (100% A to 100% D in 3.5 min, λ= 214 nm). MS (ESI+): $C_{60}H_{111}N_{17}O_{14}$ calc./obs. 1293.85/1293.85 $[M]^+$.

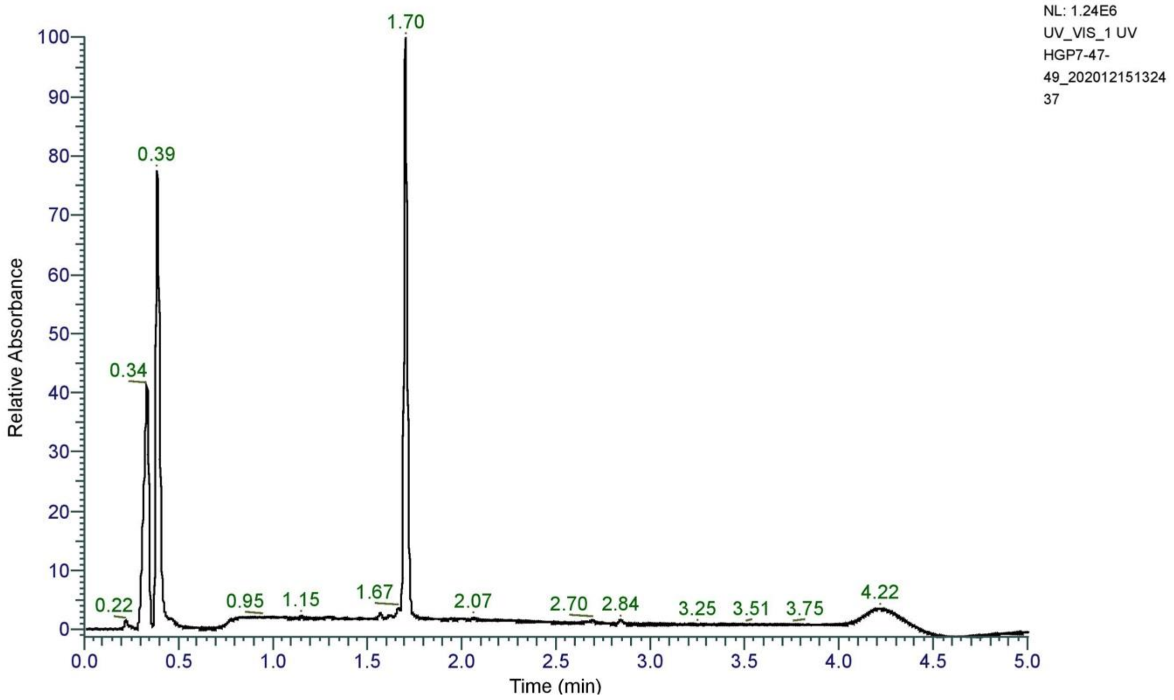

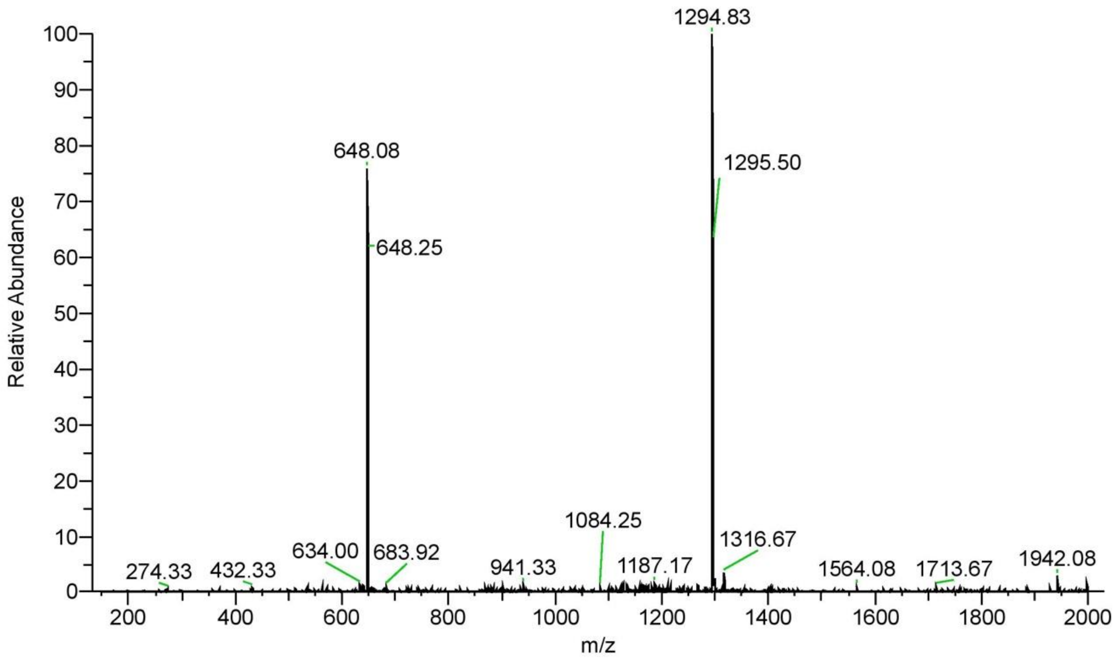

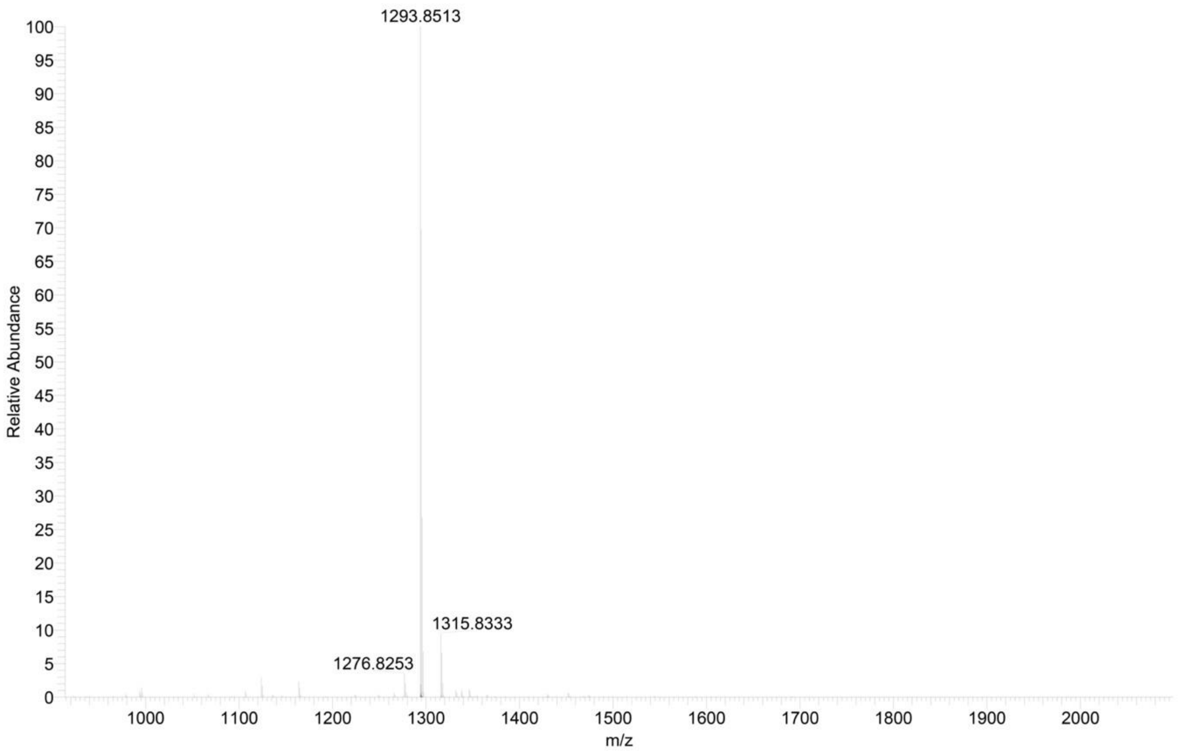
1293.8513
1315.8333
1276.8253
Relative Abundance
m/z

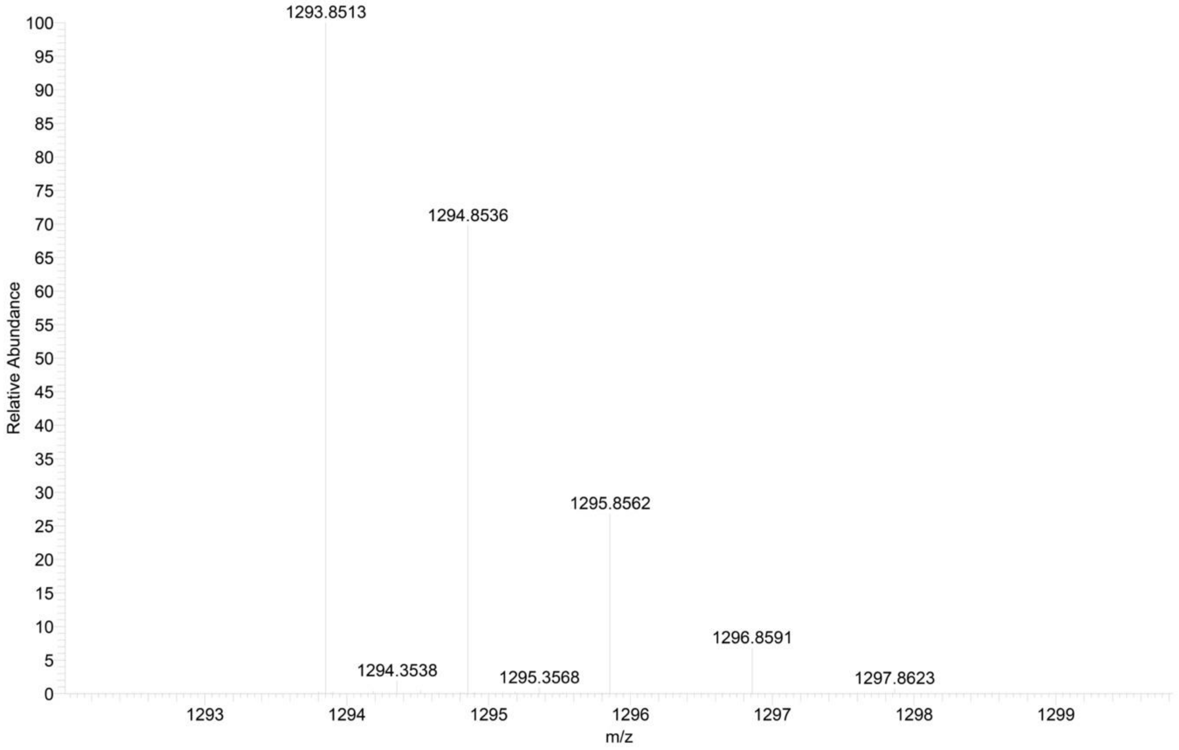
1293.8513
1294.8536
1295.8562
1296.8591
1294.3538
1295.3568
1297.8623
Relative Abundance
m/z

GP5 (KVARFLKKFFR-NH_2) was obtained from Rink Amide AM resin LL (400 mg, 0.26 mmol/g), the peptide was obtained as a white foamy solid after preparative RP-HPLC purification (220.8 mg, 10.7%). Analytical RP-HPLC: t_R = 1.38 min (100% A to 100% D in 3.5 min, λ= 214 nm). MS (ESI+): $C_{71}H_{115}N_{21}O_{11}$ calc./obs. 1437.91/1437.91 $[M]^+$.

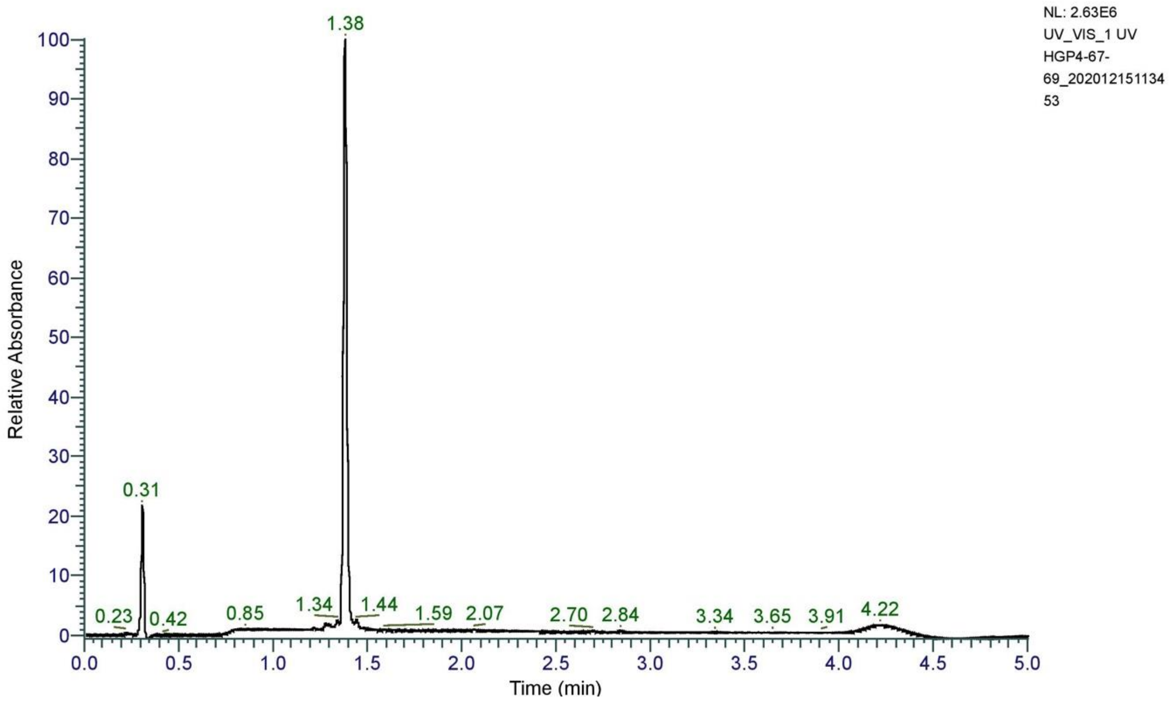

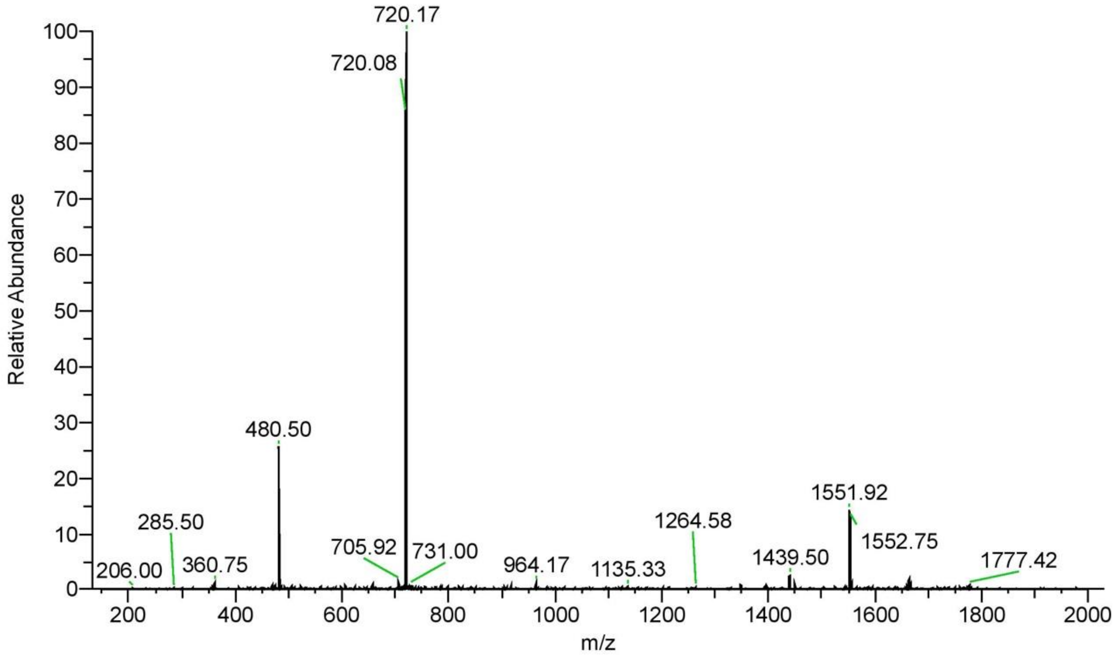

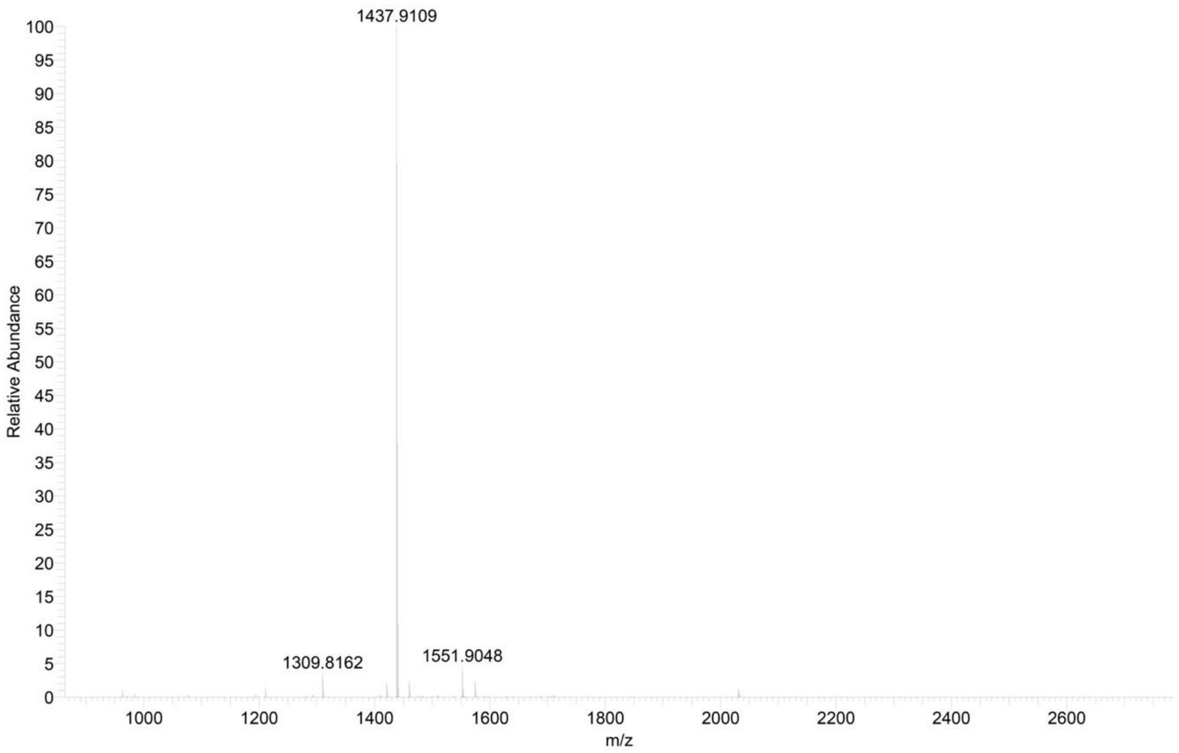
1437.9109
1309.8162
1551.9048
Relative Abundance
m/z

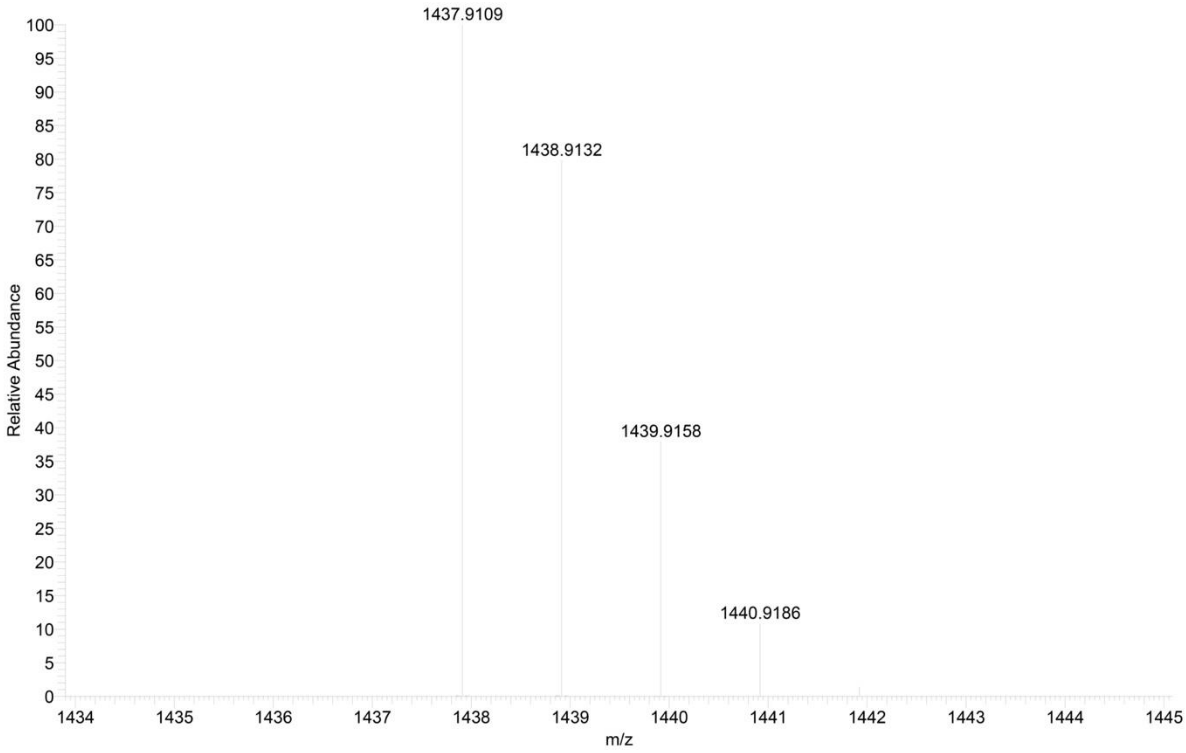
1437.9109
1438.9132
1439.9158
1440.9186
Relative Abundance
m/z

GP6 (LKKLWKRIIKVGR-NH_2) was obtained from Rink Amide AM resin LL (400 mg, 0.26 mmol/g), the peptide was obtained as a white foamy solid after preparative RP-HPLC purification (253.3 mg, 20.3%). Analytical RP-HPLC: t_R = 1.31 min (100% A to 100% D in 3.5 min, λ= 214 nm). MS (ESI+): $C_{78}H_{141}N_{25}O_{13}$ calc./obs. 1636.11/1636.11 $[M]^+$.

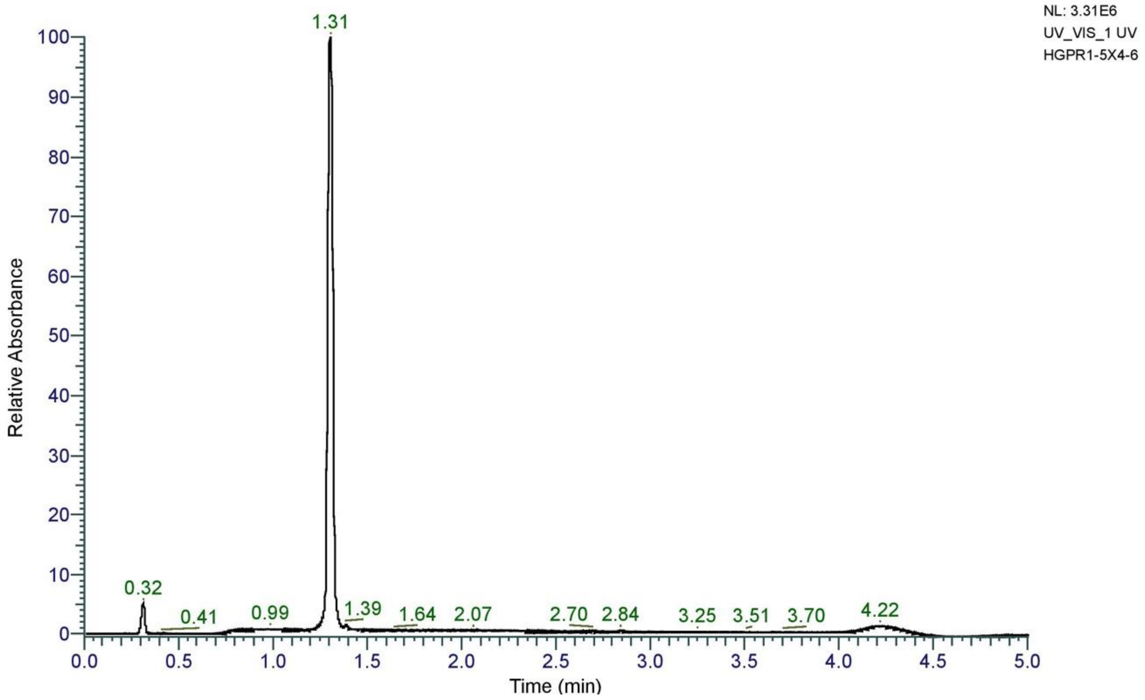

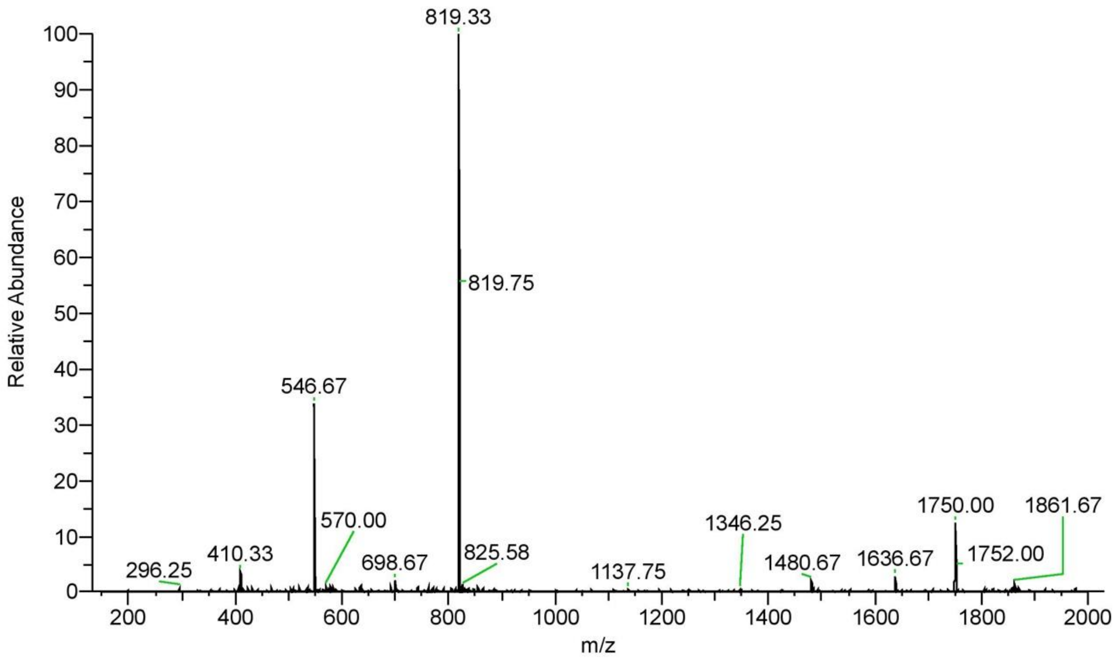

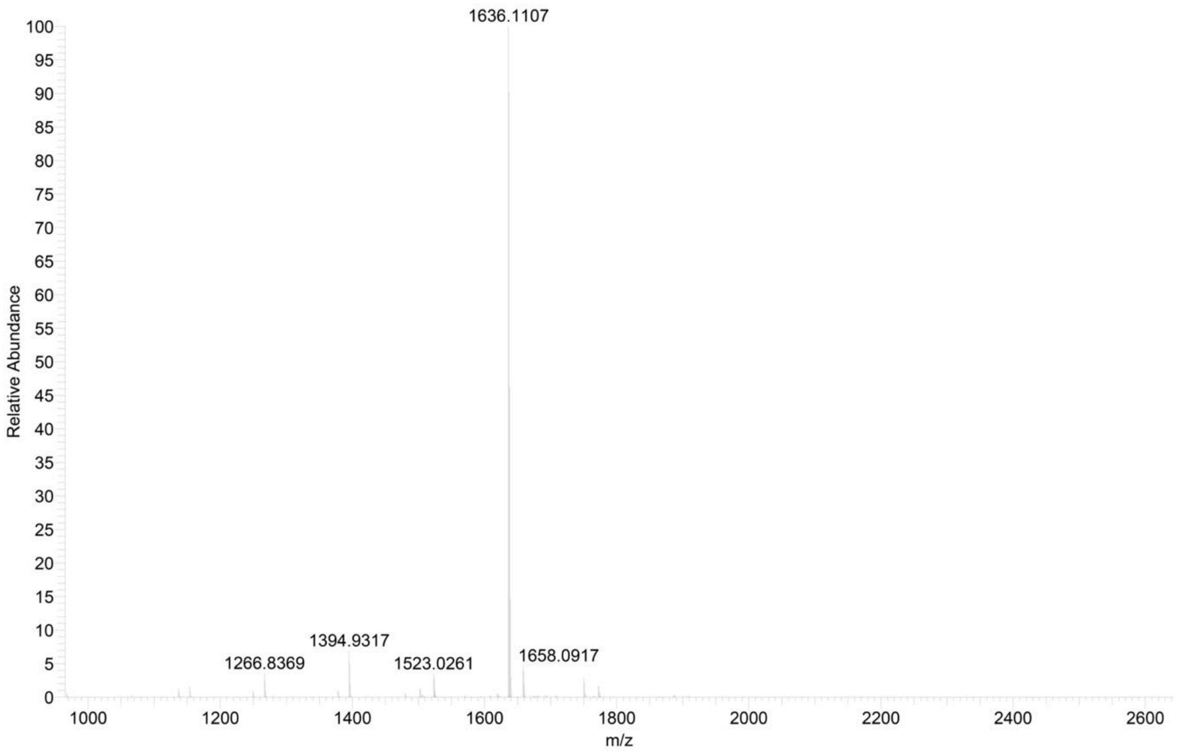
1636.1107
1394.9317
1266.8369
1523.0261
1658.0917
Relative Abundance
m/z

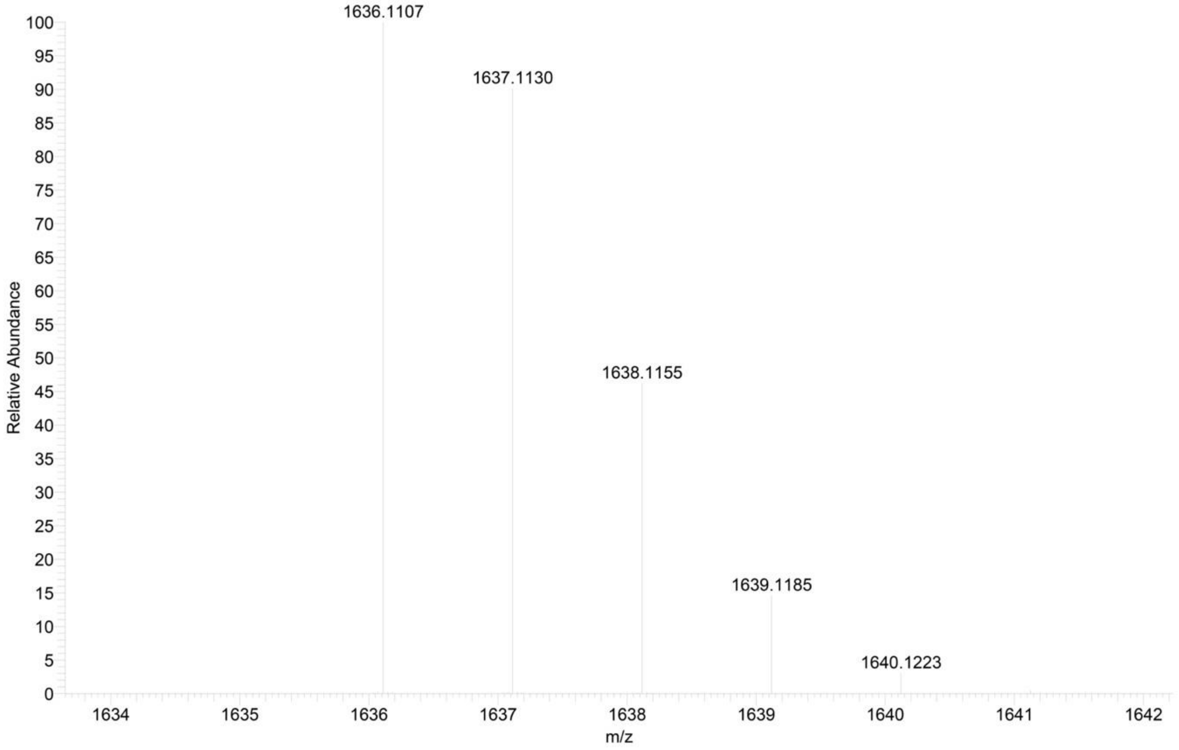
1636.1107
1637.1130
1638.1155
1639.1185
1640.1223
Relative Abundance
m/z

GP7 (ARKWRKFLKKI-NH_2) was obtained from Rink Amide AM resin LL (400 mg, 0.26 mmol/g), the peptide was obtained as a white foamy solid after preparative RP-HPLC purification (236.2 mg, 16.9%). Analytical RP-HPLC: t_R = 1.28 min (100% A to 100% D in 3.5 min, λ= 214 nm). MS (ESI+): $C_{71}H_{121}N_{23}O_{11}$ calc./obs. 1471.96/1471.96 $[M]^+$.

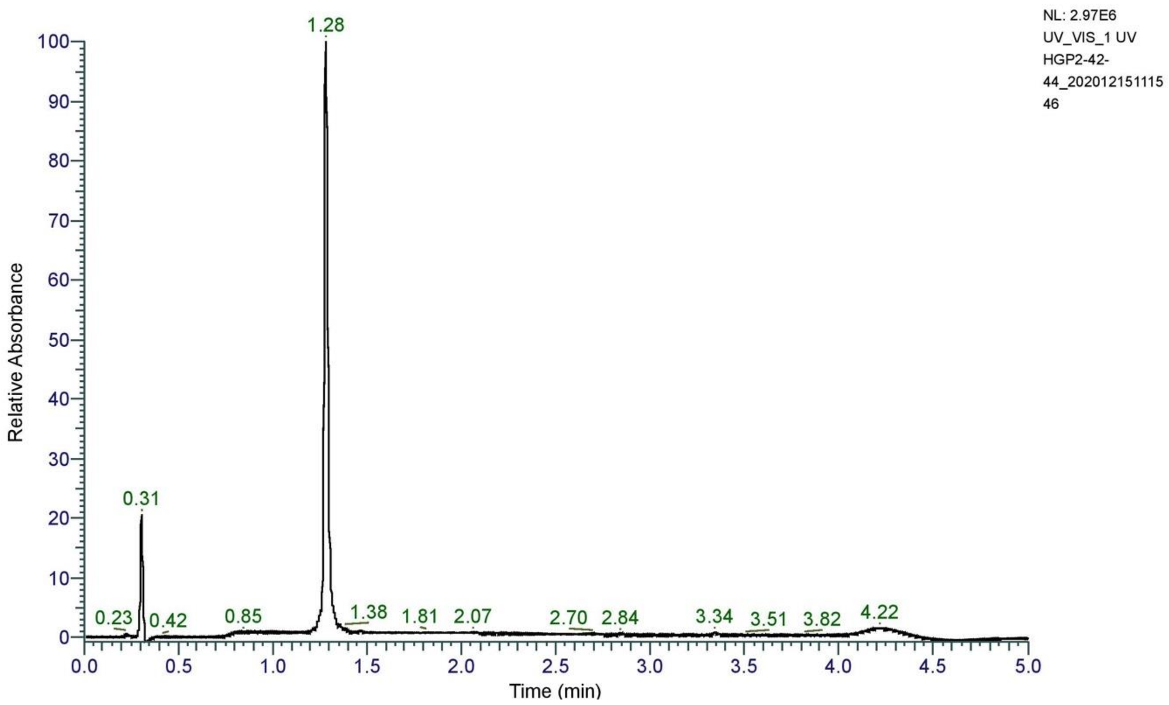

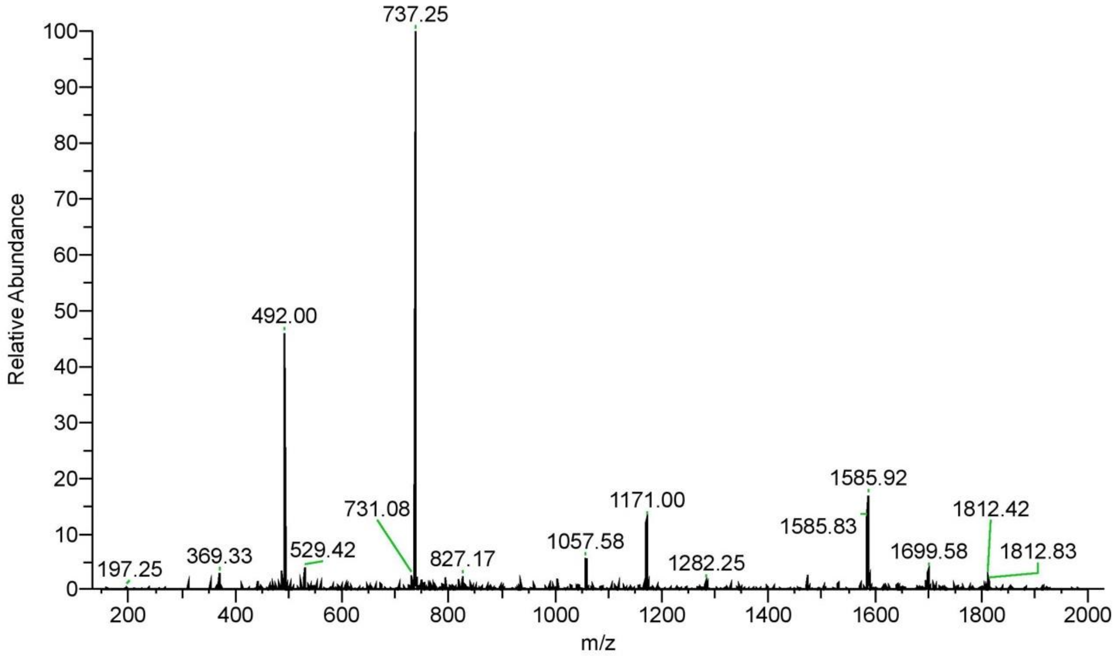

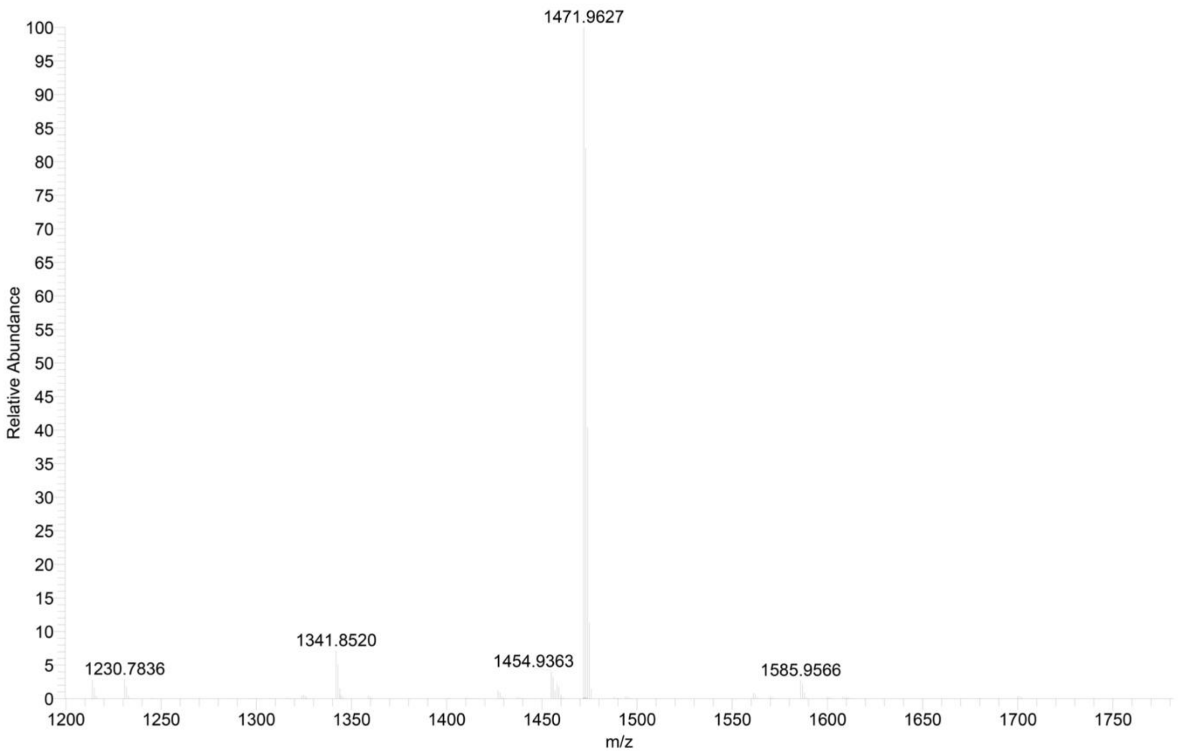
1471.9627
1341.8520
1454.9363
1230.7836
1585.9566
Relative Abundance
m/z

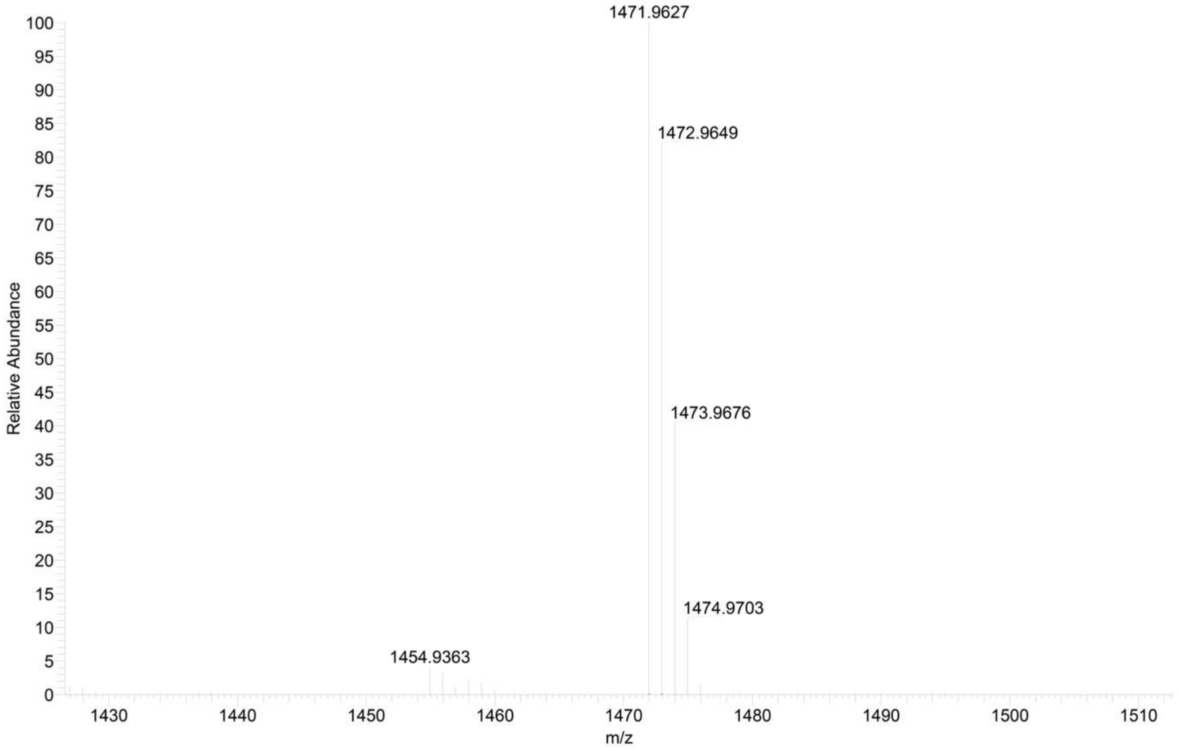
1471.9627
1472.9649
1473.9676
1474.9703
1454.9363
Relative Abundance
m/z

GP8 (GRIKRIRKIIHKY-NH_2) was obtained from Rink Amide AM resin LL (400 mg, 0.26 mmol/g), the peptide was obtained as a white foamy solid after preparative RP-HPLC purification (269.6 mg, 20.6%). Analytical RP-HPLC: t_R = 1.22 min (100% A to 100% D in 3.5 min, λ= 214 nm). MS (ESI+): $C_{77}H_{138}N_{28}O_{14}$ calc./obs. 1679.09/1679.10 $[M]^+$

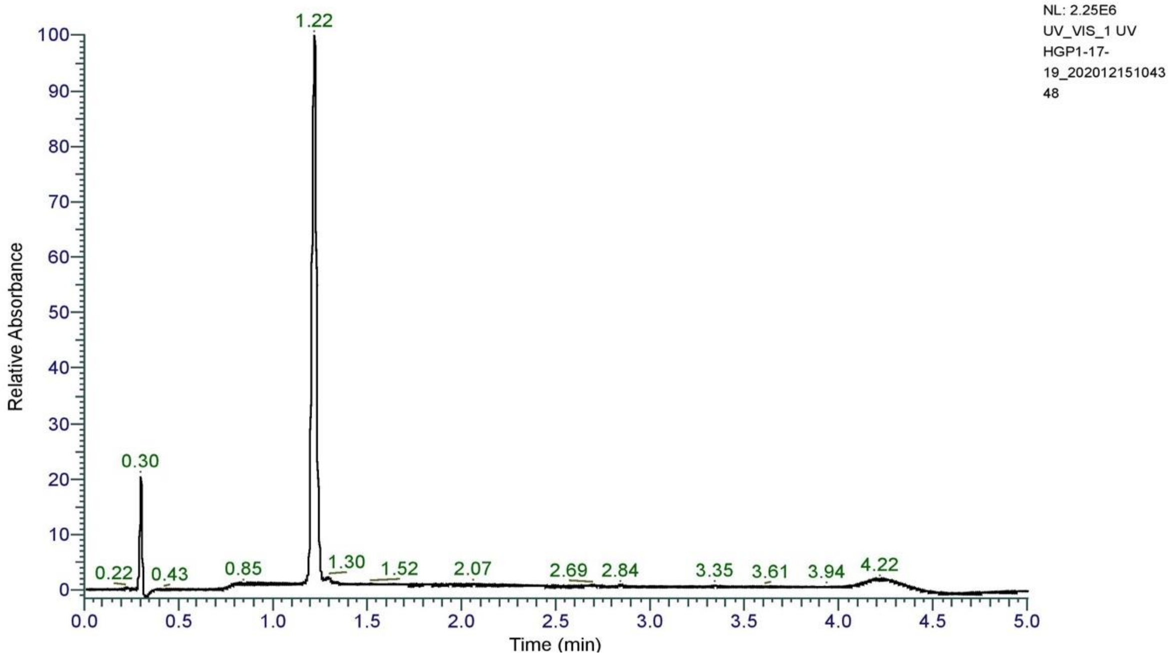

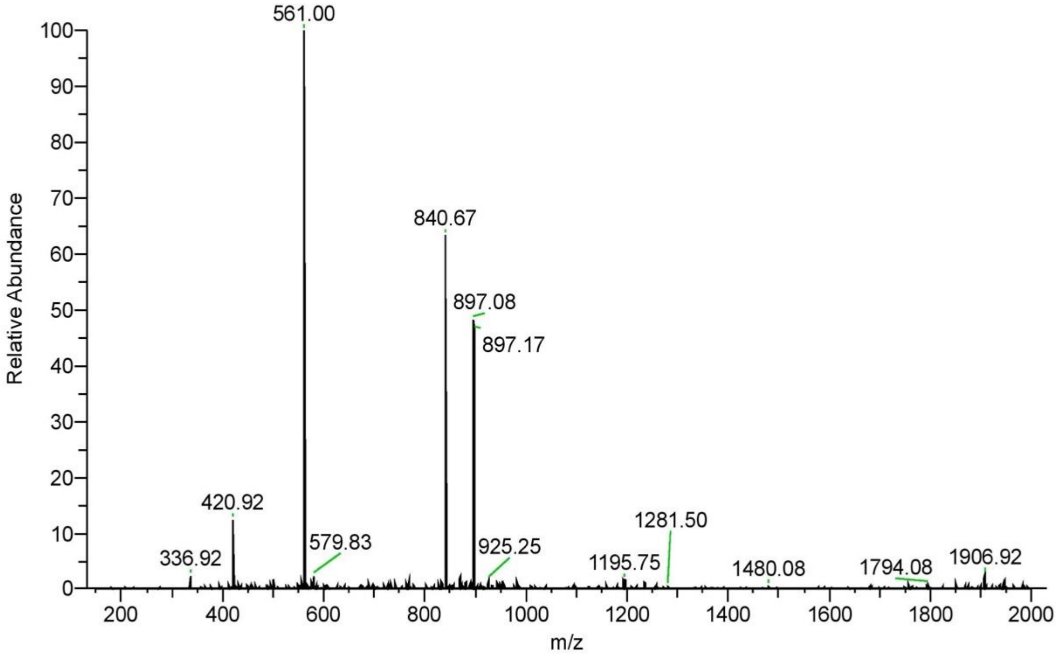

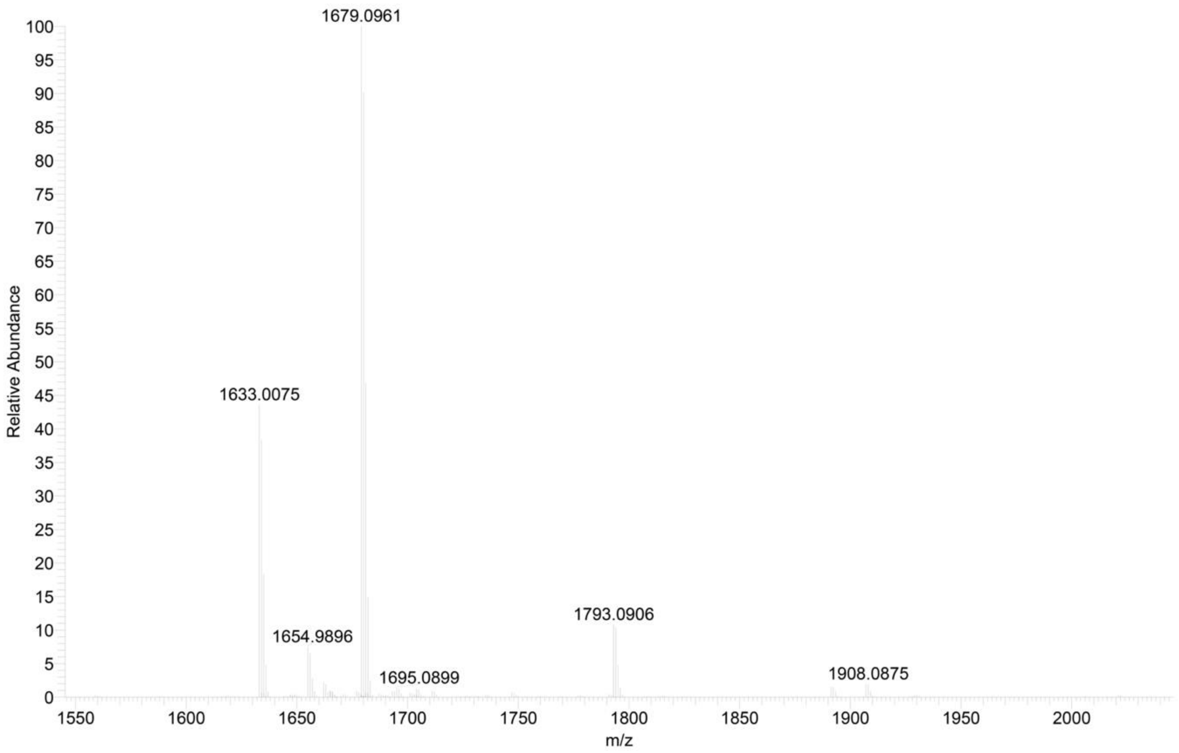
1679.0961
1633.0075
1654.9896
1695.0899
1793.0906
1908.0875
Relative Abundance
m/z

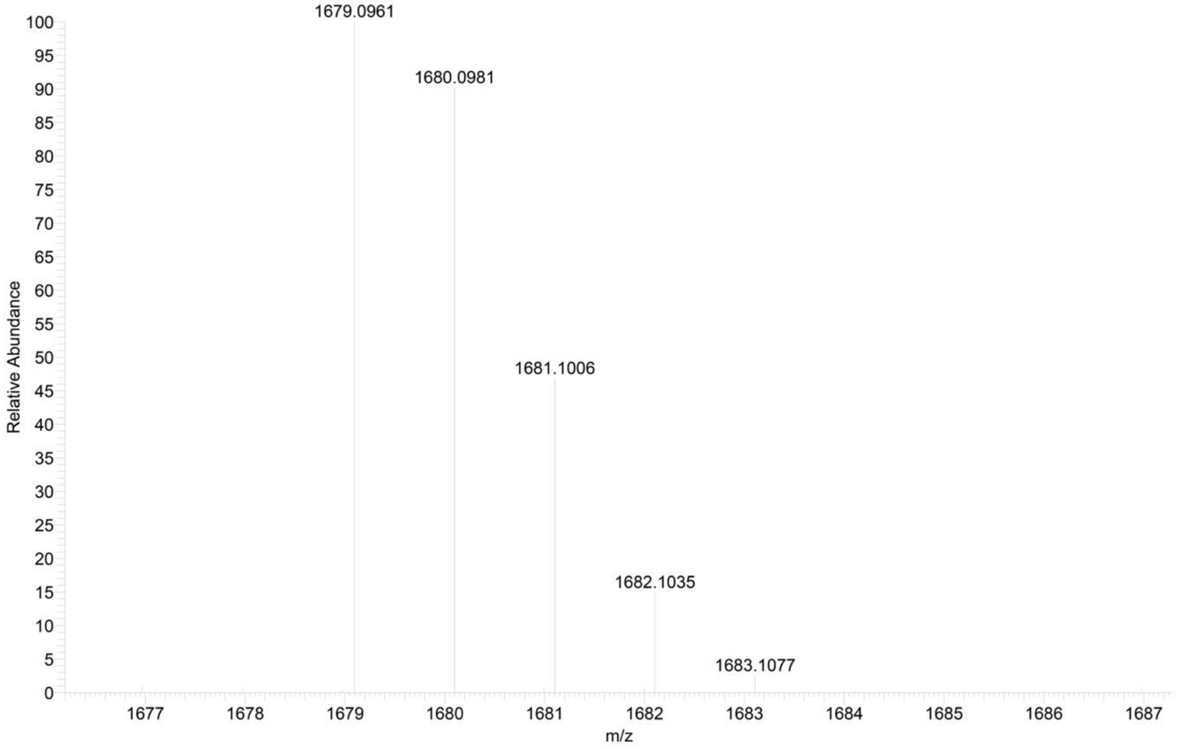
1679.0961
1680.0981
1681.1006
1682.1035
1683.1077
Relative Abundance
m/z

GP9 (ARKKWRKRLKKLKI-NH_2) was obtained from Rink Amide AM resin LL (400 mg, 0.26 mmol/g), the peptide was obtained as a white foamy solid after preparative RP-HPLC purification (311.1 mg, 15.7%). Analytical RP-HPLC: t_R = 1.22 min (100% A to 100% D in 3.5 min, λ= 214 nm). MS (ESI+): $C_{86}H_{159}N_{31}O_{14}$ calc./obs. 1850.27/1850.26 $[M]^+$.

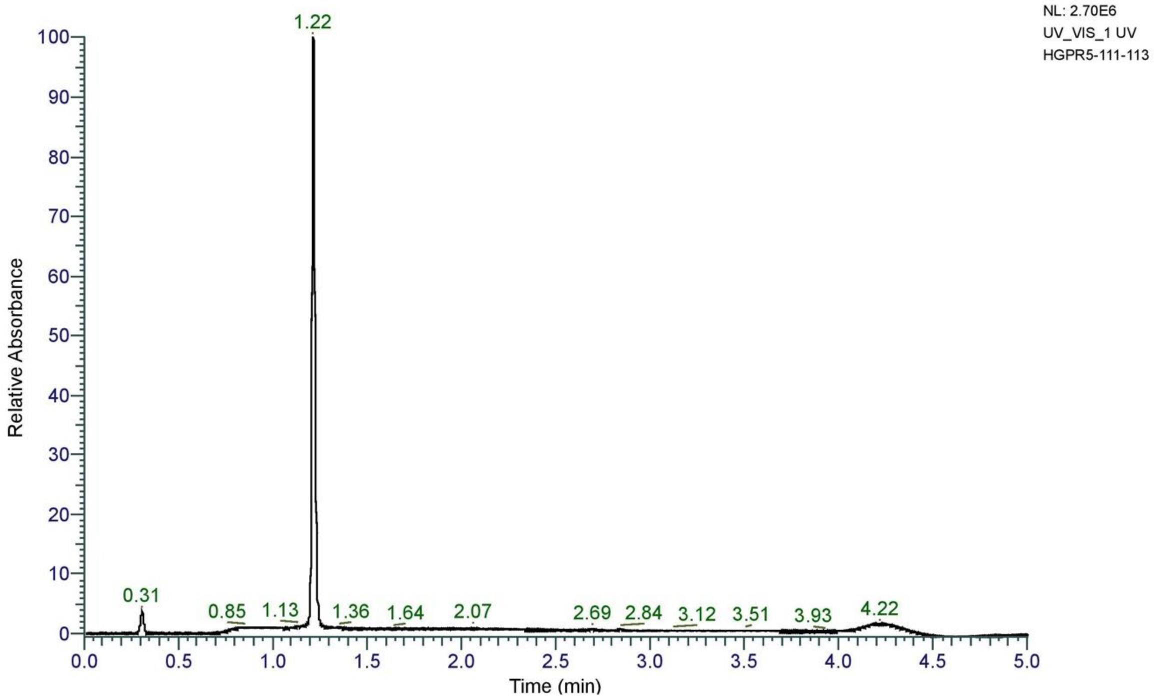

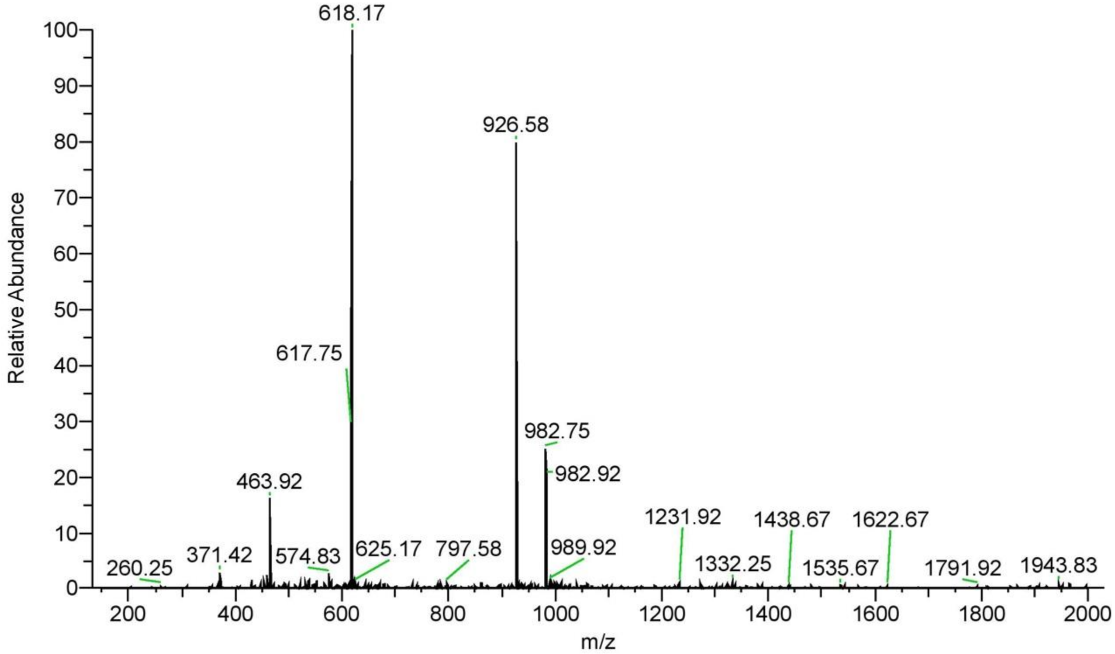

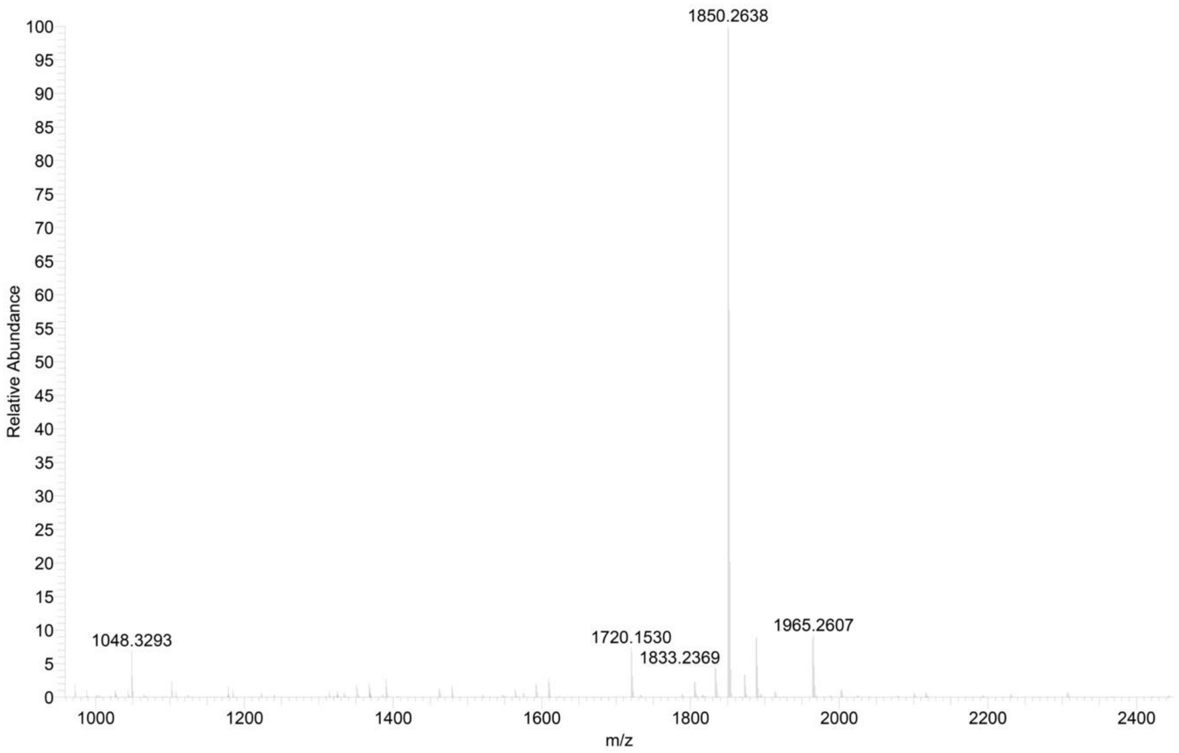
1850.2638
1048.3293
1720.1530
1833.2369
1965.2607
Relative Abundance
m/z

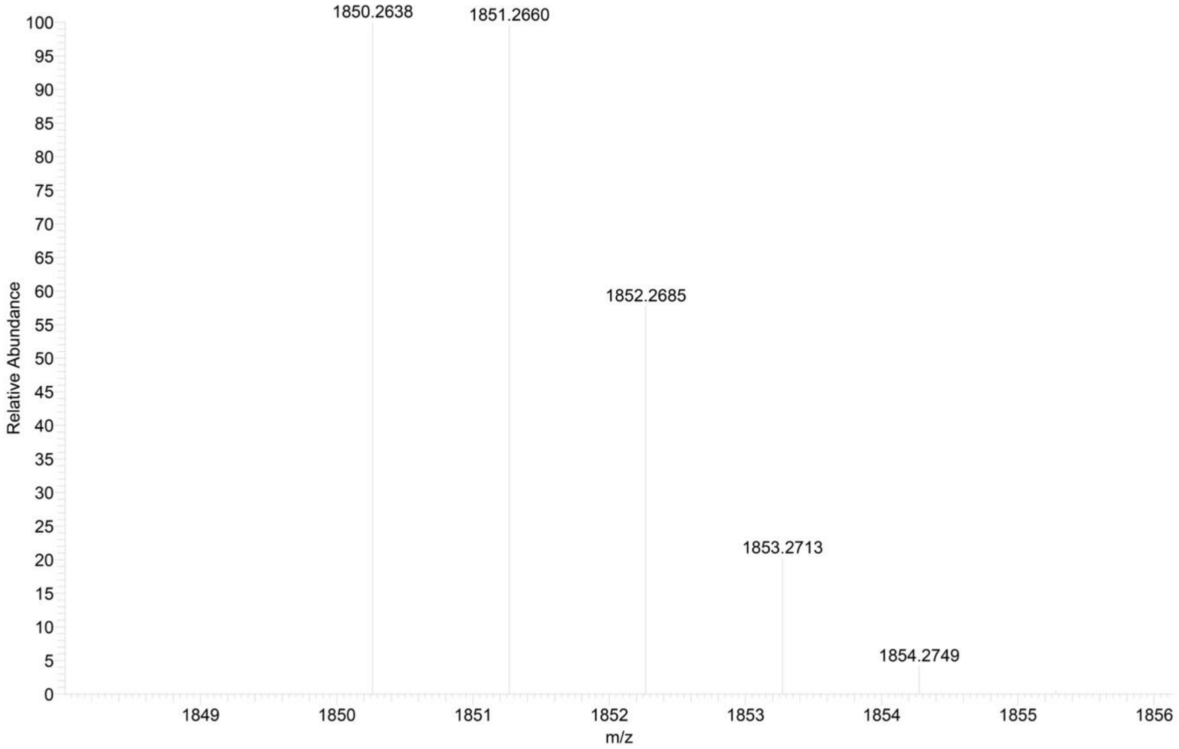
1850.2638
1851.2660
1852.2685
1853.2713
1854.2749
Relative Abundance
m/z

GP10 (AKKVVKKIYKRFQK-NH_2) was obtained from Rink Amide AM resin LL (400 mg, 0.26 mmol/g), the peptide was obtained as a white foamy solid after preparative RP-HPLC purification (278.2 mg, 4.9%). Analytical RP-HPLC: t_R = 1.16 min (100% A to 100% D in 3.5 min, λ= 214 nm). MS (ESI+): $C_{84}H_{147}N_{25}O_{16}$ calc./obs. 1762.15/1762.15 $[M]^+$.

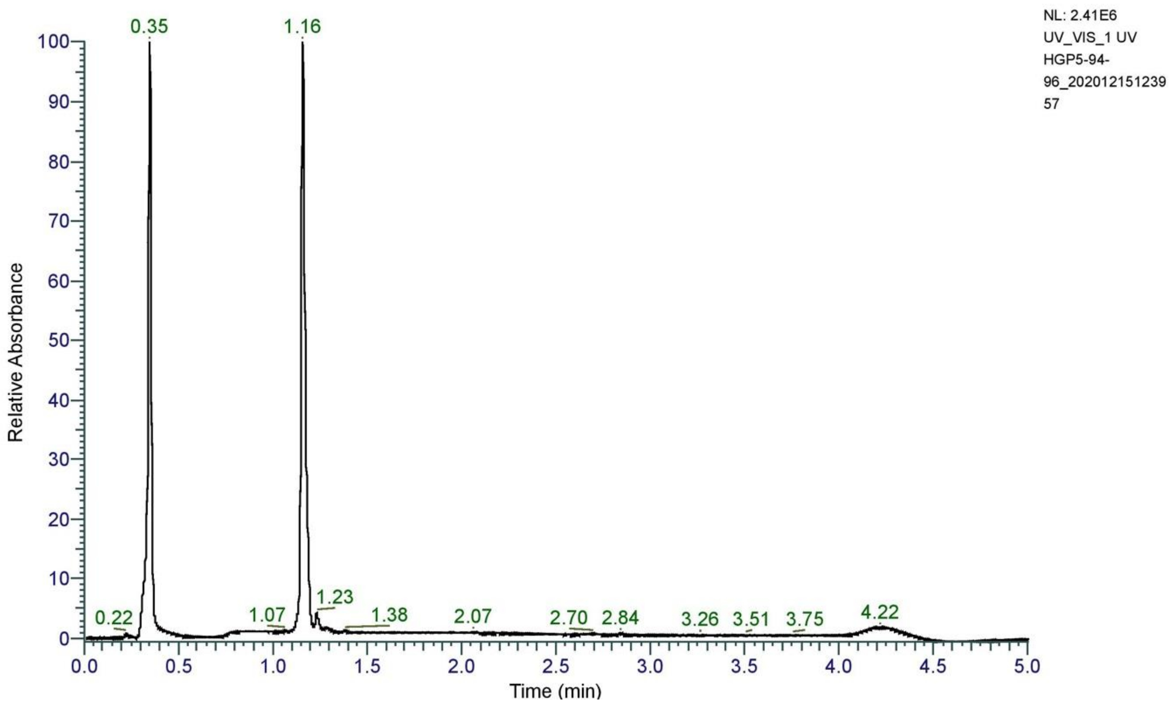

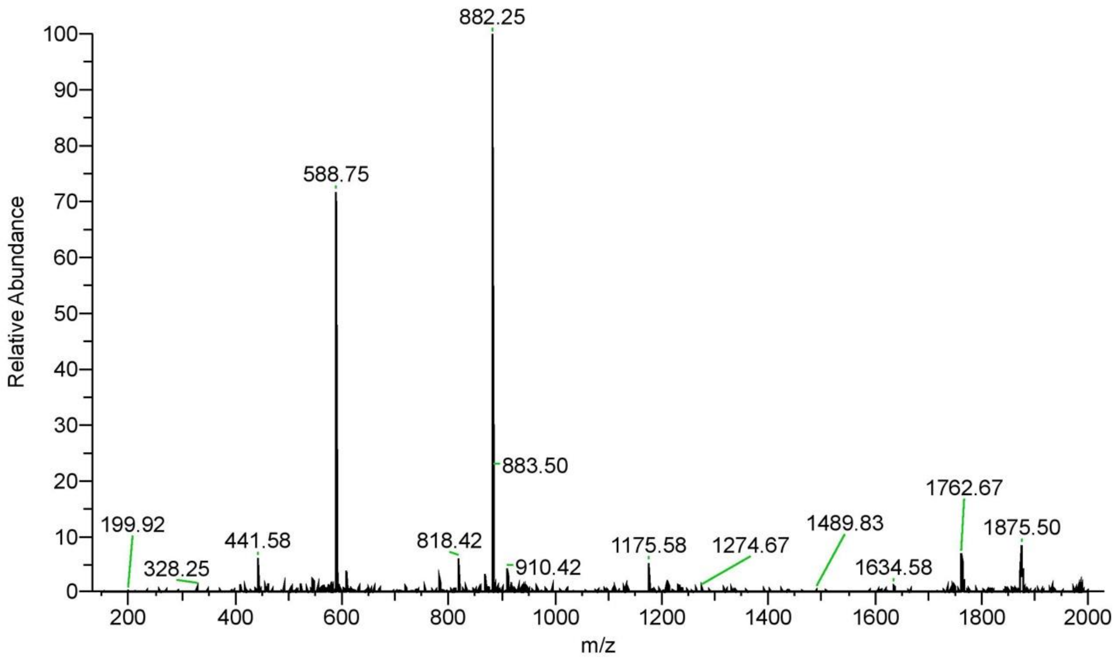

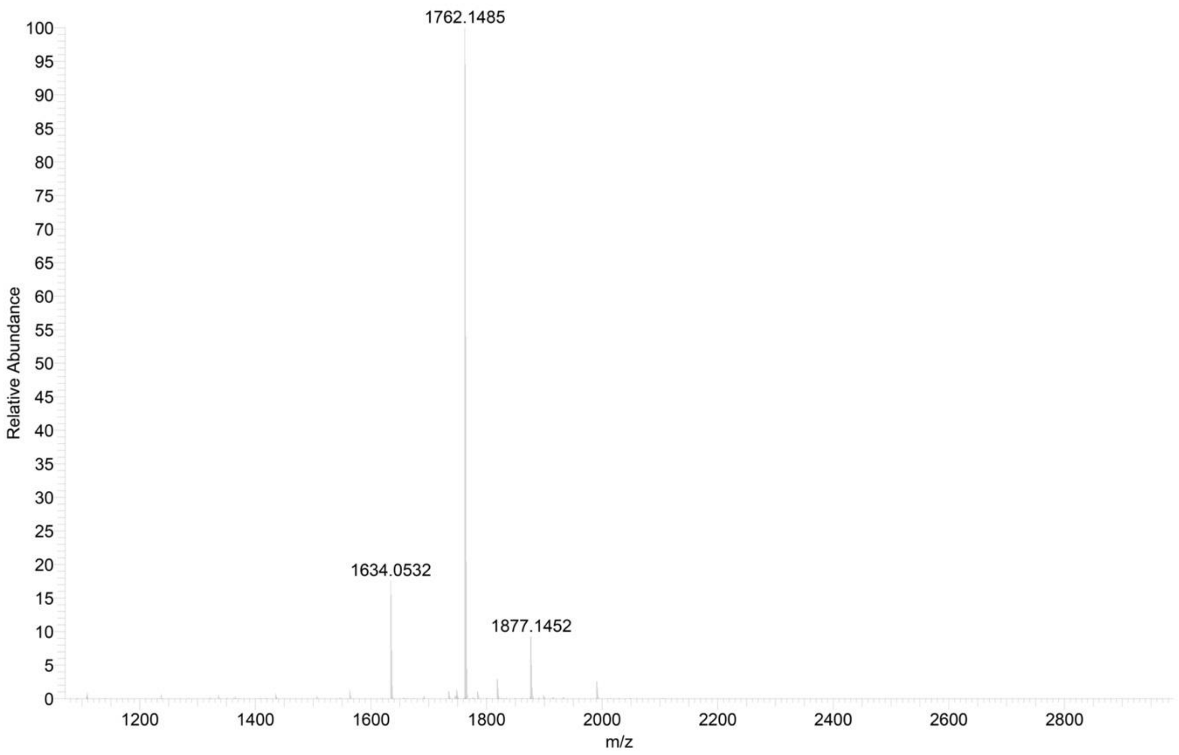
1762.1485
1634.0532
1877.1452
Relative Abundance
m/z
100
95
90
85
80
75
70
65
60
55
50
45
40
35
30
25
20
15
10
5
0
1200
1400
1600
1800
2000
2200
2400
2600
2800

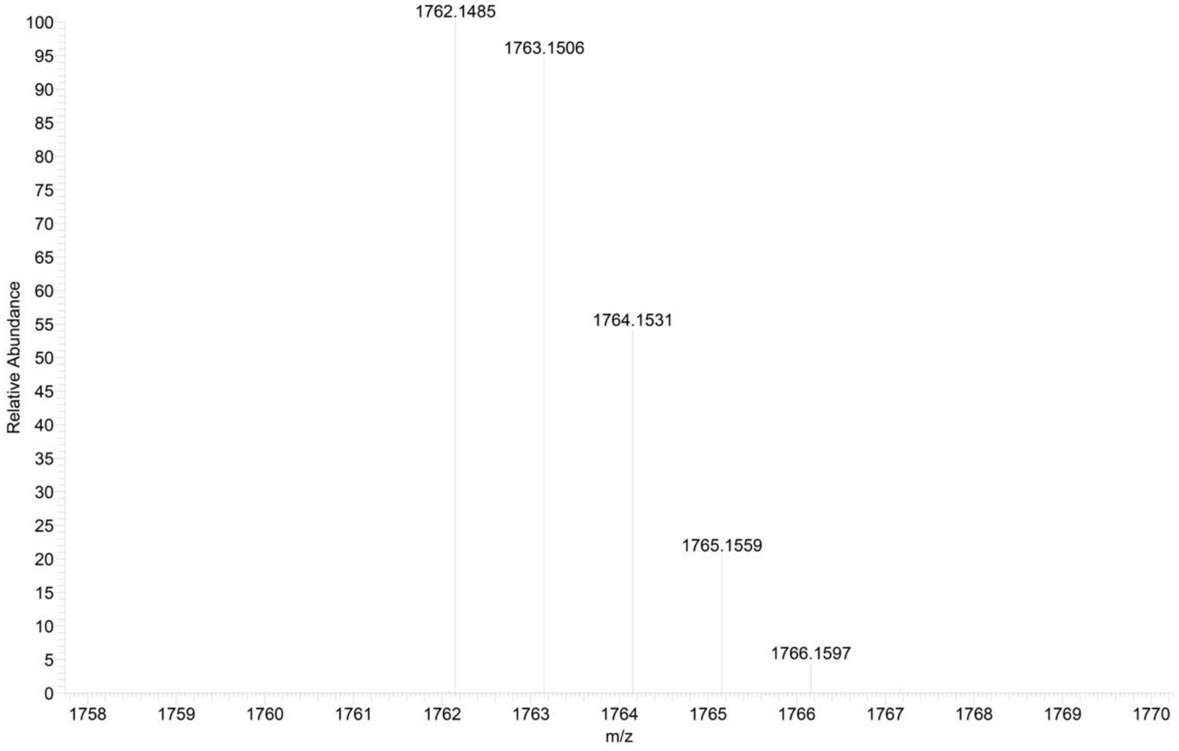
1762.1485
1763.1506
1764.1531
1765.1559
1766.1597
Relative Abundance
m/z
100
95
90
85
80
75
70
65
60
55
50
45
40
35
30
25
20
15
10
5
0
1758
1759
1760
1761
1762
1763
1764
1765
1766
1767
1768
1769
1770

GP11 (ARKFRRLVKKLR-NH_2) was obtained from Rink Amide AM resin LL (400 mg, 0.26 mmol/g), the peptide was obtained as a white foamy solid after preparative RP-HPLC purification (246.3 mg, 4.5%). Analytical RP-HPLC: t_R = 1.21 min (100% A to 100% D in 3.5 min, λ= 214 nm). MS (ESI+): $C_{71}H_{132}N_{28}O_{12}$ calc./obs. 1569.06/1569.05 $[M]^+$.

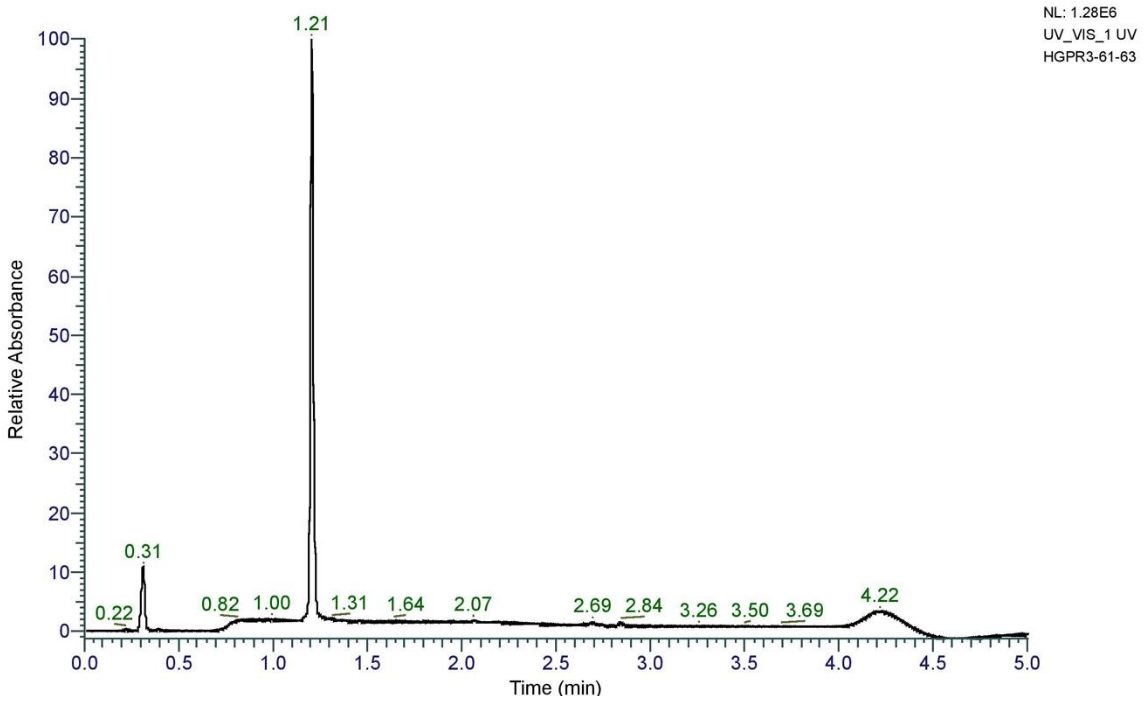

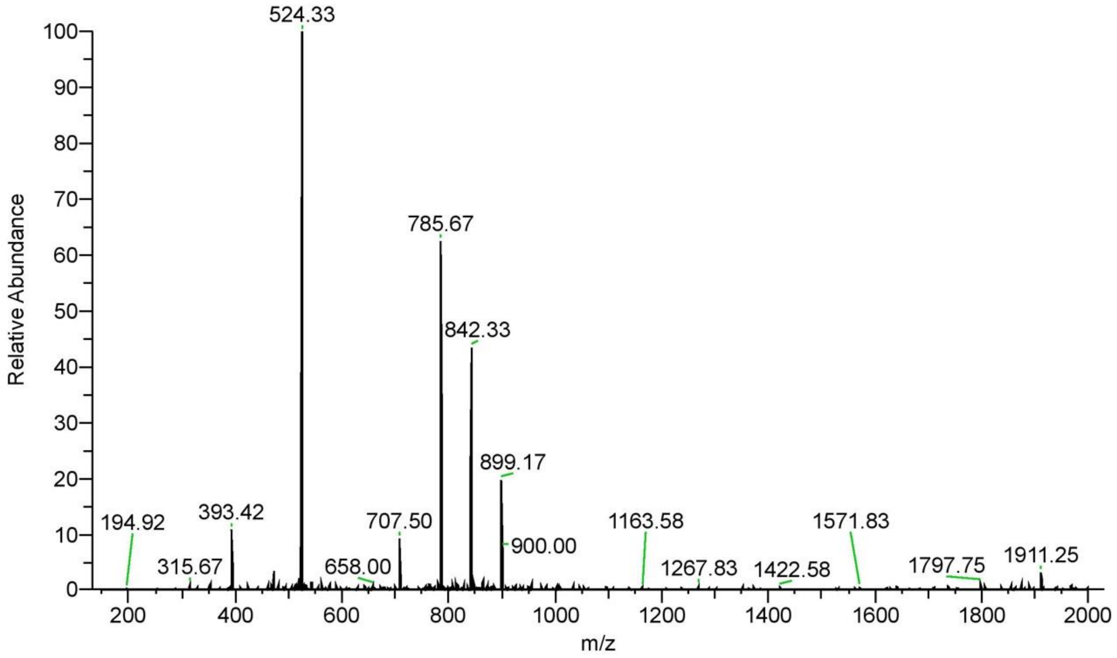

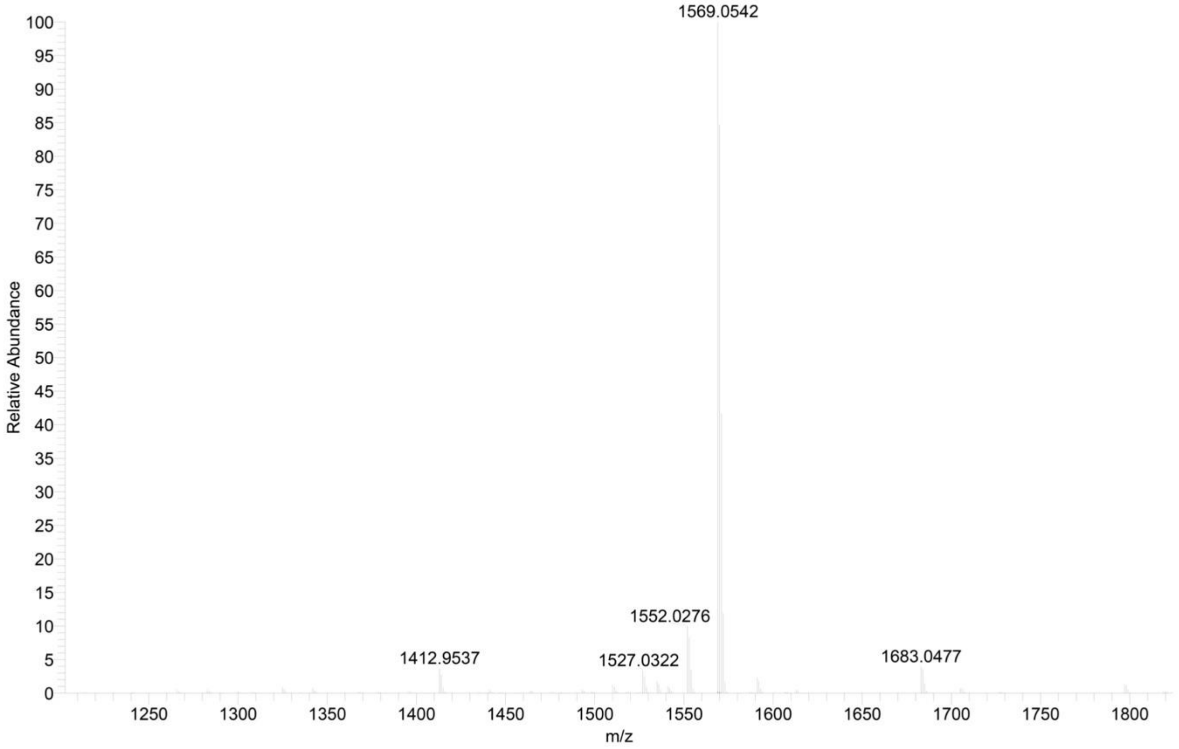

1569.0542
1552.0276
1412.9537
1527.0322
1683.0477
Relative Abundance
m/z

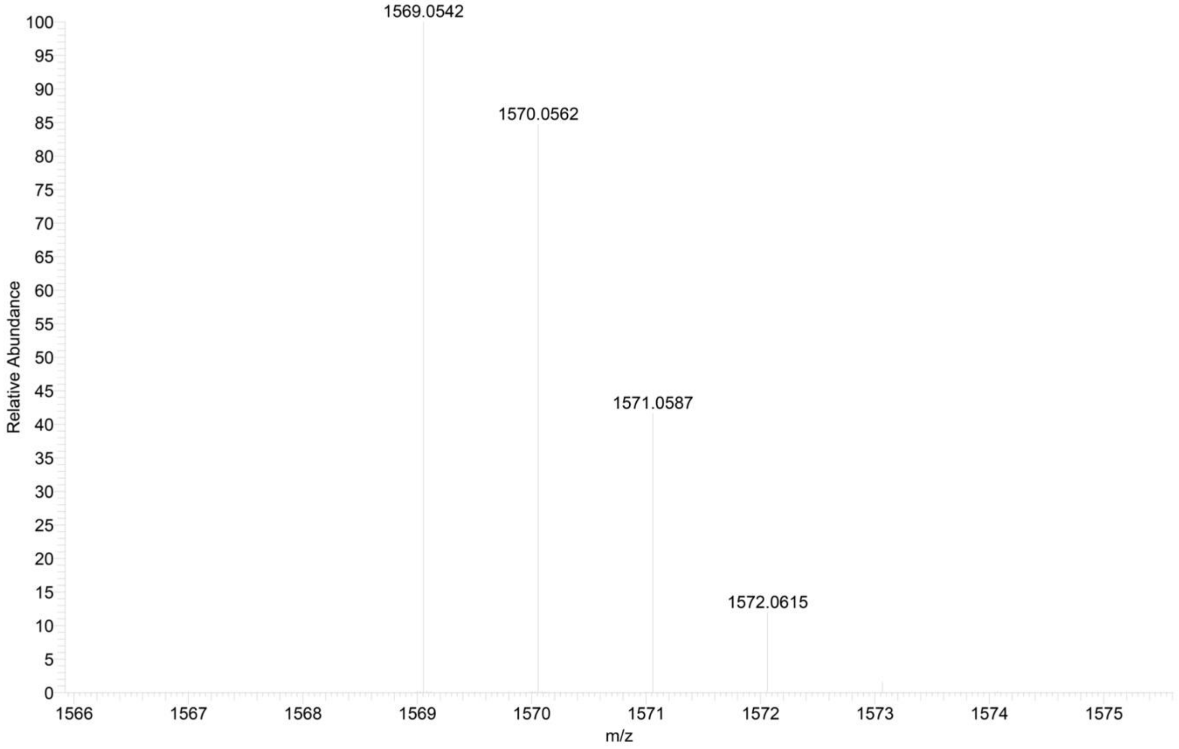

1569.0542
1570.0562
1571.0587
1572.0615
Relative Abundance
m/z

GP12 (LRKARRLVKKLA-NH_2) was obtained from Rink Amide AM resin LL (400 mg, 0.26 mmol/g), the peptide was obtained as a white foamy solid after preparative RP-HPLC purification (233.9 mg, 10.9%). Analytical RP-HPLC: t_R = 1.18 min (100% A to 100% D in 3.5 min, λ= 214 nm). MS (ESI+): $C_{65}H_{127}N_{25}O_{12}$ calc./obs. 1450.01/1450.01 $[M]^+$.

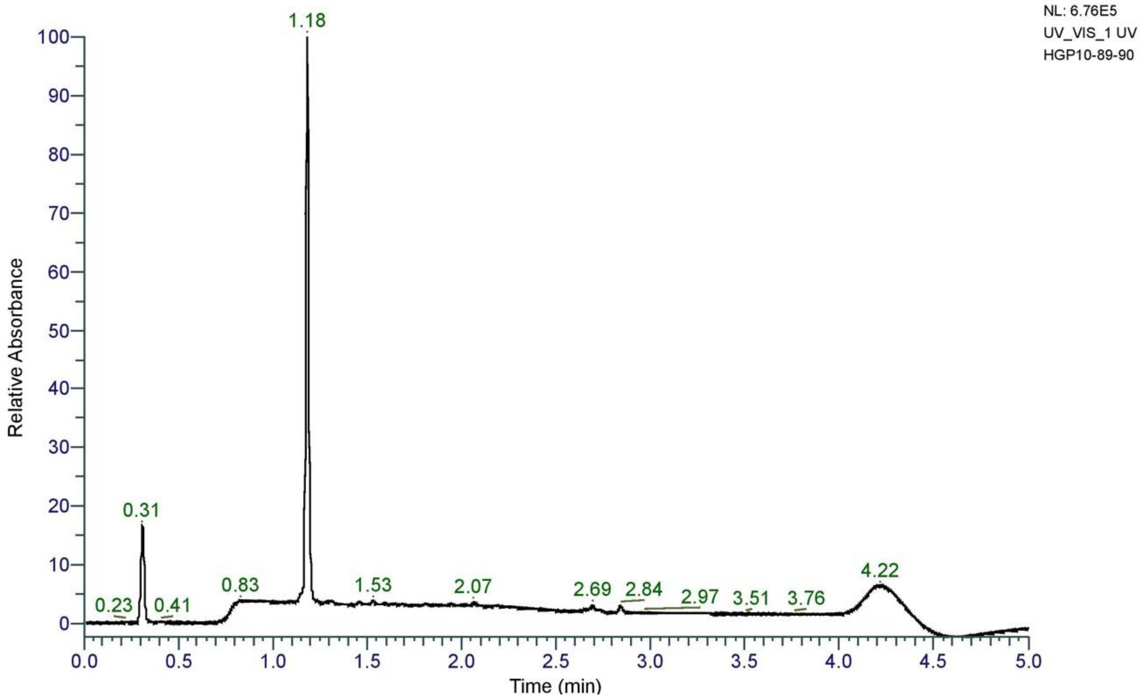

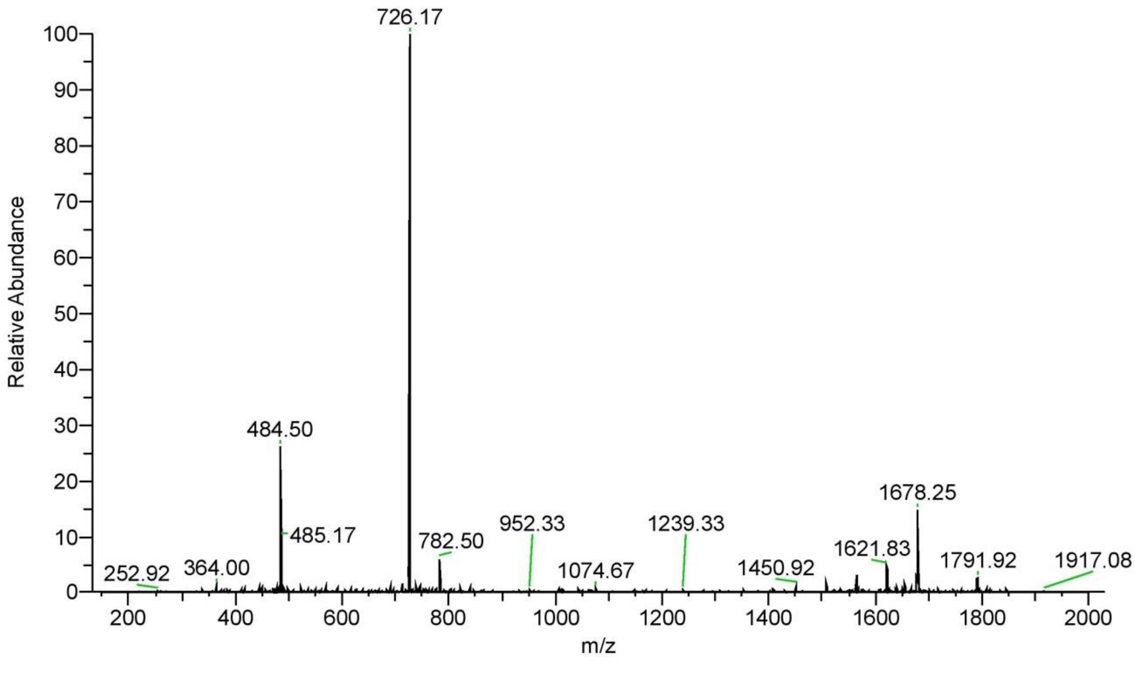

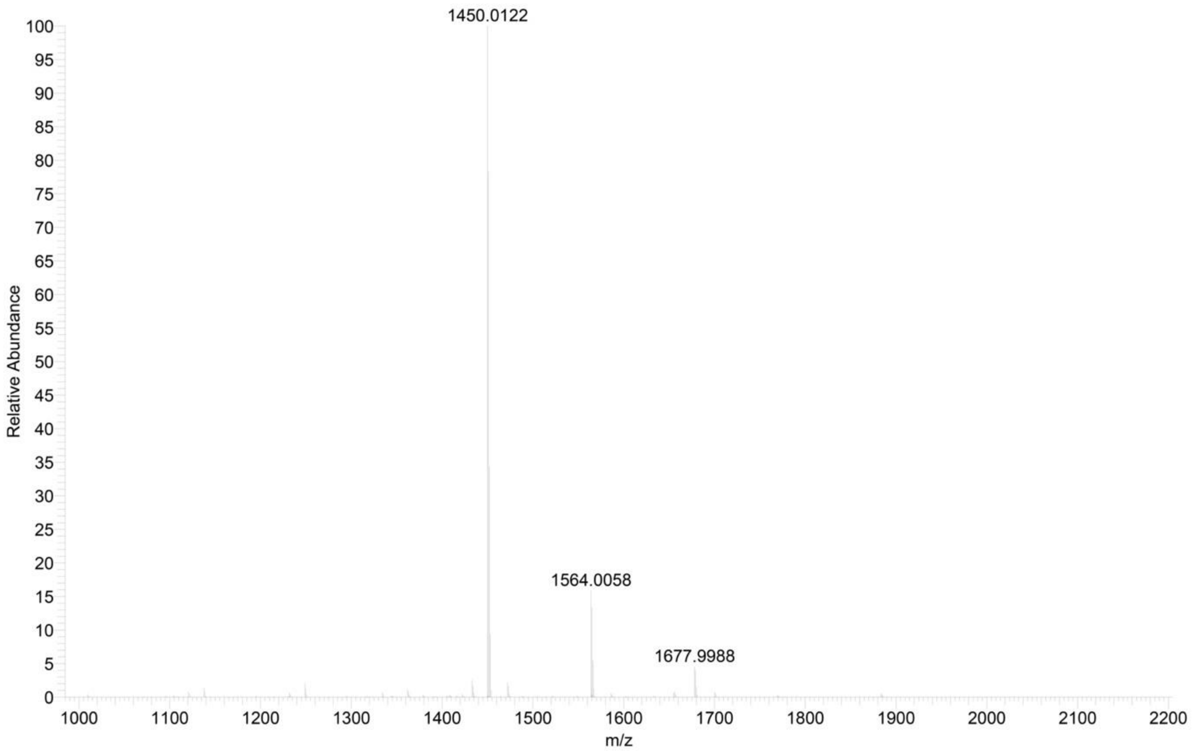
1450.0122
1564.0058
1677.9988
Relative Abundance
m/z
0
5
10
15
20
25
30
35
40
45
50
55
60
65
70
75
80
85
90
95
100
1000
1100
1200
1300
1400
1500
1600
1700
1800
1900
2000
2100
2200

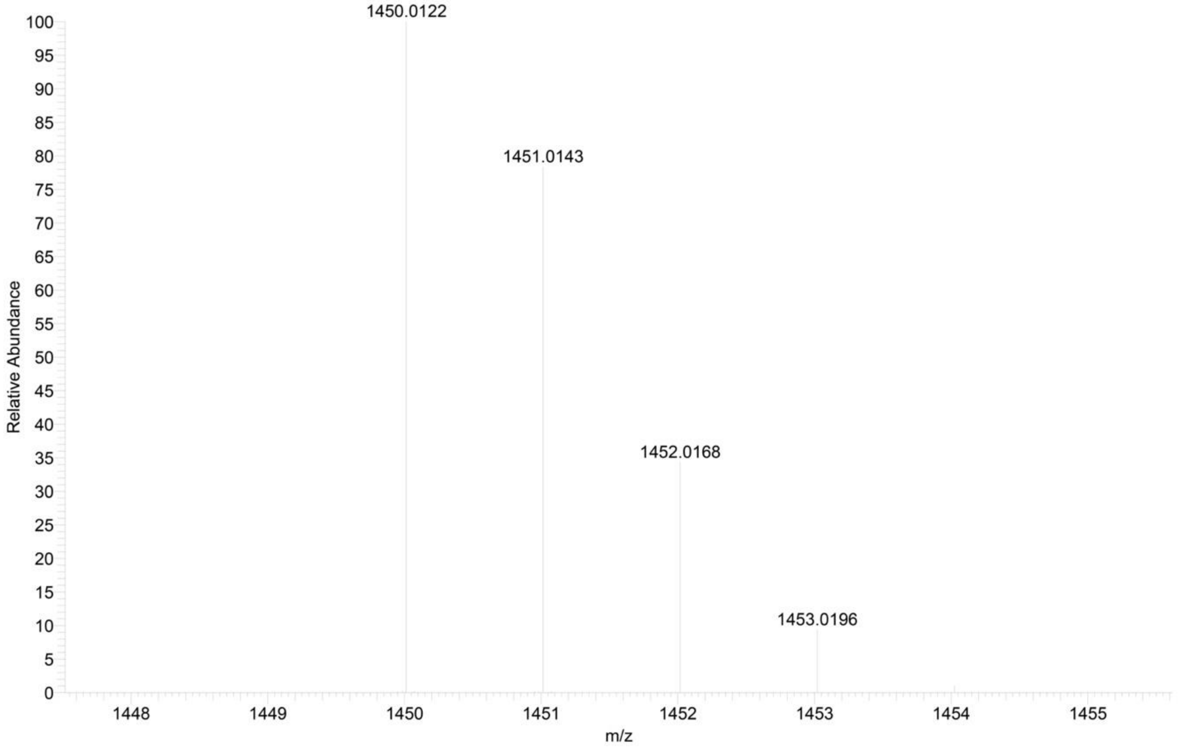
1450.0122
1451.0143
1452.0168
1453.0196
Relative Abundance
m/z
0
5
10
15
20
25
30
35
40
45
50
55
60
65
70
75
80
85
90
95
100
1448
1449
1450
1451
1452
1453
1454
1455

GP13 (KRLWKIRQRIAK-NH_2) was obtained from Rink Amide AM resin LL (400 mg, 0.26 mmol/g), the peptide was obtained as a white foamy solid after preparative RP-HPLC purification (248.9 mg, 34.3%). Analytical RP-HPLC: t_R = 1.18 min (100% A to 100% D in 3.5 min, λ= 214 nm). MS (ESI+): $C_{73}H_{131}N_{27}O_{13}$ calc./obs. 1594.04/1594.04 $[M]^+$.

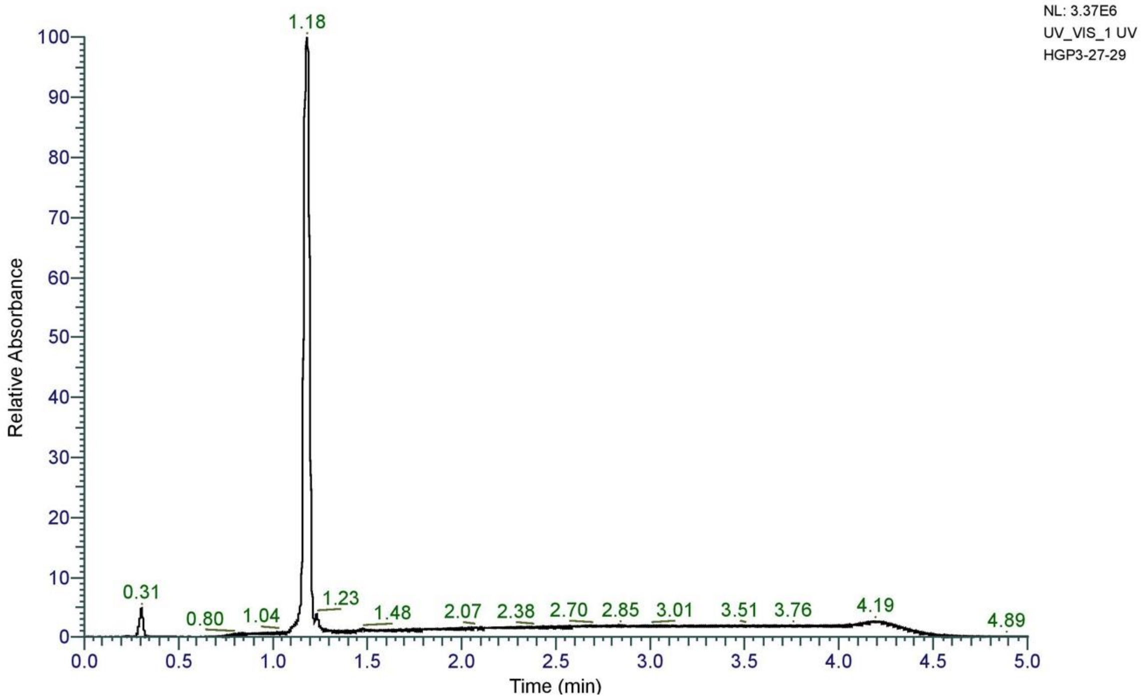

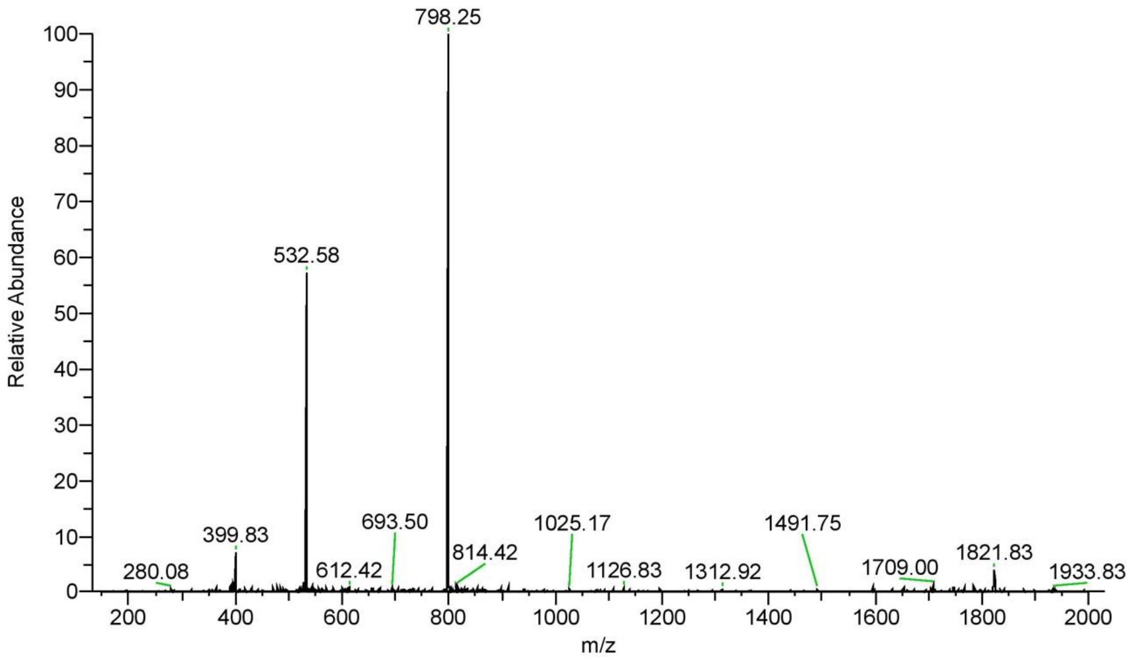

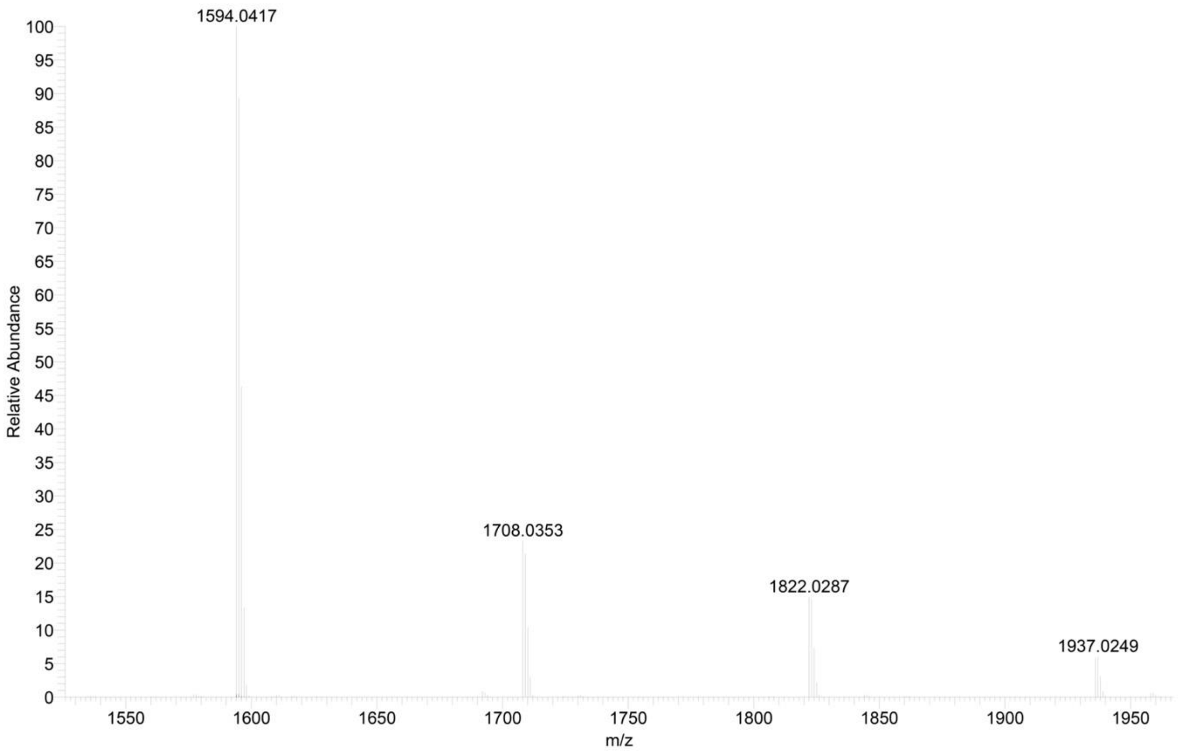
1594.0417
1708.0353
1822.0287
1937.0249
Relative Abundance
m/z

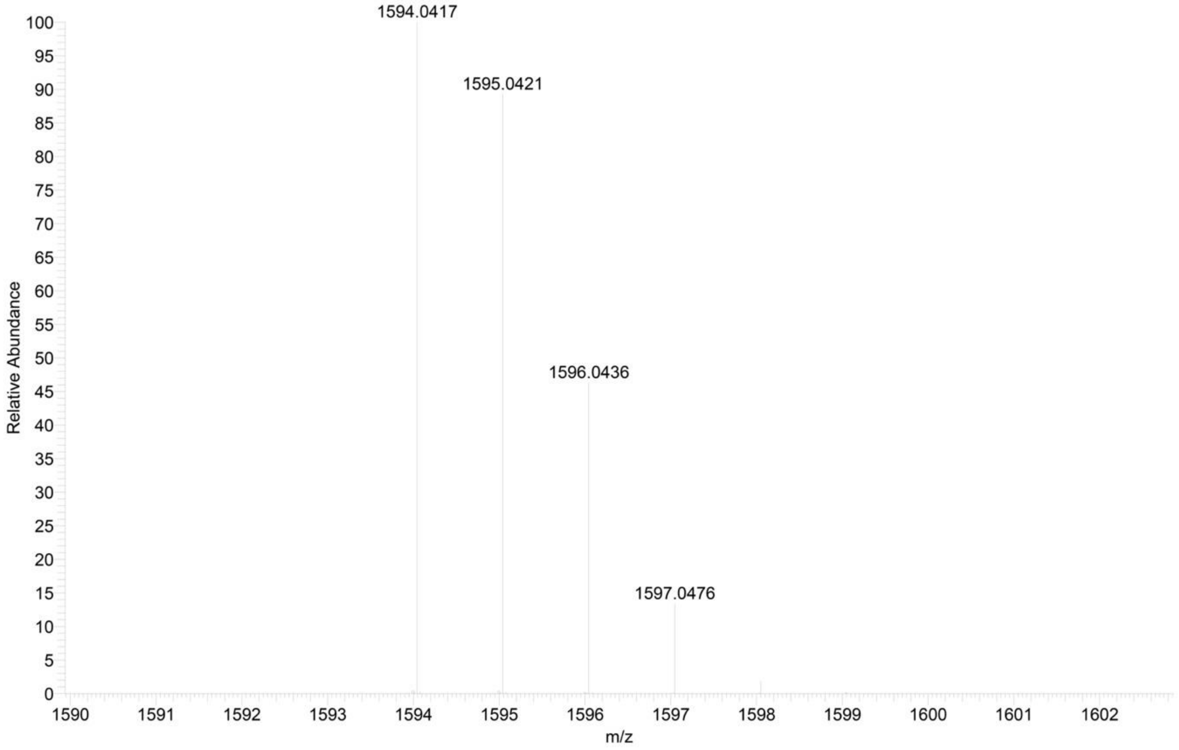
1594.0417
1595.0421
1596.0436
1597.0476
Relative Abundance
m/z

GP14 (LNALKKVFQKIH-NH_2) was obtained from Rink Amide AM resin LL (400 mg, 0.26 mmol/g), the peptide was obtained as a white foamy solid after preparative RP-HPLC purification (208.8 mg, 7.8%). Analytical RP-HPLC: t_R = 1.39 min (100% A to 100% D in 3.5 min, λ= 214 nm). MS (ESI+): $C_{68}H_{116}N_{20}O_{14}$ calc./obs. 1436.9/1437.91 $[M]^+$.

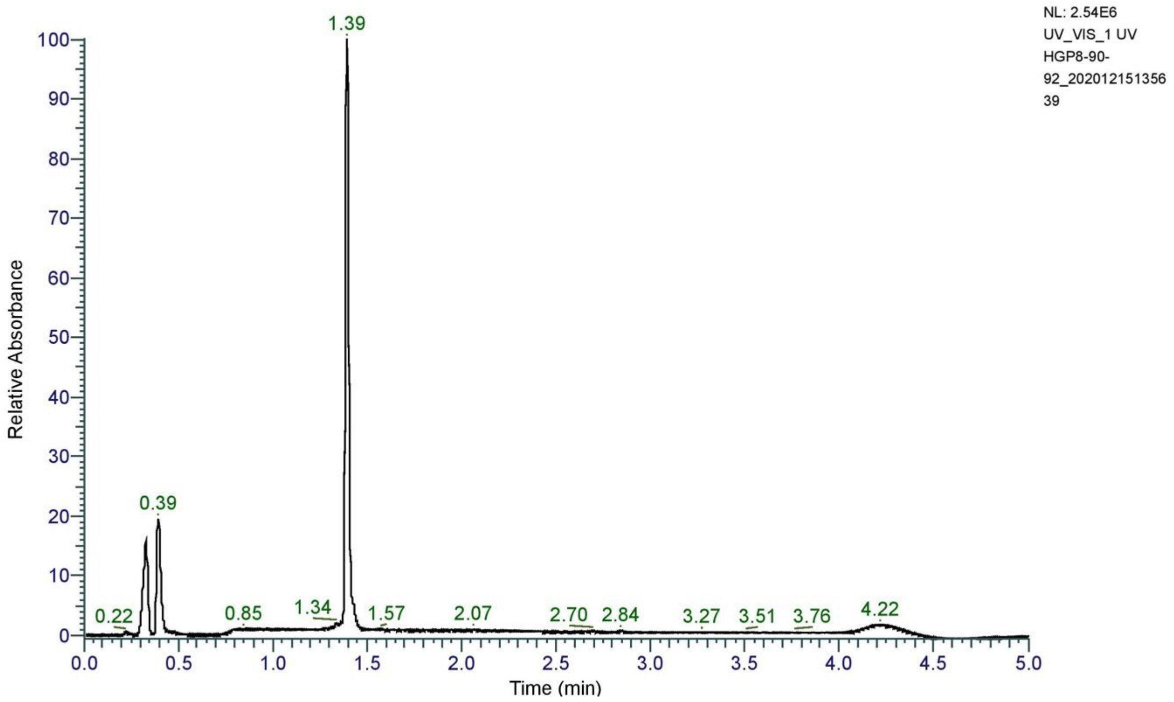

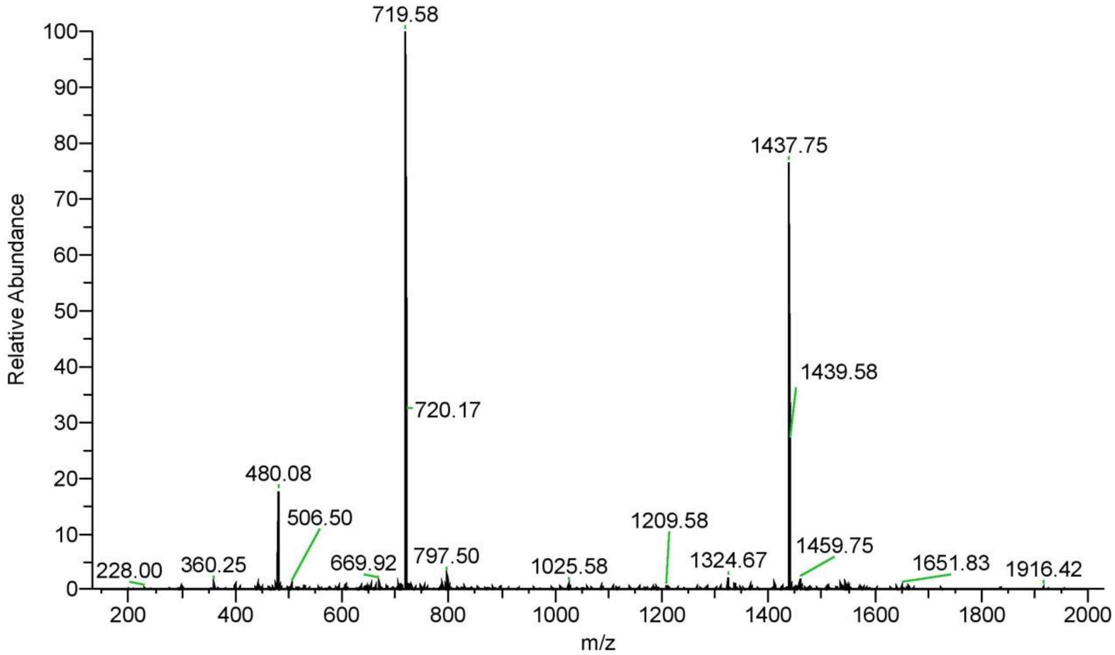

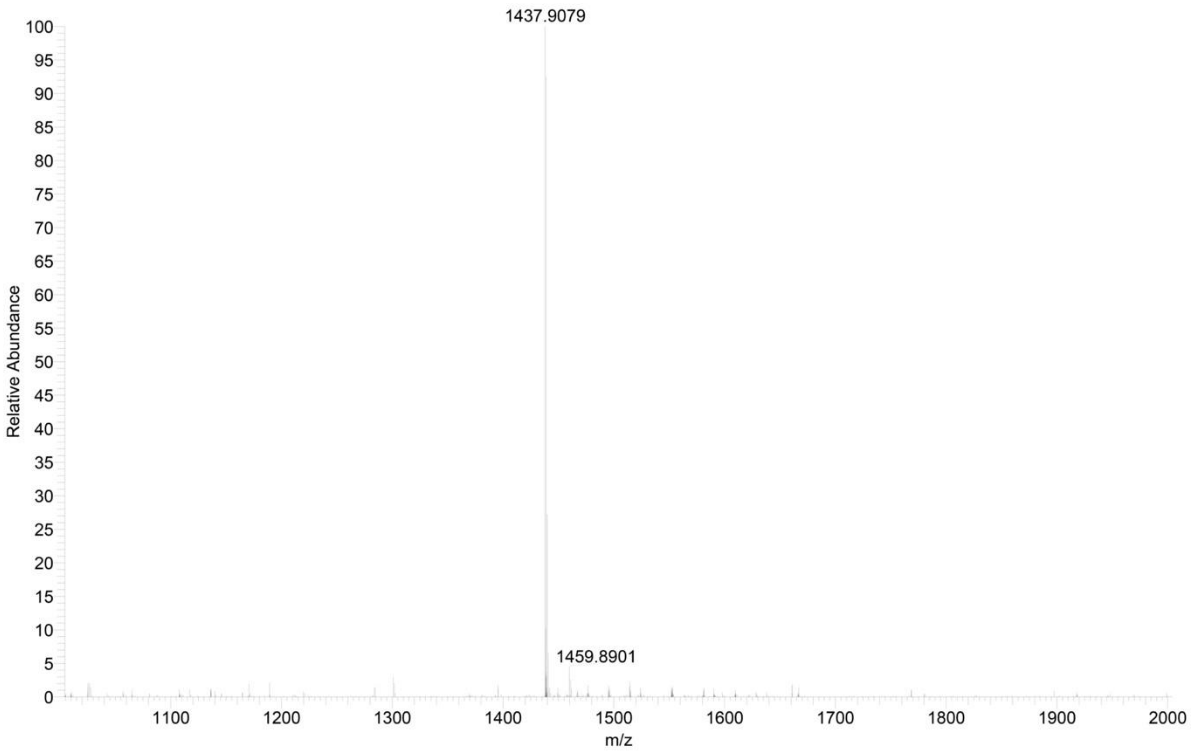
1437.9079
1459.8901
Relative Abundance
m/z

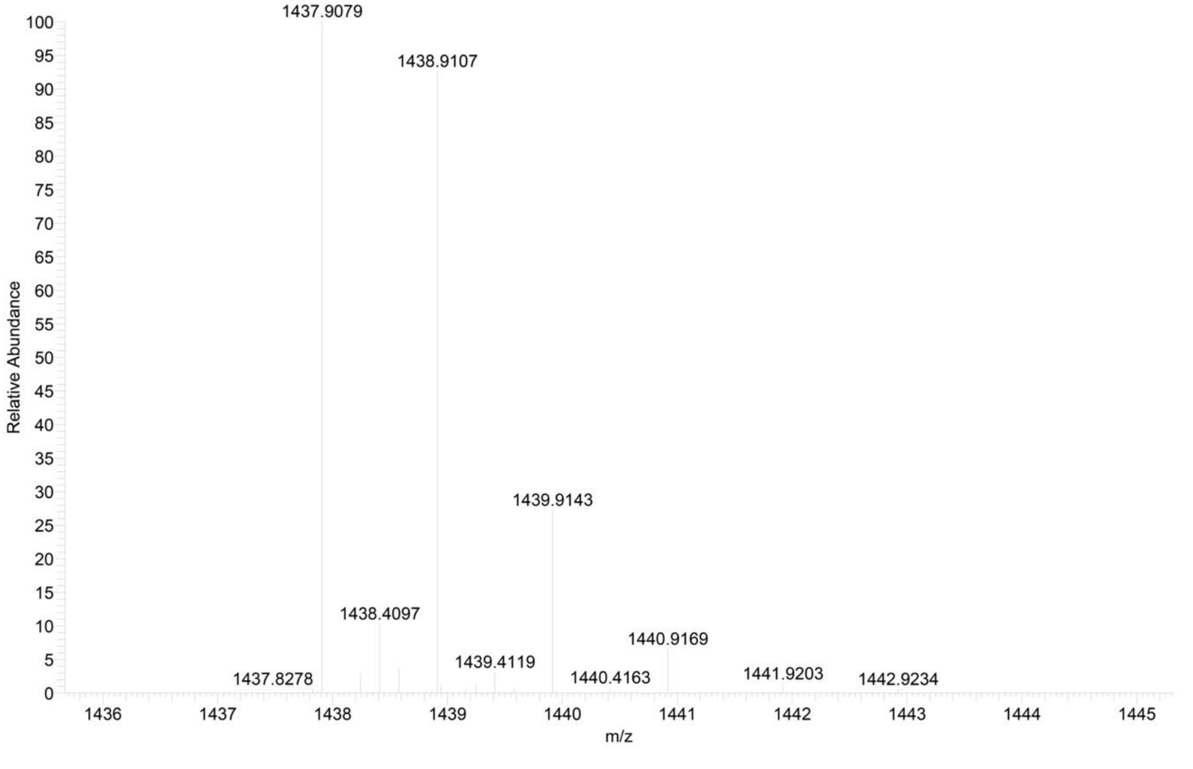
1437.9079
1438.9107
1439.9143
1438.4097
1440.9169
1439.4119
1437.8278
1440.4163
1441.9203
1442.9234
Relative Abundance
m/z

Supporting information Note 4: Topology of the DPC molecule

```
; Charge from Chiu et al.
; Chiu, S. W.; Clark, M.; Balaji, V.; Subramaniam, S.; Scott, H. L.; Jakobsson, E.
Incorporation of surface tension into molecular dynamics simulation of an interface: a fluid
phase lipid bilayer membrane. Biophys. J. 1995, 69, 1230-1245.
; Atom types from GROMOS53A6
; Oostenbrink, C.; Soares, T. A.; van der Vegt, N. F. A.; van Gunsteren, W. F. Validation of
the 53A6 GROMOS force field. Eur. Biophys. J. 2005, 34, 273-284.

[ moleculetype ]
; Name   nrexcl
DPC      3

[ atoms ]
;   nr    type   resnr  residu    atom    cgnr        charge          mass
     1     CH3       1    DPC      C1       1         0.40  15.035  ; qtot: 0.25
     2     CH3       1    DPC      C2       2         0.40  15.035  ; qtot: 0.50
     3     CH3       1    DPC      C3       3         0.40  15.035 ; qtot: 0.75
     4     NL        1    DPC      N4       4        -0.5  14.0067 ; qtot: 0.75
     5     CH2       1    DPC      C5       5         0.30  14.027 ; qtot:  1.0
     6     CH2       1    DPC      C6       6         0.40  14.027 ; qtot: 1.0
     7      OA       1    DPC      O7       7        -0.80  15.999 ; qtot: 0.64
     8       P       1    DPC      P8       8         1.7  30.973 ; qtot : 1.63
     9      OM       1    DPC      O9       9        -0.8 15.999 ; qtot: 0.995
    10      OM       1    DPC     O10      10        -0.8 15.999 ; qtot: 0.36
    11      OA       1    DPC     O11      11        -0.7  15.999 ; qtot: 0.0
    12     CH2       1    DPC     C12      12         0.0   14.027  ; qtot: 0
    13     CH2       1    DPC     C13      13         0.0   14.027  ; qtot: 0
    14     CH2       1    DPC     C14      14         0.0   14.027  ; qtot: 0
    15     CH2       1    DPC     C15      15         0.0   14.027  ; qtot: 0
    16     CH2       1    DPC     C16      16         0.0   14.027  ; qtot: 0
    17     CH2       1    DPC     C17      17         0.0   14.027  ; qtot: 0
    18     CH2       1    DPC     C18      18         0.0   14.027  ; qtot: 0
    19     CH2       1    DPC     C19      19         0.0   14.027  ; qtot: 0
    20     CH2       1    DPC     C20      20         0.0   14.027  ; qtot: 0
    21     CH2       1    DPC     C21      21         0.0   14.027  ; qtot: 0
    22     CH2       1    DPC     C22      22         0.0   14.027  ; qtot: 0
    23     CH3       1    DPC     C23      23         0.0   15.035  ; qtot: 0


[ bonds ]
;  ai    aj funct            c0            c1            c2            c3

    1     4     2    gb_21
    2     4     2    gb_21
    3     4     2    gb_21
    4     5     2    gb_21
    5     6     2    gb_27
    6     7     2    gb_18
    7     8     2    gb_28
    8     9     2    gb_24
    8    10     2    gb_24
    8    11     2    gb_28
   11    12     2    gb_18
   12    13     2    gb_27
   13    14     2    gb_27
   14    15     2    gb_27
   15    16     2    gb_27
   16    17     2    gb_27
   17    18     2    gb_27
   18    19     2    gb_27
   19    20     2    gb_27
   20    21     2    gb_27
   21    22     2    gb_27
   22    23     2    gb_27

[ pairs ]
;  ai    aj funct
    1     6     1
    2     6     1
    3     6     1
    4     7     1
    5     8     1
    6     9     1
```

```
     6    10     1
     6    11     1
     7    12     1
     8    13     1
     9    12     1
    10    12     1
    11    14     1
;   12    15     1
;   13    16     1
;   14    17     1
;   15    18     1
;   16    19     1
;   17    20     1
;   18    21     1
;   19    22     1
;   20    23     1

[ angles ]
;  ai    aj    ak funct
     1     4     2     2    ga_13
     1     4     3     2    ga_13
     1     4     5     2    ga_13
     2     4     3     2    ga_13
     2     4     5     2    ga_13
     3     4     5     2    ga_13
     4     5     6     2    ga_15
     5     6     7     2    ga_15
     6     7     8     2    ga_26
     7     8     9     2    ga_14
     7     8    10     2    ga_14
     7     8    11     2    ga_5
     9     8    10     2    ga_29
    10     8    11     1    ga_14
     8    11    12     1    ga_26
    11    12    13     1    ga_15
    12    13    14     1    ga_15
    13    14    15     1    ga_15
    14    15    16     1    ga_15
    15    16    17     1    ga_15
    16    17    18     1    ga_15
    17    18    19     1    ga_15
    18    19    20     1    ga_15
    19    20    21     1    ga_15
    20    21    22     1    ga_15
    21    22    23     1    ga_15

[ dihedrals ]
;  ai    aj    ak    al funct
     1     4     5     6     1 gd_29
     4     5     6     7     1 gd_4
     4     5     6     7     1 gd_36
     5     6     7     8     1 gd_29
;
; define gd_20     0.000       5.09           2
; O-P-O- (dna, lipids)  1.2
     6     7     8     9     1 gd_20
     7     8    11    12     1 gd_27
     8    11    12    13     1 gd_29
    11    12    13    14     1 gd_1
    12    13    14    15     1 gd_34
    13    14    15    16     1 gd_34
    14    15    16    17     1 gd_34
    15    16    17    18     1 gd_34
    16    17    18    19     1 gd_34
    17    18    19    20     1 gd_34
    18    19    20    21     1 gd_34
    19    20    21    22     1 gd_34
    20    21    22    23     1 gd_34
```
